# Regioselective addition of Grignard reagents to *N*-acylpyrazinium salts: synthesis of substituted 1,2-dihydropyrazines and Δ^5^-2-oxopiperazines

**DOI:** 10.3762/bjoc.15.8

**Published:** 2019-01-08

**Authors:** Valentine R St. Hilaire, William E Hopkins, Yenteeo S Miller, Srinivasa R Dandepally, Alfred L Williams

**Affiliations:** 1 Biomanufacturing Research Institute and Technology Enterprise (BRITE), North Carolina Central University, Durham, North Carolina 27707, United States; 2 Department of Pharmaceutical Sciences, North Carolina Central University, Durham, North Carolina 27707, United States

**Keywords:** *N*-acylpyrazinium salts, 1,2-dihydropyrazines, Grignard reagents, Δ^5^-2-oxopiperazines, regioselective addition

## Abstract

The regioselective addition of Grignard reagents to mono- and disubstituted *N*-acylpyrazinium salts affording substituted 1,2-dihydropyrazines in modest to excellent yields (45–100%) is described. Under acidic conditions, these 1,2-dihydropyrazines can be converted to substituted Δ^5^-2-oxopiperazines providing a simple and efficient approach towards their preparation.

## Introduction

Pyrazine and piperazine ring systems are key structural elements in a large number of biologically active molecules [1–6]. Compounds containing these scaffolds were shown to behave as anticancer agents [2–5], sodium channel blockers [5], and also display antiviral activity [7]. Due to their appearance in an array of biologically active small molecules and natural products, efficient synthetic routes into this class of privileged structures would be very beneficial [7–8]. One approach towards their synthesis involves the addition of nucleophiles to activated pyrazines. We recently showed that 3-alkoxy-substituted *N*-acylpyrazinium salts can be selectively reduced by tributyltin hydride to afford 1,2-dihydropyrazines in good to excellent yields [9]. There have been other reports involving the addition of TMS-ketene acetals to pyrazinium salts [10–12]. A double nucleophilic addition of bis(trimethylsilyl)ketene acetals to pyrazines activated with methyl chloroformate was found to afford polycyclic γ-lactones in moderate yields [3,10–11]. The work by Garduño-Alva and co-workers demonstrated that these TMS-ketene acetals can be regioselectively added to substituted *N*-triflate pyrazinium salts to also generate γ-lactones [12].

Grignard reagents have been used as nucleophiles on a variety of *N*-acyl-activated pyridines in the production of natural products and biologically active small molecules [13–20]. To our surprise, there are no reports on the nucleophilic addition of Grignard reagents to *N*-acylpyrazinium salts. A literature search showed this organometallic reagent reacting with pyrazine *N*-oxides towards the one-pot synthesis of *N*-Boc-protected *N*-hydroxy-substituted piperazines in good yields [6]. Methylmagnesium iodide was observed adding to 2-cyano-6-morpholinylpyrazine [21]. As a part of our continued exploration into the synthetic utility of *N*-acylpyrazinium salts, herein we report the regioselective addition of Grignard reagents to mono- and disubstituted *N*-acylpyrazinium salts towards the synthesis of 1,2-dihydropyrazines and Δ^5^-2-oxopiperazines.

## Results and Discussion

Our journey began by first reacting 2-methoxypyrazine with phenyl chloroformate to generate the *N*-acylpyrazinium salt **2** using DCM as the solvent. Next, phenylmagnesium bromide in THF was added at −41 °C. After stirring for 60 min, dihydropyrazine **3a** was isolated in a yield of 40% (entry 1, Table 1). This initial low yield prompted us to switch the Grignard solvent from THF to DCM. Using modified reaction conditions from Andersson and co-wokers [22], phenylmagnesium bromide in DCM was added to **2** at −41 °C and after 35 min, dihydropyrazine **3a** was only isolated in a 32% yield (entry 2, Table 1). The lower yields appear to be caused by the Grignard attacking the carbonyl of the *N-*acyl salt **2** causing the formation of phenyl benzoate. When toluene was used as the solvent, both **3a** and the ester were obtained in yields of 41% and 39%, respectively (entry 3, Table 1). Changing the solvent to diethyl ether showed no improvement in the yield of **3a** but when THF was used, an excellent yield of 87% was produced (entries 4 and 5, Table 1).

**Table 1:** Phenyl Grignard addition to methoxy-substituted *N*-acylpyrazinium salts.

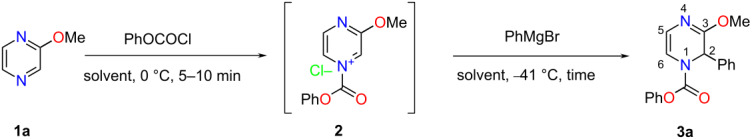			

entry^a^	solvent	time (min)	yield **3a** (%)^b^

1	DCM	60	40
2^c^	DCM	35	32
3	toluene	40	41^d^
4	diethyl ether	60	15
5	THF	30	87^e^

^a^Grignard reagent in THF. ^b^Isolated yields. ^c^Grignard reagent added as solution in DCM. ^d^PhCO_2_Ph (39%) was also isolated. ^e^Trace amounts of the ester byproduct PhCO_2_Ph was isolated.

Based on our previously developed selective tin hydride reduction of monosubstituted pyrazinium salts [9], we expected the Grignard reagent to add regioselectively to give 1,2-dihydropyrazine **3a**. DFT calculations support the observations that the isolated regioisomer we obtained was the result of a thermodynamically favored 1,2-addition over a 1,6-addition [9]. It has also been shown that TMS-ketene acetals add selectively to *N*-triflate pyrazinium salts [12]. ^1^H NMR analysis confirmed this by showing a rotameric pair of singlets at 3.89 and 3.84 ppm for the methoxy group at C3, a rotameric pair of singlets at 5.89 and 5.58 ppm for the proton at C2 and a rotameric pair of doublets at 6.17, 6.15 ppm and 6.68, 6.63 ppm for the two vinyl protons at C5 and C6, respectively. This result is in agreement with our observation that nucleophiles favored 1,2-addition over a 1,6-addition [9]. A formation of 1,6-dihydropyrazine was not observed.

**Figure 1:**
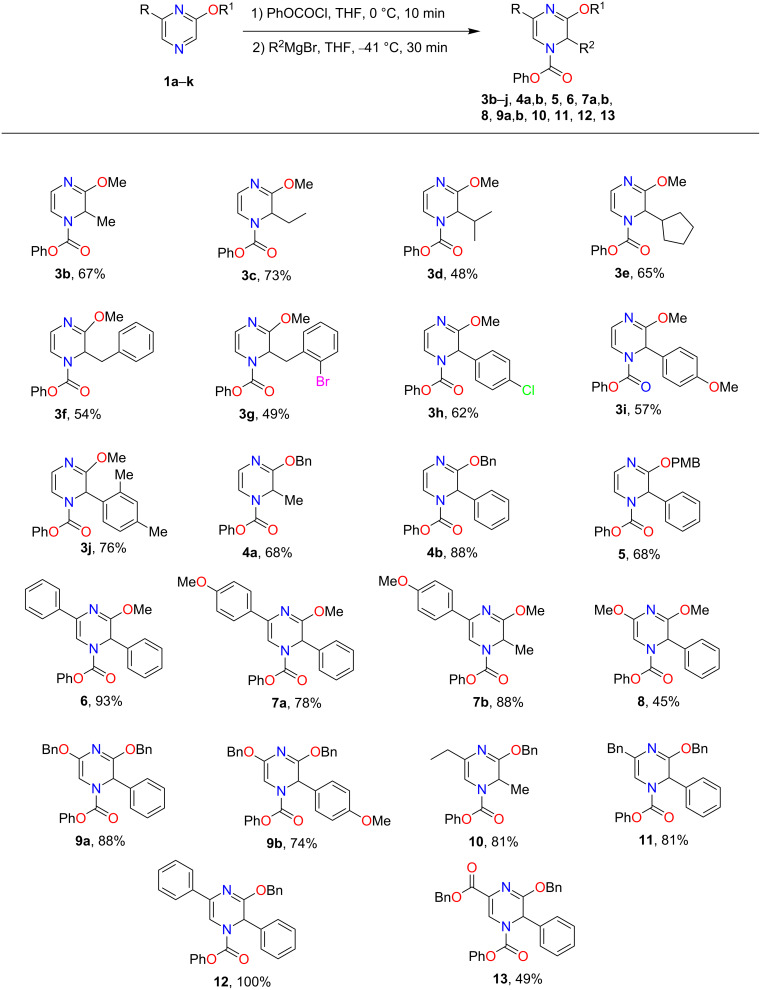
Regioselective addition of Grignard reagents to mono- and disubstituted pyrazinium salts (yields refer to isolated yields).

With THF identified as the optimal solvent to use, we sought to expand the scope of the Grignard addition to various mono- and disubstituted *N*-acylpyrazinium salts (Figure 1). Phenyl chloroformate was the acylating reagent of choice for this study due to benzyl or methyl chloroformates producing products in very poor yields. A variety of alkyl Grignard reagents were shown to add regioselectively to methoxy-substituted salts to give the dihydropyrazines in yields ranging from 48% to 73% (Figure 1, compounds **3b**–**g**). Aryl Grignard reagents containing an electron-withdrawing and donating group proceeded to give the desired substituted products in moderate yields (Figure 1, compounds **3h**–**j**). The examination of the Grignard addition to benzyloxy- or *p-*methoxybenzyloxy (PMB)-substituted pyrazinium salts, resulted in obtaining dihydropyrazines in yields that were comparable to the methoxy salts (Figure 1, compounds **4a**,**b** and **5**).

We next subjected disubstituted *N*-acylpyrazinium salts to this reaction. Based on our results from the monosubstituted substrates, a 1,2-addition of the Grignard was expected to occur. When an aryl group was present on the ring, trisubstituted dihydropyrazines in good yields ranging from 78–100% were produced (Figure 1, compounds **6**, **7a**,**b** and **12**). An alkyl substitution with either an ethyl or benzyl group on the pyrazinium salts gave an 81% yield for both **10** and **11** (Figure 1). Reacting a Grignard with pyrazinium salts disubstituted with electron-donating alkoxy groups gave us the desired dihydropyrazine in moderate to good yields of 45–88% (Figure 1, compounds **8**, **9a**,**b**) while the presence of an electron-withdrawing ester group generated **13** in a yield of 49%.

With the substituted 1,2-dihydropyrazines in hand, we next wanted to demonstrate their usefulness for synthesizing substituted Δ^5^-2-oxopiperazines. Processes into this structural motif would be very useful due to their presence in a variety of biologically active small molecules and natural products [23–30]. We previously reported that 3-methoxy-1,2-dihydropyrazines can be easily converted to Δ^5^-2-oxopiperazines using 1 M HCl_(aq)_ in methanol [9]. When we applied these acidic conditions on the phenyl-substituted 3-methoxy-1,2-dihydropyrazine **3a**, only a 5% yield of Δ^5^-2-oxopiperazine **14a** was obtained (Table 2, entry 1). This low yield appears to be due to the presence of a ring-opened side product [31]. The yields of the Δ^5^-2-oxopiperazines were improved to 61% and 92%, respectively, when the reaction was repeated on phenyl-substituted 1,2-dihydropyrazines containing benzyl and *p*-methoxybenzyl (PMB) ether groups (Table 2, entries 2 and 3). This enhancement in yield can be attributed to the benzyl and PMB groups’ better sensitivity towards removal under acidic conditions [32]. With the aqueous acidic conditions not being suitable for converting 3-methoxy-1,2-dihydropyrazine **3a**, we decided to run this reaction under anhydrous acidic conditions. After stirring dihydropyrazine **3a** in the presence of HCl/dioxane at 0 °C for 30 min, we were pleased to see that Δ^5^-2-oxopiperazine **14a** was obtained in an excellent yield of 93% (Table 2, entry 4). Subjecting both benzyl and 2-bromobenzyl-substituted dihydropyrazines **3f** and **3g** to these acidic conditions gave 95% and 88% yields of **14b** and **14c**, respectively (Table 2, entries 5 and 6). When we examined the use of anhydrous acidic conditions on **4b** and **5**, quantitative yields of Δ^5^-2-oxopiperazine **14a** were obtained (Table 2, entries 7 and 8). Finally, under these conditions, the trisubstituted dihydropyrazine **7a** was easily converted to a disubstituted Δ^5^-2-oxopiperazine **15a** in a yield of 73% (Table 2, entry 9).

**Table 2:** Conversion of dihydropyrazine to Δ^5^-2-oxopiperazines under acidic conditions.

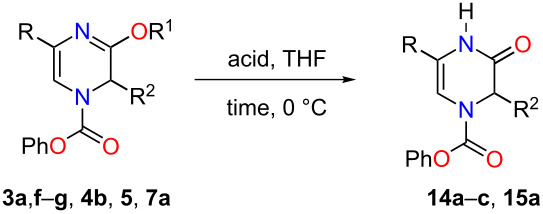							

entry	R	R^1^	R^2^	acid	time (min)	product	yield (%)^a^

1	H	Me	Ph	HCl_(aq)_/MeOH	60	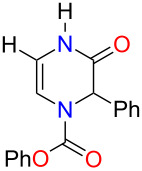 **14a**	5
2	H	Bn	Ph	HCl_(aq)_/MeOH	45	**14a**	61
3	H	PMB	Ph	HCl_(aq)_/MeOH	60	**14a**	92
4^b^	H	Me	Ph	HCl/dioxane	30	**14a**	93
5^b^	H	Me	Bn	HCl/dioxane	30	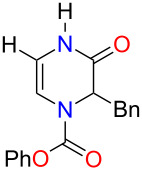 **14b**	95
6^b^	H	Me	2-BrBn	HCl/dioxane	45	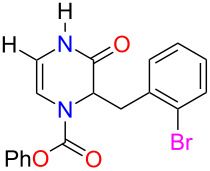 **14c**	85
7^b^	H	Bn	Ph	HCl/dioxane	20	**14a**	100
8^b^	H	PMB	Ph	HCl/dioxane	10	**14a**	100
9^b^	*p-*MeOPh	Me	Ph	HCl/dioxane	45	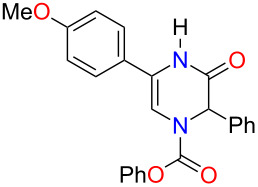 **15a**	73

^a^Isolated yields. ^b^4 M HCl/dioxane was used.

Following the step-wise conversion of 1,2-dihydropyrazines to Δ^5^-2-oxopiperazines, we decided to develop a one-pot approach towards their synthesis (Table 3). As described above, we first synthesized the phenyl-substituted 1,2-dihydropyrazine **4b** by adding a phenyl Grignard reagent to benzyloxy-substituted *N*-acylpyrazinium salt in THF at −41 °C. Next, 1 M HCl_(aq)/_MeOH was added and the reaction was monitored by TLC. After 1 h, the hydrolysis of **4b** was completed to give **14a** in a good yield of 80% (Table 3, entry 1). Disubstituted Δ^5^-2-oxopiperazines **15b**–**d** can be made similarly with yields ranging from 71–80% (Table 3, entries 2–4). This simple approach towards Δ^5^-2-oxopiperazines provides access into compounds that can be reduced into mono- and disubstituted 2-oxopiperazines [33–34]. This structure is a common scaffold found in natural products and biologically active small molecules [23].

**Table 3:** One-pot synthesis of substituted Δ^5^-2-oxopiperazines.

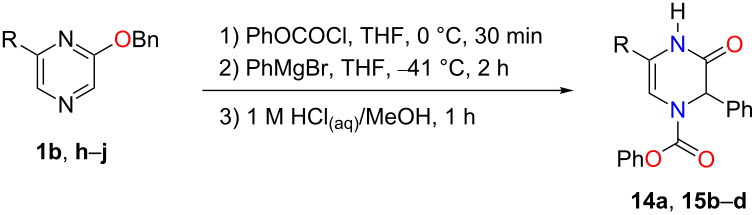			

entry	R	product	yield (%)^a^

1	H	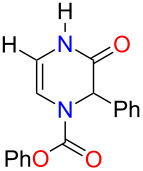 **14a**	80
2	Et	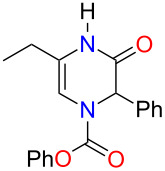 **15b**	80
3	Bn	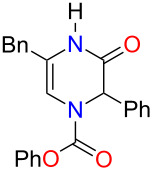 **15c**	72
4	Ph	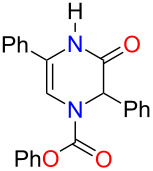 **15d**	71

^a^Isolated yields.

## Conclusion

In conclusion, we have demonstrated that various Grignard reagents can be regioselectively added to mono- and disubstituted *N*-acylpyrazinium salts to give di- and trisubstituted 1,2-dihydropyrazines in moderate to excellent yields. Under acidic conditions, the dihydropyrazines can be easily converted to substituted Δ^5^-2-oxopiperazines. These compounds can potentially serve as templates for making substituted 2-oxopiperazines. The investigation into the synthetic utility of this reaction is currently underway and will be reported in due course.

## Supporting Information

File 1 Experimental section and NMR spectra.
