# Supplementary material for: Regioselective addition of Grignard reagents to N-acylpyrazinium salts: synthesis of substituted 1,2-dihydropyrazines and Δ5-2-oxopiperazines
Source: Beilstein J Org Chem. 2019 Jan 8;15:72–8. doi: 10.3762/bjoc.15.8 (PMC6334805; doi:10.3762/bjoc.15.8)
Supplement: File 1 — Experimental section and NMR spectra. [file Beilstein_J_Org_Chem-15-72-s001.pdf]

BEILSTEIN JOURNAL OF ORGANIC CHEMISTRY

Supporting Information

for

Regioselective addition of Grignard reagents to *N*-acylpyrazinium salts: synthesis of substituted 1,2-dihydropyrazines and Δ^5-2-oxopiperazines

Valentine R. St. Hilaire, William E. Hopkins, Yenteeo S. Miller, Srinivasa R. Dandepally and Alfred L. Williams

Beilstein J. Org. Chem. **2019,** *15,* 72–78. doi:10.3762/bjoc.15.8

Experimental section and NMR spectra

License and Terms: This is a supporting information file under the terms of the Creative Commons Attribution License (http://creativecommons.org/licenses/by/4.0). Please note that the reuse, redistribution and reproduction in particular requires that the authors and source are credited.
The license is subject to the *Beilstein Journal of Organic Chemistry* terms and conditions: (https://www.beilstein-journals.org/bjoc)

Table of Contents .... **Page**

Experimental section S1

Synthesis of mono- and disubsituted pyrazines S1

General procedure for the Grignard addition to 3-substituted *N*-acylpyrazinium salts to synthesize substituted 1,2-dihydropyrazines **3a**–**3j**,**4a**,**b**,**5**,**6**,**7a**,**b**,**8**,**9a**,**b**,**10**,**11**,**12**,**13** S4

General procedure for the conversion of substituted 1,2-dihydropyrazines to Δ^5-2-oxopiperazines using 1 M $HCl_{(aq)}$/methanol S9

General procedure for the conversion of substituted 1,2-dihydropyrazines to Δ^5-2-oxopiperazines using 4 M HCl/dioxane S10

General procedure for the one-pot Grignard addition/hydrolysis reaction to synthesize substituted Δ^5-2-oxopiperazines **15b**–**d** S11

References S12

^{1}H and ^{13}C NMR spectra S13

Experimental section

All solvents and reagents were obtained from commercial sources and were used without further purification unless otherwise stated. Toluene and THF were dried using a solvent purification system. Anhydrous dichloromethane and all acylating agents were pretreated with K_2CO_3 and 4 Å molecular sieves. All reactions were performed in oven-dried glassware (either in round-bottomed flasks or 25 mL vials fitted with rubber septa) under an atmosphere of nitrogen, and the reaction progress was monitored by thin-layer chromatography, GC–MS (EI) and/or LC–MS (ESI-APCI). Analytical thin-layer chromatography was performed on precoated 250 μm layer thickness silica gel 60 F254 plates and precoated 170–220 μm layer thickness neutral aluminum oxide Si 60 F254 plates. Visualization was done by ultraviolet light and/or by staining with phosphomolybdic acid (PMA). Purifications were carried out on flash silica gel columns (230–400 mesh) with EtOAc/hexanes mixtures as the eluent. The eluent was basified with 0.5–1.5% triethylamine for the purification of all Grignard adducts. Melting points were measured on a capillary melting point apparatus and are uncorrected. Proton nuclear magnetic resonance (^{1}H NMR) spectra and carbon nuclear magnetic resonance (^{13}C NMR) spectra were recorded on a 500 MHz spectrometer. Chemical shifts (δ) for protons are reported in parts per million (ppm) downfield from tetramethylsilane and are referenced to it (TMS, δ = 0.0 ppm). Coupling constants (*J*) are reported in hertz. Multiplicities are reported using the following abbreviations: br = broad; s = singlet; d = doublet; t = triplet; q = quartet; m = multiplet. Chemical shifts (δ) for carbon are reported in parts per million (ppm) downfield from tetramethylsilane and are referenced to residual solvent peaks ($CDCl_3$, δ = 77.0 ppm). Rotameric ratios of all compounds were determined by ^{1}H NMR spectra. HRMS data was recorded on a LC-TOF.

Synthesis of mono- and disubsituted pyrazines: Compound **1a** was purchased from a commercial supplier. The known compound **1b** and unknown compound **1c** were prepared by

following the general procedure reported in the literature [1]. The known compounds **1d** [2], **1e** [2] and **1j** [2] are prepared by adopting the procedure reported by Yang and coworkers [3]. Compounds **1f** [4] and **1g** [5**]** were prepared as reported. The known compound **1h** was obtained by a slightly modified procedure [6].

*2-(4-Methoxybenzyloxy)pyrazine (**1c**).* To a suspension of NaH (0.645 g, 26.875 mmol) in anhydrous dimethoxyethane (30 mL) was slowly added dropwise 4-methoxybenzyl alcohol (3.34 mL, 26.907 mmol) at 0 °C over a period of 5 min and stirring continued for 15 min. Then, 2-chloropyrazine (2 mL, 22.405 mmol) was added at 0 °C and the mixture heated at 45 °C overnight. The reaction mixture was quenched with slow addition of water (10 mL) and extracted with EtOAc (100 mL), washed with brine, dried over Na_2SO_4, concentrated under reduced pressure and the resulted crude syrup was purified by flash silica gel column chromatography (Combiflash Rf) using EtOAc–hexanes (1:9) to obtain **1c** (4.650 g, 99%) as a white solid: mp: 69−71 °C; ^{1}H NMR ($CDCl_3$, 500 MHz) δ 8.25 (s, 1H), 8.11 (d, 1H, J = 3.0 Hz), 8.08–8.10 (m, 1H), 7.39 (d, 2H, J = 8.5 Hz), 6.91 (d, 2H, J = 8.5 Hz), 5.32 (s, 2H), 3.81 (s, 3H); ^{13}C NMR ($CDCl_3$, 125 MHz) δ 160.1, 159.6, 140.4, 136.6, 136.1, 130.0, 128.3, 113.9, 67.7, 55.2; HRMS (ESI) *m*/*z* calcd for $C_{12}H_{12}N_2O_2$ $[M+H]^+$ 217.09772, found 217.09715.

*2-Methoxy-6-phenylpyrazine (**1d**).* Starting with 2,6-dichloropyrazine, intermediate 2-chloro-6-methoxypyrazine was prepared as a colorless crystalline solid in 86% yield by adopting the procedure as described above: mp: 26−27 °C; ^{1}H NMR ($CDCl_3$, 300 MHz) δ 8.14 (s, 1H), 8.13 (s, 1 H), 3.99 (s, 3H). ^{13}C NMR ($CDCl_3$, 125 MHz) δ 159.6, 145.5, 135.1, 133.1, 54.6; MS (ESI) *m*/*z* calcd for $C_5H_6ClN_2O$ $[M+H]^+$ 144.01, found 145.10. The compound **1d** was prepared from 2-chloro-6-methoxypyrazine, phenylboronic acid, $Pd(OAc)_2$ and K_3PO_4•H_2O in 61% yield as a white solid: mp: 46−48 °C; ^{1}H NMR ($CDCl_3$, 500 MHz) δ 8.60 (s, 1H), 8.16 (s, 1H), 8.04 (d, J = 7.5 Hz, 2H), 7.56–7.40 (m, 3H), 4.07 (s, 3H); ^{13}C NMR ($CDCl_3$, 125MHz) δ 159.8, 148.9, 136.3, 133.7, 133.0, 129.7, 128.9, 126.8, 53.4; APCI/ESI-MS *m*/*z* calcd for $C_{11}H_{11}N_2O$ $[M+H]^+$ 186.08, found 187.10.

*2-Methoxy-6-(4-methoxyphenyl)pyrazine (**1e**).* The compound **1e** was prepared from 2-chloro-6-methoxypyrazine, 4-methoxyphenylboronic acid, $Pd(OAc)_2$ and $K_3PO_4 \cdot H_2O$ in 65% yield as a white solid: mp: 92−93 °C; ^{1}H NMR ($CDCl_3$, 500 MHz) δ 8.54 (s, 1H), 8.10 (s, 1H), 7.99 (d, J = 8.0 Hz, 2H), 7.01 (d, J = 8.0 Hz, 2H), 4.05 (s, 3H), 3.87 (s, 3H); ^{13}C NMR ($CDCl_3$, 125 MHz) δ 160.9, 159.6, 148.5, 132.5, 132.2, 128.7, 128.0, 114.2, 55.2, 53.2; HRMS (ESI) *m*/*z* calcd for $C_{12}H_{13}N_2O_2$ $[M+H]^+$ 217.09715, found 217.09715.

*2-(Benzyloxy)-6-ethylpyrazine (**1h**).* To a suspension of NaH (0.081 g, 3.375 mmol) in anhydrous dimethoxyethane (10 mL) was slowly added dropwise benzyl alcohol (0.35 mL, 3.379 mmol) at 0 °C and stirring continued for 15 min. Then, 2,6-dichloropyrazine (0.500 g, 3.356 mmol) was added at 0 °C and the mixture heated at 45 °C for 8 h. The reaction mixture was quenched with slow addition of water (5 mL) and extracted with EtOAc (50 mL), washed with brine, dried over Na_2SO_4, concentrated under reduced pressure and the resulted crude syrup was purified by flash silica gel column chromatography (Combiflash Rf) using EtOAc–hexanes (1:19) to obtain 2-(benzyloxy)-6-chloropyrazine (0.560 g, 76%) as a colorless liquid: ^{1}H NMR ($CDCl_3$, 500 MHz) δ 8.17 (d, J = 5.0 Hz, 1H), 7.48–7.45 (m, 2H), 7.42–7.34 (m, 3H), 5.39 (s, 2H); ^{13}C NMR ($CDCl_3$, 125 MHz) δ 159.0, 145.3, 142.4, 135.5, 135.4, 133.3, 128.6, 128.4, 68.8; HRMS (ESI)

m/z calcd for $C_{11}H_{10}ClN_2O$ $[M+H]^+$ 221.0476, found 221.0484. Next, to a solution of 2-(benzyloxy)-6-chloropyrazine (1.000 g, 4.532 mmol) in anhydrous toluene (20 mL) were added first 1,3-bis(diphenylphosphino)propane nickel(II) chloride (0.050 g, 0.092 mmol) and then diethyl zinc (1.5 M in toluene, 3.4 mL, 5.510 mmol) at 0 °C slowly (dropwise), and stirred at room temperature for 2 h. The reaction mixture was quenched with aq. NH_4OH solution (5 mL), extracted with EtOAc (50 mL), washed with brine, dried over Na_2SO_4 and concentrated under reduced pressure. The resulting crude material was purified by flash silica gel column chromatography (Combiflash Rf, 0−10% EtOAc/hexanes) to obtain **1h** (0.920 g, 95%) as a colorless liquid: ^1H NMR ($CDCl_3$, 500 MHz) δ 8.08 (s, 1H), 8.00 (s, 1H), 7.48–7.45 (m, 2H), 7.39–7.35 (m, 2H), 7.34–7.30 (m, 1H), 5.40 (s, 2H), 2.74 (q, 2H, J = 7.5 Hz), 1.30 (dt, 3H, J = 1.0, 7.5 Hz); ^{13}C NMR ($CDCl_3$, 125 MHz) δ 159.3, 155.0, 136.7, 135.0, 132.6, 128.4, 128.0, 128.2, 67.5, 28.0, 13.1; HRMS (ESI) m/z calcd for $C_{13}H_{15}N_2O$ $[M+H]^+$ 215.11789, found 215.11782.

2-Benzyl-6-benzyloxypyrazine (1i). To a solution of 2-(benzyloxy)-6-chloropyrazine (0.200 g, 0.906 mmol) in anhydrous THF (4 mL) were added first 1,3-bis(diphenylphosphino)propane nickel(II) chloride (0.010 g, 0.018 mmol) and then benzylmagnesium chloride solution (2M in THF, 0.5 mL, 0.994 mmol) at 0 °C slowly (dropwise), and stirred at room temperature for 2 h. The reaction mixture was quenched with aq. NH_4Cl solution (4 mL), extracted with EtOAc (20 mL), washed with brine, dried over Na_2SO_4 and concentrated under reduced pressure. The resulted crude material was purified by flash silica gel column chromatography (Combiflash Rf, 0−10% EtOAc/hexanes) to afford **1i** (0.130 g, 52%) as a colorless syrup: ^1H NMR ($CDCl_3$, 500 MHz) δ 8.09 (s, 1H), 8.01 (s, 1H), 7.42–7.39 (m, 2H), 7.37–7.22 (m, 8H), 5.37 (s, 2H), 4.04 (s, 2H); ^{13}C NMR ($CDCl_3$, 125 MHz) δ 159.3, 152.4, 138.4 (2), 136.6, 135.6, 133.1, 129.2, 129.1, 128.6, 128.5, 128.3, 128.0, 126.6, 67.7, 41.4; HRMS (ESI) m/z calcd for $C_{18}H_{17}N_2O$ $[M+H]^+$ 277.13354, found 277.13347.

2-Benzyloxy-6-phenylpyrazine (1j). To a solution of 2-(benzyloxy)-6-chloropyrazine (0.600 g, 2.719 mmol) in 2-propanol (5 mL) were added phenylboronic acid (0.500 g, 4.101 mmol), $Pd(OAc)_2$ (0.030 g, 0.134 mmol, 5 mol %), $K_3PO_4{\cdot}H_2O$ (1.200 g, 5.211 mmol) and water (5 mL), and the mixture heated at 80 °C for 12 h. The reaction mixture was diluted with EtOAc (30 mL), washed with brine, dried over Na_2SO_4, concentrated under reduced pressure and the resulted crude syrup was purified by flash silica gel column chromatography (Combiflash Rf, 0−10% EtOAc/hexanes) to afford **1j** (0.700 g, 98%) as a white solid: mp: 58−60 °C; ^1H NMR ($CDCl_3$, 500 MHz) δ 8.61 (s, 1H), 8.21 (s, 1H), 8.02 (d, J = 8.0 Hz, 2H), 7.52–7.43 (m, 5H), 7.40 (t, J = 7.0 Hz, 2H), 7.36–7.32 (m, 1H), 5.51 (s, 2H); ^{13}C NMR ($CDCl_3$, 125 MHz) δ 159.2, 148.8, 136.6, 136.2, 133.8, 133.3, 129.7, 128.9, 128.5, 128.2, 128.1, 126.8, 67.8; HRMS (ESI) m/z calcd for $C_{17}H_{15}N_2O$ $[M+H]^+$ 263.1179, found 263.1180.

*Benzyl 6-(benzyloxy)pyrazine-2-carboxylate (**1k**).* A stirred mixture of benzyl 4-hydroxypyrazine-2-carboxylate (2.000 g, 8.69 mmol) in 10 mL of thionyl chloride was heated at 80 °C for 10 h. The volatiles were removed under reduced pressure, then the residue was

quenched with saturated $NaHCO_3$, extracted with CH_2Cl_2 (3 × 30 mL), dried over Na_2SO_4, concentrated under reduced pressure and then purified by flash silica gel column chromatography (Combiflash Rf, 0−10% EtOAc/hexanes) to afford benzyl 6-chloropyrazine-2-carboxylate (1.700 g, 79%) as a white solid: mp: 64−65 °C; ^{1}H NMR ($CDCl_3$, 500 MHz) δ 9.19 (s, 1H), 8.76 (s, 1H), 7.52−7.45 (m, 2H), 7.44−7.33 (m, 3H), 5.47 (s, 2H); ^{13}C NMR ($CDCl_3$, 125 MHz) δ 162.4, 149.1, 147.9, 143.5, 142.5, 134.7, 128.6, 128.5(2), 67.9; HRMS (ESI) *m*/*z* calcd for $C_{12}H_9ClNaN_2O_2$ [M+Na]+ 271.02448, found 271.02494. Next, to a suspension of sodium hydride (0.106 g, 2.65 mmol) in anhydrous dimethoxyethane (5 mL) was slowly added (dropwise) benzyl alcohol (0.274 mL 2.65 mmol) at 0 °C and stirring continued for 15 min. Then, benzyl 6-chloropyrazine-2-carboxylate (0.500 g, 2.01 mmol) was added at 0 °C and the mixture was heated at 45 °C for 12 h. The volatiles were removed under reduced pressure and the residue purified by flash silica gel column chromatography (Combiflash Rf, 0−50% EtOAc/hexanes) to afford **1k** (0.354 g, 55%) as clear colorless syrup: ^{1}H NMR ($CDCl_3$, 500 MHz) δ 8.86 (s, 1H), 8.41 (s, 1H), 7.54−7.45 (m, 4H), 7.45−7.28 (m, 6H), 5.47 (s, 2H), 5.44 (s, 2H); ^{13}C NMR ($CDCl_3$, 125 MHz) δ 164.0, 159.3, 139.8, 139.4, 138.2, 135.8, 135.4, 128.8, 128.7, 128.6, 128.4, 68.6, 67.5; HRMS (ESI) *m*/*z* calcd for $C_{19}H_{17}N_2O_3$ [M+H]+ 321.12337, found 321.12332.

General procedure for the Grignard addition to 3-substituted N-acylpyrazinium salts to synthesize substituted 1,2-dihydropyrazines 3a–3j, 4a,b, 5, 6, 7a,b, 8, 9a,b, 10, 11, 12, 13. *Representative procedure for the preparation of 1-phenoxycarbonyl-2-phenyl-3-methoxy-1,2-dihydropyrazine (**3a**).* To a stirred solution of 2-methoxypyrazine (0.200 g, 1.816 mmol) in anhydrous THF (5 mL) was added phenylchloroformate (0.28 mL, 2.225 mmol) at 0 °C and stirring was continued under nitrogen atmosphere until salt formation was completed (15 min, as determined by TLC on neutral alumina, EtOAc/hexanes 1:19). The reaction mixture was cooled to −41 °C, a 1 M solution of phenylmagnesium bromide in THF (2.4 mL, 2.4 mmol) was added and the mixture stirred until reaction completion (40 min, as determined by TLC on neutral SiO_2, EtOAc/hexanes 1:9), and then quenched with 2 mL of aqueous 20% NH_4OH/NH_4Cl 1:1 (w/w). The mixture was extracted with dichloromethane (2 × 15 mL), dried over Na_2SO_4, and concentrated in vacuo. Purification of the crude mixture by flash silica gel column chromatography (0–15% EtOAc/hexanes) afforded **3a** (0.488 g, 87%) as a white solid (3:2 mixture of rotamers): mp: 81−82 °C; ^{1}H NMR ($CDCl_3$, 500 MHz) δ 7.49−7.27 (m, 7H), 7.20 (t, *J* = 7.5 Hz, 1H), 7.12 (d, *J* = 8.0 Hz, 1H), 6.99 (d, *J* = 7.5 Hz, 1H), 6.67 and 6.63 (2d due to rotamers, *J* = 5.5 Hz, 1H), 6.16 and 6.14 (2d due to rotamers, *J* = 5.0 Hz, 1H), 5.89 and 5.87 (2s due to rotamers, 1H), 3.84 and 3.83 (2s due to rotamers, 3H); ^{13}C NMR ($CDCl_3$, 125 MHz) δ 160.2, 160.0, 151.6, 151.5, 150.7, 150.4, 136.8, 135.9, 129.3, 128.8, 128.7, 128.6, 127.2, 126.6, 125.9, 125.8, 121.3, 118.2, 118.1, 112.5, 111.8, 56.0, 54.6, 54.0, 53.9; HRMS (ESI) *m*/*z* calcd for $C_{18}H_{17}N_2O_3$ $[M + H]^+$ 309.12337, found 309.123554.

*1-Phenoxycarbonyl-2-methyl-3-methoxy-1,2-dihydropyrazine (**3b**).* Compound **3b** was prepared in 67% yield as a yellowish syrup (3:2 mixture of rotamers): ^{1}H NMR ($CDCl_3$, 500 MHz) δ 7.43−7.34 (m, 2H), 7.28−7.19 (m, 1H), 7.14 (dd, *J* = 1.0, 8.5 Hz, 2H), 6.50 and 6.46 (2d due to rotamers, *J* = 5.5 Hz, 1H), 6.13 and 6.07 (2d due to rotamers, *J* = 5.5 Hz, 1H), 4.91 and 4.86 (2m due to rotamers, 1H), 3.83 and 3.82 (2s due to rotamers, 3H), 1.35 and 1.28 (2d due to rotamers,

J = 7.0 Hz, 3H); ^{13}C NMR ($CDCl_3$, 125 MHz) *δ* 162.3, 161.9, 151.2, 150.9, 150.7, 150.5, 129.4, 129.3 (2), 125.8, 125.7, 121.4, 121.3, 121.1, 118.4, 118.1, 111.1, 110.7, 53.7, 53.6, 48.6, 47.7, 15.5, 14.6; HRMS (ESI) *m*/*z* calcd for $C_{13}H_{15}N_2O_3$ $[M + H]^+$ 247.10772, found 247.10770.

1-Phenoxycarbonyl-2-ethyl-3-methoxy-1,2-dihydropyrazine (3c). Compound **3c** was prepared in 73% yield as a white solid (3:2 mixture of rotamers): mp: 80−82 °C; ^{1}H NMR ($CDCl_3$, 500 MHz) *δ* 7.45−7.33 (m, 2H), 7.28−7.19 (m, 1H), 7.14 (d, *J* = 8.0 Hz, 2H), 6.53 and 6.49 (2d due to rotamers, *J* = 5.0 Hz, 1H), 6.13. and 6.07 (2d due to rotamers, *J* = 5.5 Hz, 1H), 4.81 and 4.76 (2t due to rotamers, *J* = 7.0 Hz, 1H), 3.83 and 3.82 (2s due to rotamers, 3H), 1.81−1.61 (m, 2H), 1.01 and 0.96 (2t due to rotamers, *J* = 7.0 Hz, 1H); ^{13}C NMR ($CDCl_3$, 125 MHz) *δ* 161.9, 161.4, 151.8, 151.7, 150.9, 150.6, 129.5, 129.4, 125.9, 125.8, 121.5, 121.4, 119.2, 118.7, 111.9, 111.4, 54.0, 53.7, 53.6, 53.0, 23.7, 23.3, 9.7; HRMS (ESI) *m*/*z* calcd for $C_{14}H_{16}NaN_2O_3$ $[M + Na]^+$ 283.10531, found 283.10509.

1-Phenoxycarbonyl-2-isopropyl-3-methoxy-1,2-dihydropyrazine (3d). Compound **3d** was prepared in 48% yield as an off-white solid (3:2 mixture of rotamers): mp: 45−47 °C; ^{1}H NMR ($CDCl_3$, 500 MHz) *δ* 7.43−7.35 (m, 2H), 7.30−7.18 (m, 1H), 7.16−7.10 (m, 2H), 6.55 and 6.52 (2dd due to rotamers, *J* = 1.5, 5.0 Hz, 1H), 6.17 and 6.09 (2d due to rotamers, *J* = 5.0 Hz, 1H), 3.84 and 3.82 (2s due to rotamers, 3H), 2.15−2.00 (m, 1H), 1.05−0.99 (m, 1H), 0.97 (t, *J* = 7.0 Hz, 6H); ^{13}C NMR ($CDCl_3$, 125 MHz) *δ* 161.1, 160.6, 152.2, 152.1, 151.0, 150.9, 150.6, 129.6, 129.5, 129.4, 126.3, 125.9, 125.8, 121.5, 121.4, 120.9, 120.0, 119.5, 112.2, 111.8, 58.2, 57.1, 53.6, 53.5, 30.0, 29.9, 19.4, 19.3, 18.6; HRMS (ESI) *m*/*z* calcd for $C_{15}H_{19}N_2O_3$ $[M + H]^+$ 275.13902, found 275.13901.

1-Phenoxycarbonyl-2-cyclopentyl-3-methoxy-1,2-dihydropyrazine (3e). Compound **3e** was prepared in 65% yield as a white solid (3:2 mixture of rotamers): mp: 78−80 °C; ^{1}H NMR ($CDCl_3$, 500 MHz) *δ* 7.45−7.34 (m, 2H), 7.30−7.19 (m, 1H), 7.13 (d, *J* = 8.5 Hz, 2H), 6.53 and 6.50 (2d due to rotamers, *J* = 5.0 Hz, 1H), 6.19 and 6.12 (2d due to rotamers, *J* = 5.0 Hz, 1H), 4.70 and 4.67 (2dd due to rotamers, *J* = 1.5, 9.0 Hz, 1H), 3.84 and 3.83 (2s due to rotamers, 3H), 2.37−2.19 (m, 1H), 1.82−1.60 (m, 4H), 1.60−1.41(m, 4H); ^{13}C NMR ($CDCl_3$, 125 MHz) *δ* 161.7, 161.2, 152.1, 152.0, 151.0, 150.9, 150.6, 129.6, 129.5, 129.4, 126.3, 125.9, 125.7, 121.5, 121.4, 120.9, 120.0, 119.5, 112.0, 111.5, 56.2, 55.0, 53.7, 53.6, 40.9, 40.8, 29.6, 29.5, 28.6 (2), 24.9, 24.7, 24.3, 24.2; HRMS (ESI) *m*/*z* calcd for $C_{17}H_{21}N_2O_3$ $[M + H]^+$ 301.15467, found 301.15502.

1-Phenoxycarbonyl-2-benzyl-3-methoxy-1,2-dihydropyrazine (3f). Compound **3f** was prepared in 54% yield as a colorless syrup (1:1 mixture of rotamers): ^{1}H NMR ($CDCl_3$, 500 MHz) *δ* 7.41−7.08 (m, 8H), 6.96 (d, *J* = 7.5 Hz, 1H), 6.58 (d, *J* = 7.5 Hz, 1H), 6.55 and 6.22 (2d due to rotamers, *J* = 5.5 Hz, 1H), 6.49 and 6.04 (2d due to rotamers, *J* = 5.5 Hz, 1H), 5.11−4.99 (m, 1H), 3.82 and 3.74 (2s due to rotamers, 3H), 3.02−2.80 (m, 2H); ^{13}C NMR ($CDCl_3$, 125 MHz) *δ* 161.0, 160.8, 151.5, 151.2, 150.7, 150.1, 135.8, 135.7, 129.5, 129.2, 129.0, 128.5, 128.2, 126.9, 126.7, 125.6, 121.3, 121.2, 119.6, 118.6, 111.3, 111.1, 53.9, 53.8, 53.5, 52.8, 35.3. 35.1; HRMS (ESI) *m*/*z* calcd for $C_{19}H_{19}N_2O_3$ $[M + H]^+$ 323.13902, found 323.13932.

1-Phenoxycarbonyl-2-(2-bromobenzyl)-3-methoxy-1,2-dihydropyrazine (3g). Compound **3g** was prepared in 49% yield as a white solid (1:1 mixture of rotamers): mp: 124−126 °C; ^{1}H NMR

(CDCl$_3$, 500 MHz) δ 7.52 (t, *J* = 9.0 Hz, 1H), 7.30 (t, *J* = 8.0 Hz, 1H), 7.21−7.08 (m, 3H), 6.80 (d, *J* = 8.0 Hz, 1H), 6.67 (d, *J* = 8.0 Hz, 1H), 6.55 (t, *J* = 4.0 Hz, 1H), 6.26 and 6.09 (2d due to rotamers, *J* = 5.5 Hz, 1H), 5.34−5.27 (m) and 5.21 (t, *J* = 6.5 Hz), (total 1H), 3.82 and 3.77 (2s due to rotamers, 3H), 3.21 and 3.18 (2d, *J* = 5.5 Hz, 1H), 3.14 and 3.11 (2d, *J* = 5.0 Hz, 1H), 3.07−2.96 (m, 2H); ^{13}C NMR (CDCl$_3$, 125 MHz) δ 160.7, 160.6, 151.4, 151.3, 150.7, 150.2, 135.5, 135.3, 132.9, 131.9, 131.6, 129.3, 129.1, 128.8, 128.6, 127.6, 127.2, 125.8, 125.6, 125.3, 125.2, 121.4, 121.1, 119.7, 118.7, 111.5, 111.3, 53.9, 53.8, 51.8, 51.3, 35.2, 35.1; HRMS (ESI) *m*/*z* calcd for $C_{19}H_{18}BrN_2O_3$ [M + H]$^+$ 401.04953, found 401.05022.

*1-Phenoxycarbonyl-2-(4-chlorophenyl)-3-methoxy-1,2-dihydropyrazine (**3h**).* Compound **3h** was prepared in 62% yield as a colorless syrup (3:2 mixture of rotamers): ^{1}H NMR (CDCl$_3$, 500 MHz) δ 7.46−7.28 (m, 6H), 7.28−7.18 (m, 1H), 7.12 (d, *J* = 8.5 Hz, 1H), 7.00 (d, *J* = 8.5 Hz, 1H), 6.67 and 6.63 (2d due to rotamers, *J* = 5.5 Hz, 1H), 6.16 and 6.14 (2d due to rotamers, *J* = 5.5 Hz, 1H), 5.84 (s, 1H), 3.85 and 3.84 (2s due to rotamers, 3H); ^{13}C NMR (CDCl$_3$, 125 MHz) δ 159.8, 159.7, 151.6, 151.5, 150.7, 150.4, 135.5, 134.7, 134.6, 129.5, 129.1, 129.0, 128.8, 128.2, 126.1, 126.0, 121.4, 118.2, 118.1, 112.5, 111.8, 55.5, 54.2, 54.1; HRMS (ESI) *m*/*z* calcd for $C_{18}H_{16}ClN_2O_3$ [M + H]$^+$ 343.08440, found 343.08453.

*1-Phenoxycarbonyl-2-(4-methoxyphenyl)-3-methoxy-1,2-dihydropyrazine (**3i**).* Compound **3i** was prepared in 74% yield as a white solid (3:2 mixture of rotamers): mp: 102−104 °C; ^{1}H NMR (CDCl$_3$, 500 MHz) δ 7.40−7.29 (m, 4H), 7.22 (t, *J* = 7.5 Hz, 1H), 7.12 (d, *J* = 8.5 Hz, 1H), 7.03 (d, *J* = 8.0 Hz, 1H), 6.87 (t, *J* = 10.5 Hz, 2H), 6.64 and 6.61 (2d due to rotamers, *J* = 5.5 Hz, 1H), 6.17 and 6.14 (2d due to rotamers, *J* = 5.5 Hz, 1H), 5.83 and 5.82 (2s due to rotamers, 1H), 3.85 and 3.84 (2s due to rotamers, 3H), 3.81 and 3.79 (2s due to rotamers, 3H); ^{13}C NMR (CDCl$_3$, 125 MHz) δ 160.6, 160.5, 159.9, 151.7, 151.6, 150.8, 150.6, 129.5, 129.4, 129.1, 128.8, 128.3, 128.2, 126.0, 125.9, 121.5, 118.3, 118.2, 114.2, 112.5, 111.8, 55.5, 55.3, 54.2, 54.1, 54.0; HRMS (ESI) *m*/*z* calcd for $C_{19}H_{19}N_2O_4$ [M + H]$^+$ 339.13393, found 339.13412.

*1-Phenoxycarbonyl-2-(2,4-dimethylphenyl)-3-methoxy-1,2-dihydropyrazine (**3j**).* Compound **3j** was prepared in 76% yield as a colorless syrup (3:2 mixture of rotamers): ^{1}H NMR (CDCl$_3$, 500 MHz) δ 7.44 and 7.37 (2d due to rotamers, 1H), 7.34−7.21 (m, 2H), 7.21−6.91 (m, 4H), 6.84−6.72 (m), 6.56 (br s) and 6.32 (br s), (total 2H), 6.10 (dd, *J* = 1.0, 5.5 Hz, 1H), 5.99−5.92 (m, 1H), 3.76−3.69 (m, 3H), 2.52 (s), 2.46−2.41 (m) and 2.31−2.25 (m) (total 6H); ^{13}C NMR (CDCl$_3$, 125 MHz) δ 161.7, 161.4, 151.4, 151.3, 150.6, 150.3, 138.2, 138.0, 135.9, 135.6, 134.9, 134.3, 131.1, 130.8, 129.2, 129.1, 129.0, 127.6 (2), 127.2, 126.4, 126.3, 125.6 (2), 121.4, 121.1, 117.1, 116.6, 113.7, 113.2, 112.6, 53.7, 52.5, 51.5, 20.9, 20.2, 19.3, 19.2; HRMS (ESI) *m*/*z* calcd for $C_{20}H_{21}N_2O_3$ [M + H]$^+$ 337.15467, found 337.15505.

*1-Phenoxycarbonyl-2-methyl-3-benzyloxy-1,2-dihydropyrazine (**4a**).* Compound **4a** was prepared in 68% yield as a white solid (3:2 mixture of rotamers): mp: 60−62 °C; ^{1}H NMR (CDCl$_3$, 500 MHz) δ 7.45−7.32 (m, 7H), 7.29−7.21 (m, 1H), 7.17−7.11 (m, 2H), 6.53 and 6.48 (2d due to rotamers, *J* = 5.5 Hz, 1H), 6.15 and 6.09 (2d due to rotamers, *J* = 5.5 Hz, 1H), 5.24 and 5.23 (2s due to rotamers, 2H), 4.98 and 4.92 (2q due to rotamers, *J* = 7.0 Hz, 1H), 1.38 and 1.31 (2d due to rotamers, *J* = 7.0 Hz, 3H); ^{13}C NMR (CDCl$_3$, 125 MHz) δ 161.6, 161.2, 151.3, 151.0, 150.7, 150.5, 136.0, 135.9, 129.4 (2), 128.5 (2), 128.2 (2), 128.1, 128.0, 125.8 (2), 121.5, 121.4, 118.4,

118.1, 111.4, 110.9, 68.3, 68.1, 48.8, 47.9, 15.6, 14.7; HRMS (ESI) *m*/*z* calcd for $C_{19}H_{19}N_2O_3$ $[M + H]^+$ 323.13902, found 323.13861.

*1-Phenoxycarbonyl-2-phenyl-3-benzyloxy-1,2-dihydropyrazine (**4b**).* Compound **4b** was prepared in 88% yield as a white solid (3:2 mixture of rotamers): mp: 95−96 °C; ^{1}H NMR ($CDCl_3$, 500 MHz) δ 7.46−7.26 (m, 12H), 7.26−7.18 (m, 1H), 7.17−7.10 (m, 1H), 6.99 (d, *J* = 8.0 Hz, 1H), 6.70 and 6.65 (2d due to rotamers, *J* = 5.5 Hz, 1H), 6.18 and 6.15 (2d due to rotamers, *J* = 5.5 Hz, 1H), 5.95 and 5.94 (2s due to rotamers, 1H), 5.38−5.18 (m, 2H); ^{13}C NMR ($CDCl_3$, 125 MHz) δ 159.4, 159.3, 151.8, 151.7, 150.8, 150.5, 136.9, 136.0, 135.9, 129.4, 128.9 (2), 128.7, 128.5 (2), 128.2 (2), 128.1, 128.0, 127.3, 126.7, 126.0, 125.9, 121.5 (2), 118.4, 118.2, 112.8, 112.1, 68.6, 68.4, 56.1, 54.8; HRMS (ESI) *m*/*z* calcd for $C_{24}H_{21}N_2O_3$ $[M + H]^+$ 385.15467, found 385.15394.

*1-Phenoxycarbonyl-2-phenyl-3-(4-methoxybenzyloxy)-1,2-dihydropyrazine (**5**).* Compound **5** was prepared in 68% yield as a white solid (3:2 mixture of rotamers): mp: 90−91 °C; ^{1}H NMR ($CDCl_3$, 500 MHz) δ 7.44−7.29 (m, 7H), 7.29−7.17 (m, 3H), 7.13 (d, *J* = 8.5 Hz, 1H), 6.90−6.82 (m, 2H), 6.69 and 6.63 (2d due to rotamers, *J* = 5.5 Hz, 1H), 6.18 and 6.15 (2d due to rotamers, *J* = 5.5Hz, 1H), 5.92 and 5.91 (2s due to rotamers, 1H), 5.30−5.11 (m, 2H), 3.79 (2s due to rotamers, 3H); ^{13}C NMR ($CDCl_3$, 125 MHz) δ 159.6, 159.5, 159.4, 159.3, 151.7, 151.6, 150.7, 150.4, 136.7, 135.9, 129.9, 129.8, 129.3 (2), 128.7 (2), 128.6 (2), 127.9 (2), 127.2, 126.6, 125.9, 125.8, 121.4 (2), 118.4, 118.2, 113.8 (2), 112.6, 111.9, 68.3, 68.2, 56.0, 55.2, 54.7; HRMS (ESI) *m*/*z* calcd for $C_{25}H_{23}N_2O_4$ $[M + H]^+$ 415.16523, found 415.16468.

*1-Phenoxycarbonyl-2,5-diphenyl-3-methoxy-1,2-dihydropyrazine (**6**).* Compound **6** was prepared in 93% yield as an yellow solid (3:2 mixture of rotamers): mp: 118−120 °C; ^{1}H NMR ($CDCl_3$, 500 MHz) δ 7.85−7.76 (m, 2H), 7.46−7.27 (m, 9H), 7.27−7.19 (m, 2H), 7.19−7.11 (m, 2H), 7.03 (d, *J* = 7.5 Hz, 1H), 5.95 and 5.93 (2s due to rotamers, 1H), 3.99 and 3.98 (2s due to rotamers, 3H); ^{13}C NMR ($CDCl_3$, 125 MHz) δ 159.4, 151.8, 150.7, 150.5, 136.7, 136.4, 136.3, 135.8, 129.3 (2), 128.8 (2), 128.6, 128.4, 128.3, 127.3 (2), 127.2, 126.7, 125.9, 125.8, 124.5, 124.4, 121.4, 121.3, 108.1, 107.3, 55.5, 54.3, 54.0, 53.9; HRMS (ESI) *m*/*z* calcd for $C_{24}H_{21}N_2O_3$ $[M + H]^+$ 385.15467, found 385.15462.

*1-Phenoxycarbonyl-2-phenyl-3-methoxy-5-(4-methoxyphenyl)-1,2-dihydropyrazine (**7a**).* Compound **7a** was prepared in 78% yield as a yellow solid (3:2 mixture of rotamers): mp: 48−50 °C; ^{1}H NMR ($CDCl_3$, 500 MHz) δ 7.80−7.69 (m, 2H), 7.42−7.28 (m, 8H), 7.26−7.20 (m, 1H), 7.18−7.11 (m, 1H), 7.05 and 7.03 (2s due to rotamers, 1H), 6.96−6.89 (m, 2H), 5.94 and 5.92 (2s due to rotamers, 1H), 3.98 and 3.97 (2s due to rotamers, 3H), 3.83 (2s due to rotamers, 3H); ^{13}C NMR ($CDCl_3$, 125 MHz) δ 159.6, 159.5, 159.3, 159.2, 152.0, 151.9, 150.9, 150.7, 136.8, 135.9, 129.5, 129.4, 129.2, 128.9 (2), 128.7(3), 128.4, 127.5, 126.9, 126.0, 125.9 (2), 125.8, 121.6, 121.5, 114.0, 113.9, 106.6, 105.9, 55.6, 55.4, 54.4, 54.2, 54.0; HRMS (ESI) *m*/*z* calcd for $C_{25}H_{23}N_2O_4$ $[M+H]^+$ 415.16523, found 415.16442.

*1-Phenoxycarbonyl-2-methyl-3-methoxy-5-(4-methoxyphenyl)-1,2-dihydropyrazine (**7b**).* Compound **7b** was prepared in 88% yield as a colorless syrup (3:2 mixture of rotamers): ^{1}H NMR ($CDCl_3$, 500 MHz) δ 7.77−7.68 (m, 2H), 7.44−7.36 (m, 2H), 7.29−7.21 (m, 1H), 7.21−7.15 (m, 2H), 6.96−6.89 (m, 3H), 4.96 and 4.91 (2q due to rotamers, *J* = 6.5 Hz, *J* = 7.0

Hz, 1H), 3.96 and 3.95 (2s due to rotamers, 3H), 3.83 and 3.82 (2s due to rotamers, 3H), 1.37 and 1.30 (2d due to rotamers, J = 6.5 Hz, 3H); ^{13}C NMR ($CDCl_3$, 125 MHz) δ 161.7, 161.4, 159.2, 159.1, 151.6, 151.4, 150.9, 150.7, 129.5, 129.4, 129.3 (2), 128.5, 128.3, 125.9, 125.8, 125.7 (2), 121.5, 121.4, 113.9, 113.8, 105.4, 104.9, 55.3, 53.8, 53.7, 48.3, 47.4, 16.1, 15.2; HRMS (ESI) *m/z* calcd for $C_{20}H_{21}N_2O_4$ [M+H]$^+$ 353.14958, found 353.14900.

*1-Phenoxycarbonyl-2-phenyl-3,5-dimethoxy-1,2-dihydropyrazine (**8**).* Compound **8** was prepared in 45% yield as a colorless syrup (3:2 mixture of rotamers): ^{1}H NMR ($CDCl_3$, 500 MHz) δ 7.43−7.30 (m, 6H), 7.27−7.18 (m, 1H), 7.14 (d, J = 7.5 Hz, 1H), 7.04 (d, J = 7.5 Hz, 1H), 5.98 and 5.89 (2s due to rotamers 1H), 5.93 and 5.92 (2s due to rotamers 1H), 6.01−5.87 (m, 2H), 3.91 (2s due to rotamers, 3H), 3.69 (2s due to rotamers, 3H); ^{13}C NMR ($CDCl_3$, 125 MHz) δ 161.6, 161.4, 152.2, 151.5, 150.8, 150.6, 148.0, 147.8, 135.7, 135.0, 129.3 (2), 128.8, 128.7 (3), 127.1, 126.6, 125.7 (2), 121.5, 121.4, 87.5, 87.1, 55.6, 55.1 (2), 54.6, 54.5, 54.4; HRMS (ESI) *m/z* calcd for $C_{19}H_{18}NaN_2O_4$ [M+Na]$^+$ 361.11588, found 361.11560.

*1-Phenoxycarbonyl-2-phenyl-3,5-dibenzyloxy-1,2-dihydropyrazine (**9a**).* Compound **9a** was prepared in 88% yield as a colorless syrup (3:2 mixture of rotamers): ^{1}H NMR ($CDCl_3$, 500 MHz) δ 7.39−7.24 (m, 17H), 7.22−7.15 (m, 1H), 7.13−7.09 (m) and 7.04−6.99 (m) (total 2H), 6.14 and 6.10 (2s due to rotamers, 1H), 5.94 and 5.91 (2s due to rotamers, 1H), 5.41−5.27 (m, 2H), 5.02−4.90 (m, 2H); ^{13}C NMR ($CDCl_3$, 125 MHz) δ 160.5, 160.3, 152.1, 151.5, 150.9, 150.6, 145.9, 145.6, 136.5, 136.4, 135.8, 135.5, 135.4, 135.2, 129.3 (2), 128.8, 128.7, 128.6 (2), 128.5, 128.4 (2), 128.3, 128.2, 128.1, 128.0, 127.9, 127.1, 126.6, 125.7, 125.6, 121.5, 121.4, 91.2, 90.9, 69.8, 69.7, 69.2, 69.0, 55.7, 54.6; HRMS (ESI) *m/z* calcd for $C_{31}H_{27}N_2O_4$ [M+H]$^+$ 491.19653, found 491.19661.

*1-Phenoxycarbonyl-2-(4-methoxyphenyl)-3,5-dibenzyloxy-1,2-dihydropyrazine (**9b**).* Compound **9b** was prepared in 77% yield as a colorless syrup (3:2 mixture of rotamers): ^{1}H NMR ($CDCl_3$, 500 MHz) δ 7.38−7.29 (m, 11H), 7.24−6.96 (m, 6H), 6.83 and 6.78 (2d due to rotamers, J = 8.0 Hz), 6.10 and 6.07 (2s due to rotamers, 1H), 5.88 and 5.85 (2s due to rotamers, 1H), 5.40−5.27 (m, 2H), 5.04−4.93 (m, 2H), 3.81 and 3.78 (2s due to rotamers, 3H); ^{13}C NMR ($CDCl_3$, 125 MHz) δ 166.8, 160.7, 159.8, 152.1, 151.5, 151.0, 150.8, 146.0, 145.7, 136.6, 136.5, 135.6 (2), 132.2, 129.4 (2), 128.7, 128.6, 128.5 (2), 128.4, 128.3, 128.2, 128.1 (3), 127.4, 125.8, 125.7, 121.6, 121.5, 114.2, 114.1, 113.5, 91.0 , 90.8, 69.8, 69.7, 69.2, 69.0, 55.3(2), 55.2, 54.1; HRMS (ESI) *m/z* calcd for $C_{32}H_{29}N_2O_5$ [M+H]$^+$ 521.20710, found 521.20718.

*1-Phenoxycarbonyl-2-methyl-3-(benzyloxy)-5-ethyl-1,2-dihydropyrazine (**10**).* Compound **10** was prepared in 81% yield as a colorless syrup (3:2 mixture of rotamers): ^{1}H NMR ($CDCl_3$, 500 MHz) δ 7.46−7.30 (m, 7H), 7.30−7.19 (m, 1H), 7.17−7.10 (m, 2H), 6.29 and 6.24 (2s due to rotamers, 1H), 5.26 and 5.25 (2s due to rotamers, 2H), 4.91 and 4.86 (2q due to rotamers, J = 6.5 Hz, 1H), 2.33−2.21 (m, 2H), (2d due to rotamers, J = 7.0 Hz, 3H), 1.20−1.10 (m, 3H); ^{13}C NMR ($CDCl_3$, 125 MHz) δ 161.3, 160.9, 151.4, 151.3, 151.0 (2), 150.8, 136.3, 132.5, 132.2, 129.6, 129.4 (2), 128.5 (2), 128.4, 128.2 (2), 128.1, 126.3, 125.7 (2), 121.6, 121.5, 120.9, 105.2, 104.7, 68.1, 67.9, 48.3, 47.5, 26.9, 15.7, 14.8, 12.4 (2); HRMS (ESI) *m/z* calcd for $C_{21}H_{23}N_2O_3$ [M + H]$^+$ 351.17032, found 351.17015.

*1-Phenoxycarbonyl-2-phenyl-3-benzyloxy-5-benzyl-1,2-dihydropyrazine (**11**).* Compound **11** was prepared in 81% yield as a colorless syrup (3:2 mixture of rotamers): ^{1}H NMR ($CDCl_3$, 500 MHz) δ 7.44−7.08 (m) and 7.00 (d, J = 8.0 Hz) (total 20H), 6.52 and 6.46 (2s due to rotamers, 1H), 5.89 and 5.86 (2s due to rotamers, 1H), 5.33−5.15 (m, 2H), 3.65−3.49 (m, 2H); ^{13}C NMR ($CDCl_3$, 125 MHz) δ 158.9, 151.8, 150.8, 150.6, 139.0, 136.0, 135.5, 130.4, 130.2, 129.4, 129.3, 129.1 (2), 128.7, 128.6, 128.5 (2), 128.4, 128.3 (2), 128.2 (2), 128.0 (2), 127.4, 126.8, 126.2 (2), 125.8, 125.7, 121.5, 121.4, 108.2, 107.4, 68.3, 68.2, 55.4, 54.0, 40.0 (2); HRMS (ESI) *m*/*z* calcd for $C_{31}H_{27}N_2O_3$ $[M+H]^+$ 475.20162, found 475.20297.

*1-Phenoxycarbonyl-2,5-diphenyl-3-benzyloxy-1,2-dihydropyrazine (**12**).* Compound **12** was prepared in 100% yield as an yellow syrup (3:2 mixture of rotamers): ^{1}H NMR ($CDCl_3$, 500 MHz) δ 7.80 (d, J = 7.5 Hz, 2H), 7.47−7.20 (m, 16H**)**, 7.20−7.13 (m, 2H), 7.03 (d, J = 8.5 Hz, 1H), 6.00 and 5.99 (2s due to rotamers, 1H), 5.53−5.37 (m, 2H); ^{13}C NMR ($CDCl_3$, 125 MHz) δ 158.7, 151.9, 150.8, 150.6, 136.6, 136.4 (2), 136.1, 135.8, 129.2 (2), 128.9, 128.8, 128.7 (2), 128.5, 128.4 (2), 128.2 (2), 128.1, 127.2 (2), 127.3, 126.8, 126.0, 125.9, 124.6, 124.5, 121.5, 121.4, 108.4, 107.6, 68.6, 68.5, 55.7, 54.4; HRMS (ESI) *m*/*z* calcd for $C_{30}H_{25}N_2O_3$ $[M+H]^+$ 461.18597, found 461.18689.

*1-Phenoxycarbonyl-2-phenyl-3-benzyloxy-5-(benzyloxycarbonyl)-1,2-dihydropyrazine (**13**).* Compound **13** was prepared in 49% yield as a white solid (3:2 mixture of rotamers): ^{1}H NMR ($CDCl_3$, 500 MHz) δ 7.97 and 7.90 (2br s due to rotamers, 1H), 7.48−7.43 (m, 2H), 7.41−7.22 (m, 16H), 7.14−7.09 (m, 1H), 6.96−6.92 (m, 1H), 5.90 (s, 1H), 5.45−5.31 (m, 4H); ^{13}C NMR ($CDCl_3$, 125 MHz) δ 164.6, 158.7, 158.6, 151.4, 151.1, 150.4, 150.2, 136.6, 136.1, 135.6, 129.5, 129.0, 128.5 (2), 128.4, 128.3, 128.2, 127.8, 127.3, 126.7, 126.3, 122.1, 121.3, 121.2, 120.2, 69.0, 66.5, 56.3, 55.0; HRMS (ESI) *m*/*z* calcd for $C_{32}H_{27}N_2O_5$ $[M+H]^+$ 519.19145, found 519.19150.

General procedure for the conversion of substituted 1,2-dihydropyrazines to Δ^5-2-oxopiperazines using 1 M $HCl_{(aq)}$/methanol. *Representative procedure for the preparation of phenyl 3-oxo-2-phenyl-3,4-dihydropyrazine-1(2H)-carboxylate (**14a**).* To a solution of phenyl 3-(benzyloxy)-2-phenylpyrazine-1(2*H*)-carboxylate (**4b**, 0.100 g, 0.260 mmol) in anhydrous THF (1 mL) was added 1M $HCl_{(aq)}$ in methanol (1 mL) at 0 °C. Stirring was continued under nitrogen for 45 minutes until hydrolysis was completed as determined by TLC (silica gel, EtOAc/hexanes 2:3). The reaction mixture was quenched with saturated $NaHCO_3$ (2 mL), extracted with dichloromethane (3 × 10 mL), dried over Na_2SO_4 and concentrated in vacuo. Purification of the crude mixture by flash silica gel column chromatography (10–75% EtOAc/hexanes) afforded **15a** as a white solid (0.058 g, 61%, 1:1 mixture of rotamers): mp: 164–166 °C; ^{1}H NMR ($CDCl_3$, 500 MHz) δ 8.28 and 8.02 (2br s due to rotamers, 1H), 7.58−7.43 (m, 2H), 7.43−7.29 (m, 5H), 7.29−7.18 (m, 1H), 7.14 (d, J = 8.5 Hz, 1H), 6.98 (d, J = 8.5 Hz, 1H), 5.96 and 5.93 (2s due to rotamers, 1H), 5.83−5.68 (m, 1H); ^{13}C NMR ($CDCl_3$, 125 MHz) δ 166.1, 165.8, 151.8, 151.4, 150.6, 150.4, 136.5, 135.9, 129.4, 128.9, 128.8, 128.7, 126.9, 126.4, 126.0, 125.9, 121.3, 108.9, 108.7, 108.2, 60.9, 59.8; HRMS (ESI) *m*/*z* calcd for $C_{17}H_{14}NaN_2O_3$ $[M + Na]^+$ 317.08966, found 317.08968.

*Ring-opened side product methyl 2-((2-oxoethyl)(phenoxycarbonyl)amino)-2-phenylacetate (**16**).* ^{1}H NMR ($CDCl_3$, 500 MHz, 4:1 mixture of rotamers) δ 3.81 and 3.84 (2s due to rotamers, 3H),

3.91−4.17 (m, 2H), 6.22 and 6.27 (2s due to rotamers, 1H), 7.12−7.26 (m, 3H), 7.28−7.32 (m, 2H), 7.35−7.44 (m, 5H), 9.40 and 9.49 (2s due to rotamers, 1H); ^{13}C NMR ($CDCl_3$, 125 MHz, mixture of rotamers) δ 52.5, 52.6, 53.8, 54.0, 62.0, 62.7, 121.4, 125.8 (2), 128.8 (2), 129.3 (2), 133.3, 133.6, 150.7, 150.8, 154.4, 154.8, 170.8, 171.0, 197.7, 197.9; HRMS (ESI) *m*/*z* calcd for $C_{18}H_{18}NO_5$ $[M+H]^+$ 328.11795, found 328.11758.

General procedure for the conversion of substituted 1,2-dihydropyrazines to Δ^5-2-oxopiperazines using 4 M HCl/dioxane. *Representative procedure for the preparation of phenyl 3-oxo-2-phenyl-3,4-dihydropyrazine-1(2H)-carboxylate (**14a**).* To a solution of of phenyl 3-(benzyloxy)-2-phenylpyrazine-1(2*H*)-carboxylate (**4b**, 0.100 g, 0.260 mmol) in anhydrous THF (1 mL) was added 4 M HCl in dioxane (1 mL) at 0 °C. Stirring was continued under nitrogen for 20 minutes until hydrolysis was completed as determined by TLC (silica gel, EtOAc/hexanes 2:3). The reaction mixture was quenched with water (2 mL) and extracted with dichloromethane (3 × 15 mL). The organic phase was washed with saturated $NaHCO_3$ (25 mL), dried over Na_2SO_4 and concentrated in vacuo. Purification of the crude mixture by flash silica gel column chromatography (10–75% EtOAc/hexanes) afforded **14a** as a white solid (0.259 g, 100%).

*Phenyl 2-benzyl-3-oxo-3,4-dihydropyrazine-1(2H)-carboxylate (**14b**).* Compound **14b** was prepared in 95% yield as a white crystalline solid (3:2 mixture of rotamers): mp: 128−130 °C; ^{1}H NMR ($CDCl_3$, 500 MHz) δ 8.44 and 8.39 (2s due to rotamers, 1H), 7.43−7.10 (m, 8H), 6.93 (d, *J* = 8.0 Hz, 1H), 6.55 (d, *J* = 8.0 Hz, 1H), 6.55 (d, *J* = 8.0 Hz, 1H), 6.55 (d, *J* = 8.0 Hz, 1H), 6.44 and 6.37 (2d due to rotamers, *J* = 5.5 Hz, 1H), 5.20−5.05 (m, 1H), 5.80 and 5.60 (2d due to rotamers, *J* = 4.5 Hz, 1H), 3.21−2.97 (m, 2H); ^{13}C NMR ($CDCl_3$, 125 MHz) δ 167.0, 166.9, 151.7, 151.2, 150.6, 150.1, 135.7, 135.5, 129.7, 129.3, 129.1, 128.7, 128.4, 127.1, 127.0, 125.8, 125.7, 121.3, 121.2, 110.4, 109.1, 108.0, 107.8, 59.1, 57.8, 36.0, 35.9; HRMS (ESI) *m*/*z* calcd for $C_{18}H_{16}NaN_2O_3$ $[M+Na]^+$ 331.10531, found 331.10499.

*Phenyl 2-(2-bromobenzyl)-3-oxo-3,4-dihydropyrazine-1(2H)-carboxylate (**14c**).* Compound **14c** was prepared in 85% yield as a white solid (1:1 mixture of rotamers): mp: 40−42 °C; ^{1}H NMR ($CDCl_3$, 500 MHz) δ 8.34 and 8.30 (2br s due to rotamers, 1H), 7.54 (t, *J* = 8.5 Hz, 2H), 7.35−7.10 (m) and 6.93 (d, *J* = 7.5 Hz) (total 5H), 6.62 (d, *J* = 7.5 Hz, 1H), 6.48−6.40 (m, 1H), 5.86 and 5.65 (2t due to rotamers, *J* = 5.0 Hz, 1H), 5.38−5.32 (m) and 5.29−5.24 (m) (total 1H), 3.79−3.74 (m) and 3.68−3.61 (m) (total 1H), 3.42 and 3.39 (dt, *J* = 5.0 Hz), 3.35 and 3.34 (dd, *J* = 4.0 Hz, (total 1H), 3.17−3.08 (m, 1H); ^{13}C NMR ($CDCl_3$, 125 MHz) δ 166.6, 166.4, 151.5, 151.2, 150.6, 150.1, 135.3, 135.2, 133.0,132.9, 131.8, 131.7, 129.4, 129.3, 129.1, 128.9, 128.8, 127.6, 127.2, 125.8, 125.7, 125.4,125.3, 121.4, 121.3, 121.0, 110.4, 109.2, 108.1, 108.0, 72.2, 71.1, 61.6, 57.1, 56.5, 42.8, 36.0, 35.7; HRMS (ESI) *m*/*z* calcd for $C_{18}H_{16}BrN_2O_3$ $[M + H]^+$ 387.03388, found 387.03360.

*Phenyl 5-(4-methoxyphenyl)-3-oxo-2-phenyl-3,4-dihydropyrazine-1(2H)-carboxylate (**15a**).* Compound **15a** was prepared in 73% yield as a white solid (1:1 mixture of rotamers): mp: 206–208 °C; ^{1}H NMR ($CDCl_3$, 500 MHz) δ 7.87 and 7.70 (2br s due to rotamers, 1H), 7.48 (t, *J* = 6.0 Hz, 2H), 7.41−7.30 (m, 7H), 7.26−7.21 (m, 1H), 7.17 (d, *J* = 8.0 Hz, 1H), 7.01 (d, *J* = 8.0 Hz, 1H), 6.93 (dd, *J* = 4.0, 8.5 Hz, 1H), 6.84 and 6.77 (2s due to rotamers, 1H), 6.02 (d, *J* =11.5 Hz, 1H), 3.82 (s, 3H); ^{13}C NMR ($CDCl_3$, 125 MHz) δ 166.0, 165.8, 160.1 (2), 152.0, 151.8, 150.8, 150.6, 136.3, 135.8, 129.5, 129.3, 129.2, 129.0, 128.9, 128.8, 128.7, 126.9, 126.4, 126.1, 126.0

(2), 124.6, 122.1 (2), 121.5, 121.4, 114.5, 104.7, 104.2, 60.6, 59.5, 55.4; HRMS (ESI) *m*/*z* calcd for $C_{24}H_{21}N_2O_4$ $[M]^+$ 400.14958, found 400.14878.

General procedure for the one-pot Grignard addition/hydrolysis reaction to synthesize substituted Δ^5-2-oxopiperazines 15b–d. *Representative procedure for the preparation of phenyl 5-ethyl-3-oxo-2-phenyl-3,4-dihydropyrazine-1(2H)-carboxylate (**15b**).* To a solution of 2-(benzyloxy)-6-ethylpyrazine (0.100 g, 0.467 mmol) in anhydrous THF (4 mL) was added phenyl chloroformate (0.07 mL, 0.556 mmol) at 0 °C and stirred under a nitrogen atmosphere until salt formation was completed (15 min, as determined by TLC on neutral alumina, EtOAc/hexanes 1:19). The reaction mixture was cooled to −41 °C, phenylmagnesium bromide solution (1 M in THF, 0.56 mL, 0.556 mmol) was added and the mixture stirred for 2 h. Then, the reaction was quenched with 1 M HCl solution in MeOH (2 mL) at −41 °C, allowed to reach room temperature and stirred for 1 h. The reaction mixture was neutralized with aqueous $NaHCO_3$ solution, extracted with EtOAc (20 mL), washed with brine, dried over Na_2SO_4 and concentrated under reduced pressure. The resulted crude material was purified by flash silica gel column chromatography (Combiflash Rf) using EtOAc/hexanes 1:3 to obtain **15b** (0.120 g, 80%) as a colorless syrup (1:1 mixture of rotamers): ^1H NMR ($CDCl_3$, 500 MHz) δ 7.58 and 7.82 (2br s due to rotamers, 1H, NH), 7.42 (t, *J* = 7.5 Hz, 2H), 7.32–7.40 (m, 5H), 7.18–7.24 (m, 1H), 7.14 (d, *J* = 8.0 Hz, 1H), 7.00 (d, *J* = 8.5 Hz, 1H), 6.31 and 6.35 (2s due to rotamers, 1H), 5.94 and 5.96 (2s due to rotamers, 1H), 2.18–2.26 (m, 2H), 1.12–1.18 (m, 3H). ^{13}C NMR ($CDCl_3$, 125 MHz) δ 166.6, 166.5, 151.8, 151.7, 150.7, 150.5, 136.3, 135.8, 129.3, 128.8, 128.7, 128.5 (2), 126.8, 126.3, 125.8, 125.7, 123.9, 123.8, 121.4, 121.3, 103.4, 103.0, 60.2, 59.0, 23.3, 11.5, 11.4; HRMS (ESI) *m*/*z* calcd for $C_{19}H_{19}N_2O_3$ $[M+H]^+$ 323.13902, found 323.13895.

*Phenyl 5-benzyl-3-oxo-2-phenyl-3,4-dihydropyrazine-1(2H)-carboxylate (**15c**).* Compound **15c** was prepared in 72% yield as an orange solid (1:1 mixture of rotamers): mp: 88–90 ^oC; ^1H NMR ($CDCl_3$, 500 MHz) δ 8.49 and 8.14 (2br s due to rotamers, 1H), 7.40–7.13 (m, 14H), 7.01 (d, *J* = 8.0 Hz, 1H), 6.50 and 6.47 (2s due to rotamers, 1H), 5.90 (d, 1H, *J* = 4.5 Hz), 3.48–3.54 (m, 2H); ^{13}C NMR ($CDCl_3$, 125 MHz) δ 165.9, 165.6, 151.8, 151.6, 150.7, 150.5, 136.0, 135.8, 135.7, 135.5, 129.4, 128.8, 128.7 (3), 128.6, 128.5, 127.2, 126.9, 126.3, 125.9, 125.8, 121.4 (2), 121.2, 105.5, 104.9, 60.3, 59.1, 36.4 (2); HRMS (ESI) *m*/*z* calcd for $C_{19}H_{18}N_2NaO_3$ $[M+Na]^+$ 407.13661, found 407.13640.

*Phenyl 3-oxo-2,5-diphenyl-3,4-dihydropyrazine-1(2H)-carboxylate (**15d**).* Compound **15d** was prepared in 71% yield as a white solid (1:1 mixture of rotamers): mp: 208−210 °C; ^1H NMR ($CDCl_3$, 500 MHz) δ 7.63−7.34 (m, 13H), 7.25−7.21 (m, 1H), 7.17 and 7.01 (2d due to rotamers, *J* = 8.0 Hz, 2H), 6.97 and 6.90 (2s due to rotamers, 1H), 6.03 (d, *J* = 12.5 Hz, 1H); ^{13}C NMR ($CDCl_3$, 125 MHz) δ 59.7, 60.8, 105.5, 106.0, 121.4, 121.5, 121.8 (2), 124.4, 124.5, 126.0, 126.1, 126.3, 126.8, 128.8, 129.0 (2), 129.3, 129.5, 132.2, 135.8, 136.2, 148.7, 150.5, 150.7, 151.7, 152.0, 165.2, 165.3; HRMS (ESI) *m*/*z* calcd for $C_{23}H_{18}N_2NaO_3$ $[M+Na]^+$ 393.1215, obsd 393.1206.

References

1 (a) Masuda, H.; Mihara, S. *Agric. Biol. Chem.* **1989**, *53*, 3367−3368. (b) Lanni, E. L.; Bosscher, M. A.; Ooms, B. D.; Shandro, C. A.; Ellsworth, B. A.; Anderson, C. E. *J. Org. Chem.* **2008**, *73*, 6425–6428.

2 Smith, C. L.; Hirschhaeuser, C.; Malcolm, G. K.; Nasrallah, D. J.; Gallagher, T. *Synlett* **2014**, *25*, 1904−1908.

3 Liu, C.; Yang, W. *Chem. Commun.*, **2009**, 6267–6269

4 2,6-Dimethoxypyrazine (**1f**) and 2,6-bis(benzyloxy)pyrazine (**1g**) were synthesis from 1,6-dichloropyrazine. Guillou, S.; Jacob, G.; Terrier, F.; Goumont, R. *Tetrahedron* **2009**, *65*, 8891−8895.

5 Niedermann, H.-P.; Munro, D. Brit. UK Pat. Appl., 2285045, 28 Jun 1995.

6 Motohashi, K.; Inaba, K.; Fuse, S.; Doi, T.; Izumikawa, M.; Khan, S. T.; Takagi, M.; Takahshi, T.; Shin-ya, K. *J. Nat. Prod.* **2011**, *74*, 1630−1635.

2-(4-Methoxybenzyloxy)pyrazine (1c)

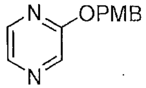

Sample: DBR-4-593
Sample ID: s_20120319_04
File: s_20120319_04/data/cdcl3_

Pulse Sequence: s2pul
Solvent: cdcl3
Temp. 26.0 C / 299.1 K
Operator: walkup
File: cdcl3_01
VNMRS-500 "NMR500"

Relax. delay 1.000 sec
Pulse 45.0 degrees
Acq. time 2.049 sec
Width 8012.8 Hz
8 repetitions
OBSERVE H1, 499.7316307 MHz
DATA PROCESSING
Resol. enhancement -0.0 Hz
FT size 65536
Total time 0 min, 31 sec

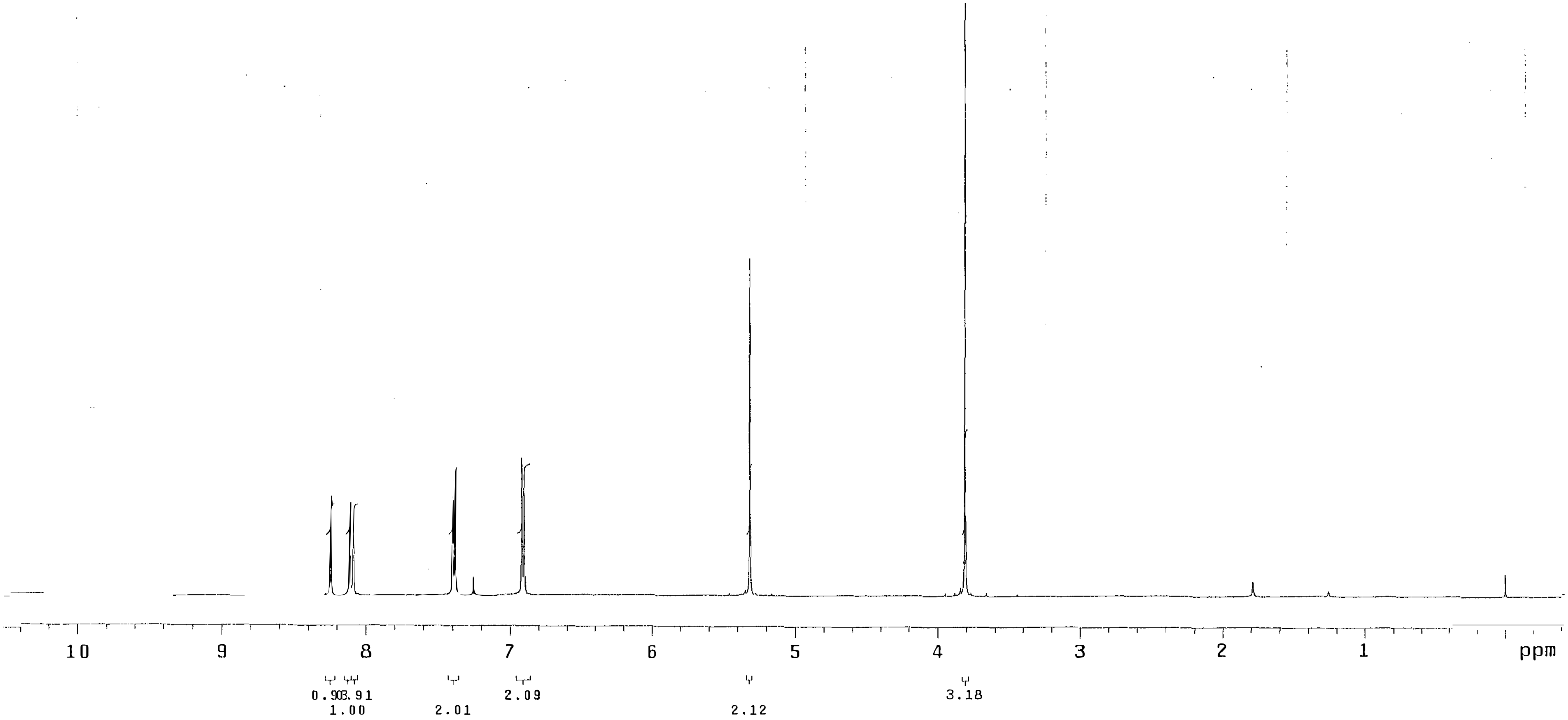

2-(4-Methoxybenzyloxy)pyrazine (1c)

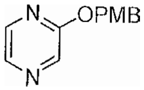

Sample: DBR-4-593
Sample ID: s_20120319_05
File: s_20120319_05/data/cdcl3_

Pulse Sequence: s2pul

Solvent: cdcl3
Temp. 26.0 C / 299.1 K
Operator: walkup
File: cdcl3_01
VNMRS-500 "NMR500"

Relax. delay 1.000 sec
Pulse 45.0 degrees
Acq. time 1.300 sec
Width 30487.8 Hz
256 repetitions
OBSERVE C13, 125.6576224 MHz
DECOUPLE H1, 499.7341220 MHz
Power 42 dB
continuously on
WALTZ-16 modulated
DATA PROCESSING
Line broadening 0.5 Hz
FT size 131072
Total time 9 min, 51 sec

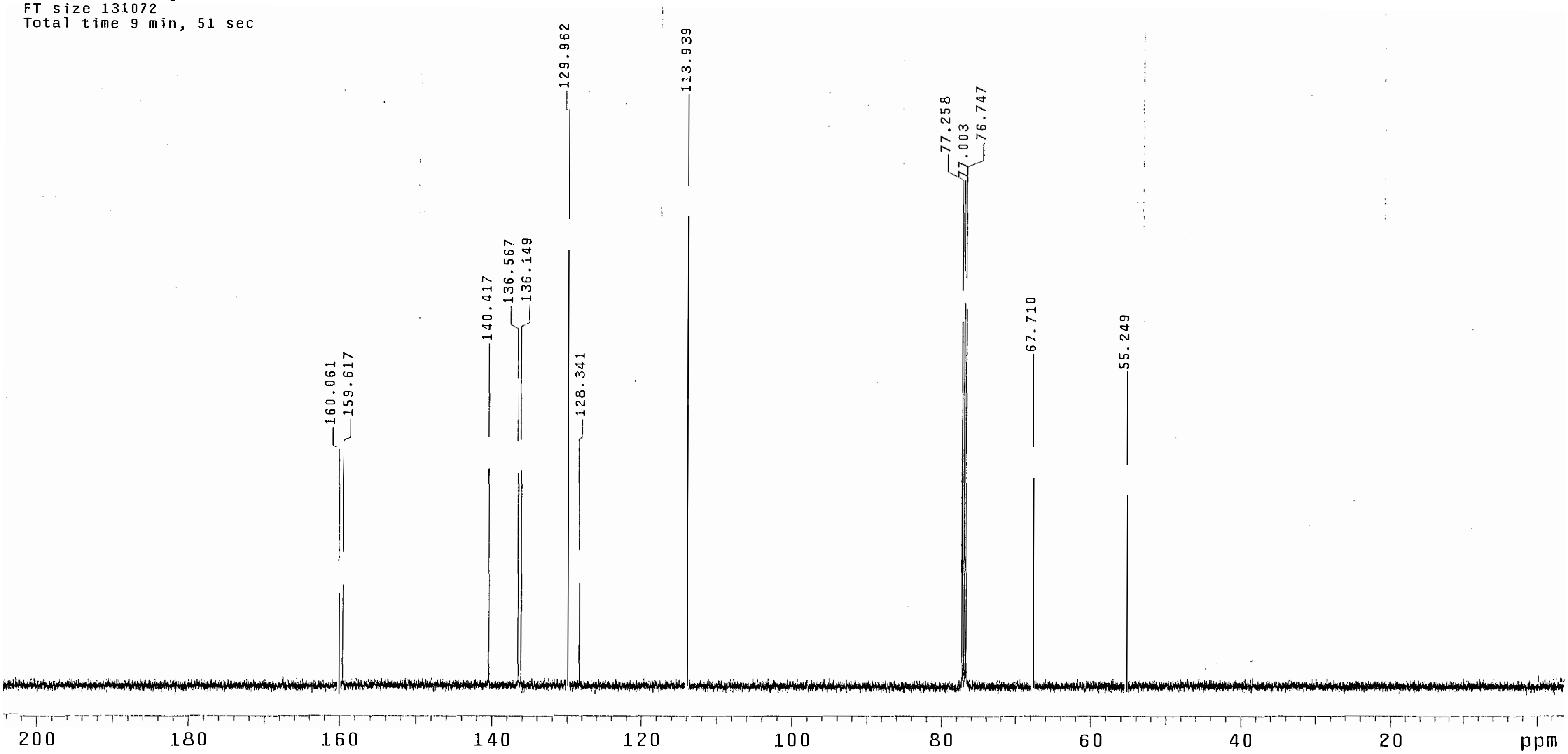

Band 1

Sample: VRS-6-111
Sample ID: s_20120926_07
File: s_20120926_07/data/cdcl3_01.fid

Pulse Sequence: s2pul

Solvent: cdcl3
Temp. 26.0 C / 299.1 K
Operator: walkup
File: cdcl3_01
VNMRS-500 "NMR500"

Relax. delay 1.000 sec
Pulse 45.0 degrees
Acq. time 2.049 sec
Width 8012.8 Hz
8 repetitions
OBSERVE H1, 499.7316167 MHz
DATA PROCESSING
Resol. enhancement -0.0 Hz
FT size 65536
Total time 0 min, 31 sec

2-Chloro-6-methoxypyrazine

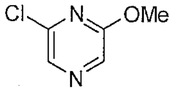

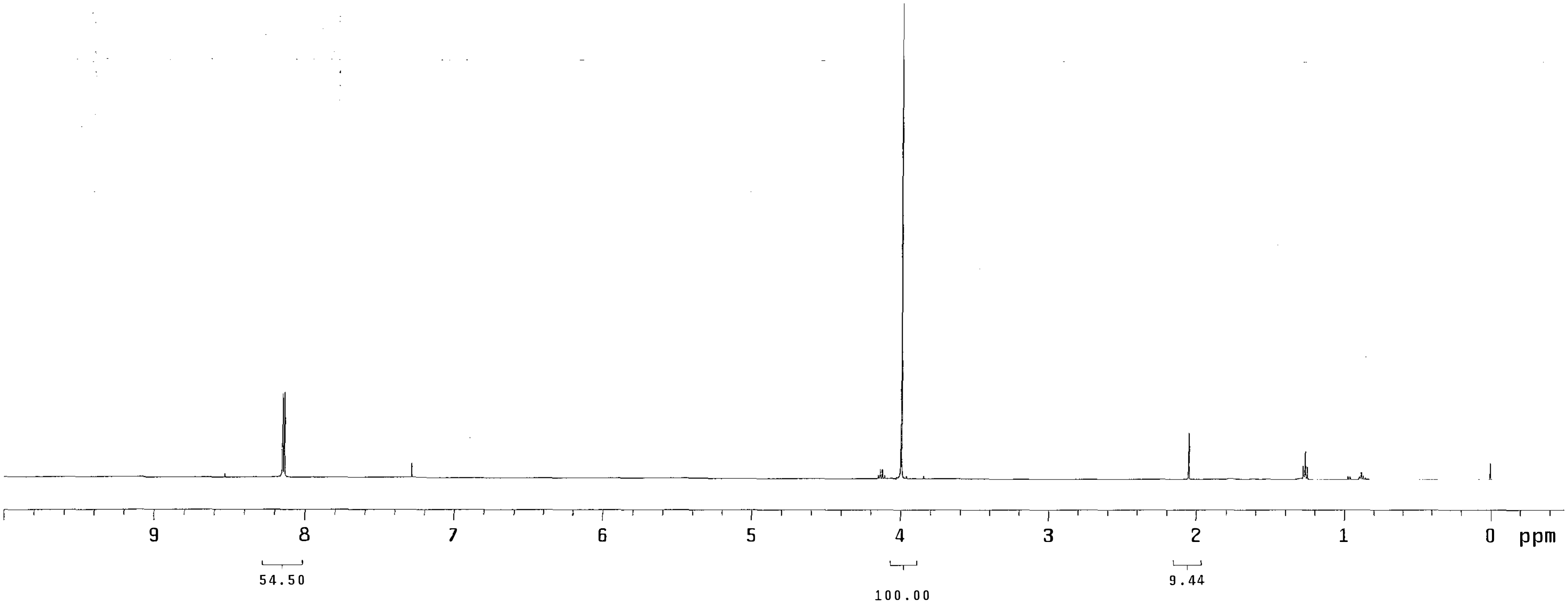

Band 1

Sample: VRS-6-111
Sample ID: s_20120926_08
File: s_20120926_08/data/cdcl3_01.fid

Pulse Sequence: s2pul
 Solvent: cdcl3
 Temp. 26.0 C / 299.1 K
Operator: walkup
File: cdcl3_01
VNMRS-500 "NMR500"

 Relax. delay 1.000 sec
 Pulse 45.0 degrees
 Acq. time 1.300 sec
 Width 30487.8 Hz
 256 repetitions
OBSERVE C13, 125.6576166 MHz
DECOUPLE H1, 499.7341220 MHz
 Power 42 dB
 continuously on
 WALTZ-16 modulated
DATA PROCESSING
 Line broadening 0.5 Hz
FT size 131072
Total time 9 min, 51 sec

2-Chloro-6-methoxypyrazine

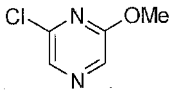

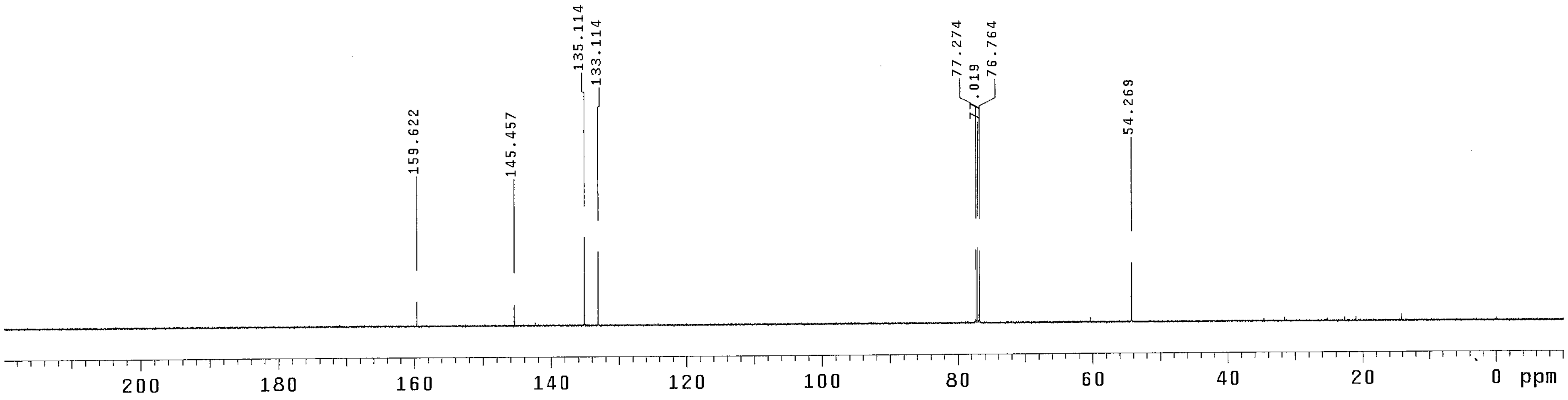

2-Methoxy-6-phenylpyrazine

Sample: VRs-6-114
Sample ID: s_20120930_01
File: s_20120930_01/data/cdcl3_01.fid

Pulse Sequence: s2pul
Solvent: cdcl3
Temp. 26.0 C / 299.1 K
Operator: walkup
File: cdcl3_01
VNMRS-500 "NMR500"

Relax. delay 1.000 sec
Pulse 45.0 degrees
Acq. time 2.049 sec
Width 8012.8 Hz
8 repetitions
OBSERVE H1, 499.7316268 MHz
DATA PROCESSING
Resol. enhancement -0.0 Hz
FT size 65536
Total time 0 min, 31 sec

2-Methoxy-6-phenylpyrazine (1d)

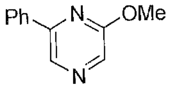

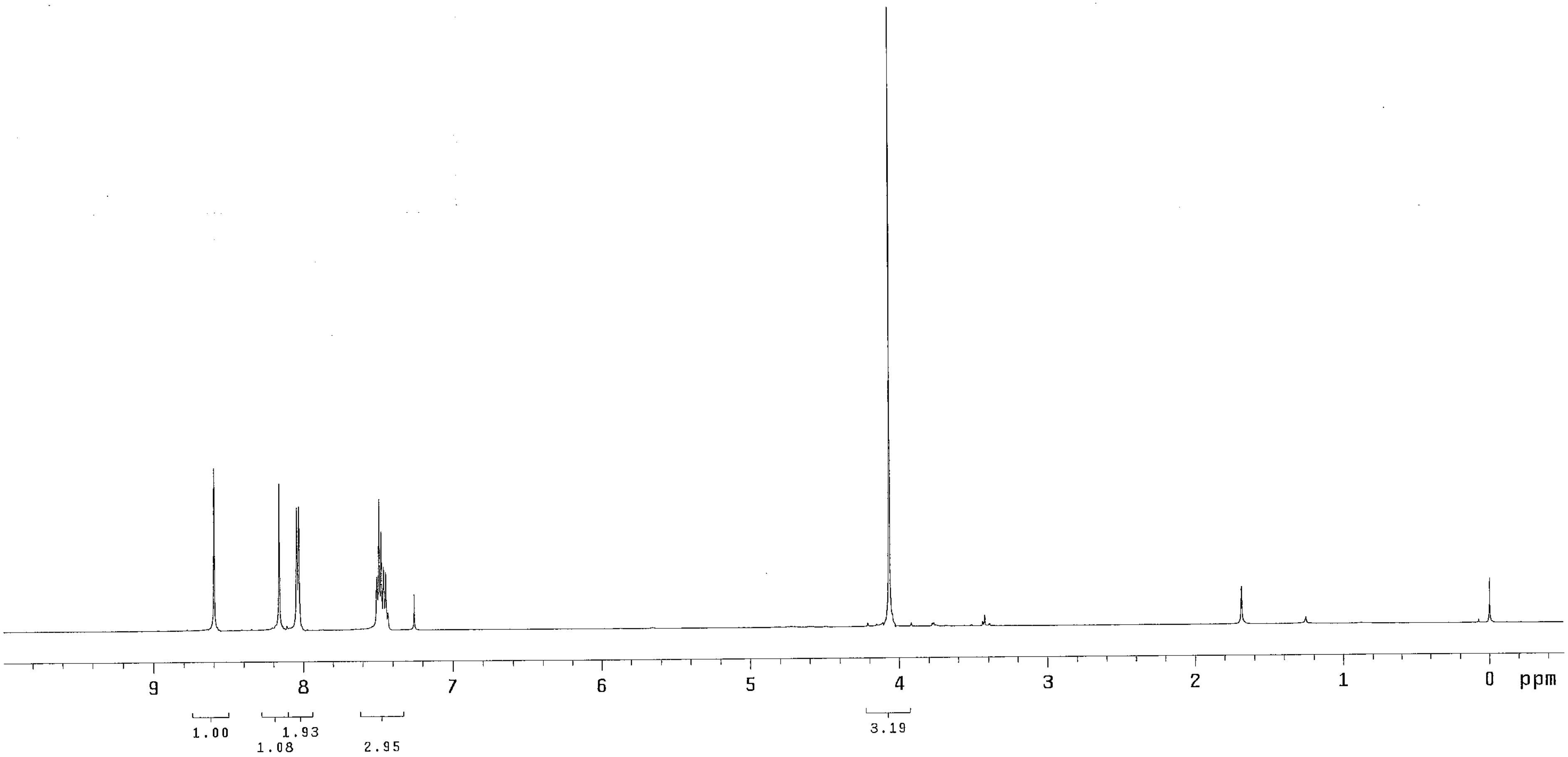

Purified (suzuki)

Sample: VRs-6-114
Sample ID: s_20120930_02
File: s_20120930_02/data/cdcl3_01.fid

Pulse Sequence: s2pul
Solvent: cdcl3
Temp. 26.0 C / 299.1 K
Operator: walkup
File: cdcl3_01
VNMRS-500 "NMR500"

Relax. delay 1.000 sec
Pulse 45.0 degrees
Acq. time 1.300 sec
Width 30487.8 Hz
256 repetitions
OBSERVE C13, 125.6576166 MHz
DECOUPLE H1, 499.7341220 MHz
Power 42 dB
continuously on
WALTZ-16 modulated
DATA PROCESSING
Line broadening 0.5 Hz
FT size 131072
Total time 9 min, 51 sec

2-Methoxy-6-phenylpyrazine (1d)

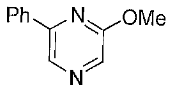

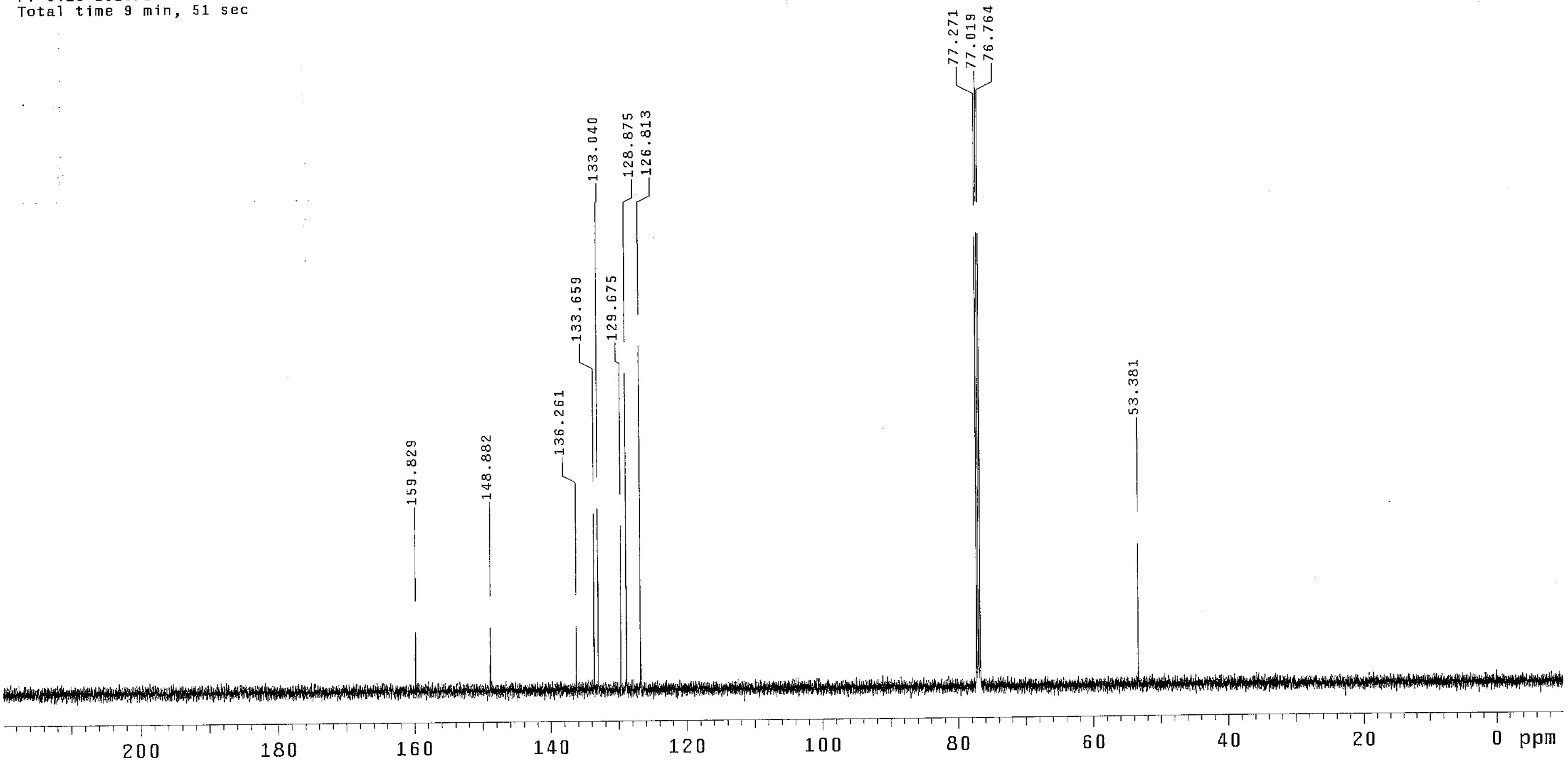

2-(Benzyloxy)-6-chloropyrazine

Sample: DBR-3-447-R
Sample ID: s_20120111_02
File: s_20120111_02/data/cdcl3_01.fid

Pulse Sequence: s2pul
Solvent: cdcl3
Temp. 26.0 C / 299.1 K
Operator: walkup
File: cdcl3_01
VNMRS-500 "NMR500"

Relax. delay 1.000 sec
Pulse 45.0 degrees
Acq. time 2.049 sec
Width 8012.8 Hz
8 repetitions
OBSERVE H1, 499.7316295 MHz
DATA PROCESSING
Resol. enhancement -0.0 Hz
FT size 65536
Total time 0 min, 31 sec

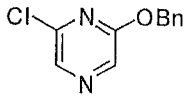

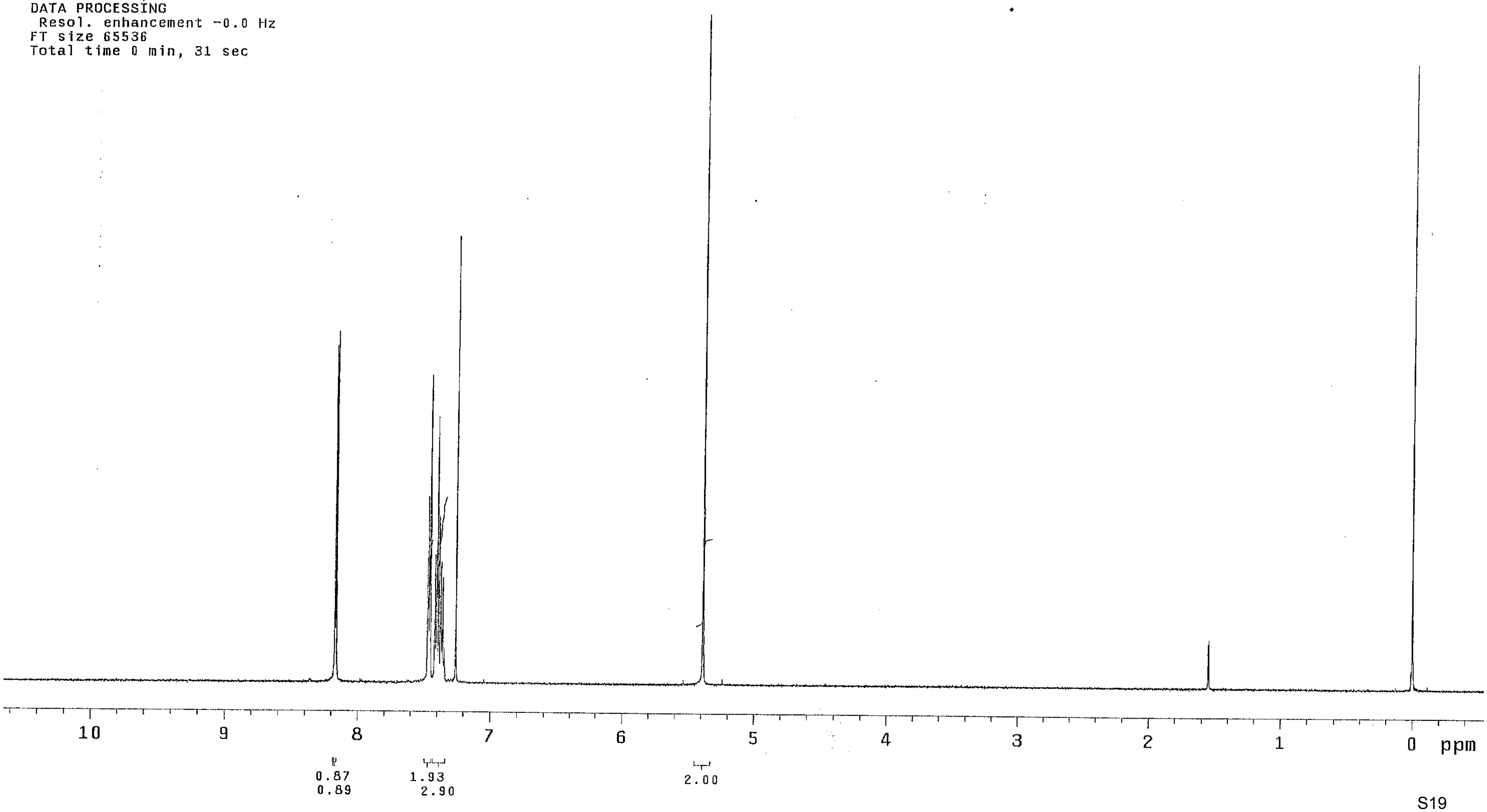

2-(Benzyloxy)-6-chloropyrazine

Sample: DBR-3-447
Sample ID: s_20101203_08
File: 00772.fid

Pulse Sequence: s2pul

Solvent: cdcl3
Temp. 26.0 C / 299.1 K
Operator: walkup
File: 00772
VNMRS-500 "NMR500"

Relax. delay 1.000 sec
Pulse 45.0 degrees
Acq. time 1.300 sec
Width 30487.8 Hz
256 repetitions
OBSERVE C13, 125.6576217 MHz
DECOUPLE H1, 499.7341220 MHz
Power 42 dB
continuously on
WALTZ-16 modulated
DATA PROCESSING
Line broadening 0.5 Hz
FT size 131072
Total time 9 min, 51 sec

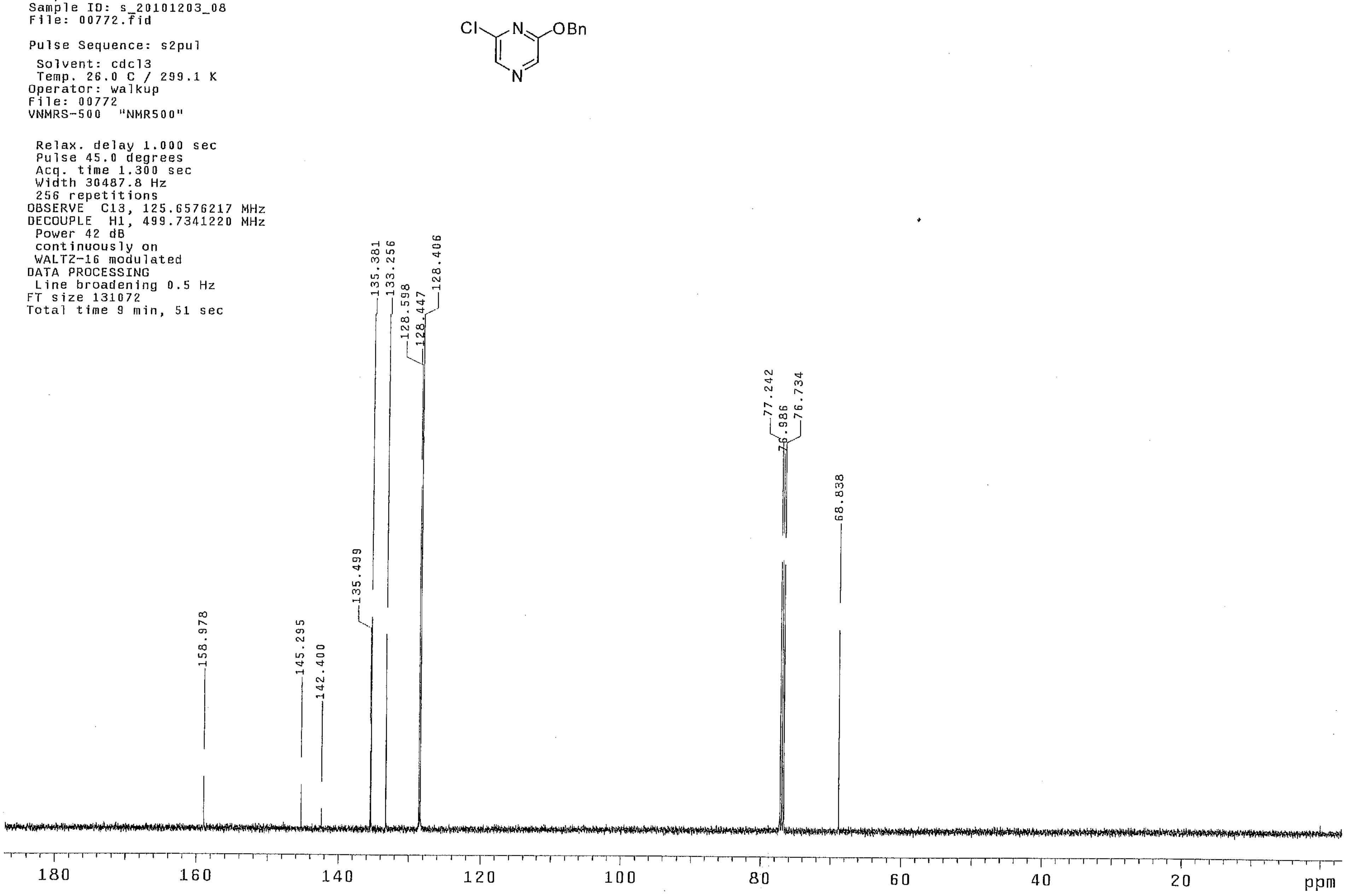

Sample: DBR-4-611
Sample ID: s_20120705_02
File: s_20120705_02/data/cdcl3_01.fid

Pulse Sequence: s2pul
Solvent: cdcl3
Temp. 26.0 C / 299.1 K
Operator: walkup
File: cdcl3_01
VNMRS-500 "NMR500"

Relax. delay 1.000 sec
Pulse 45.0 degrees
Acq. time 2.049 sec
Width 8012.8 Hz
8 repetitions
OBSERVE H1, 499.7316322 MHz
DATA PROCESSING
Resol. enhancement -0.0 Hz
FT size 65536
Total time 0 min, 31 sec

2-Benzyloxy-6-ethylpyrazine (1h)

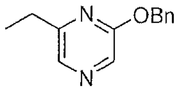

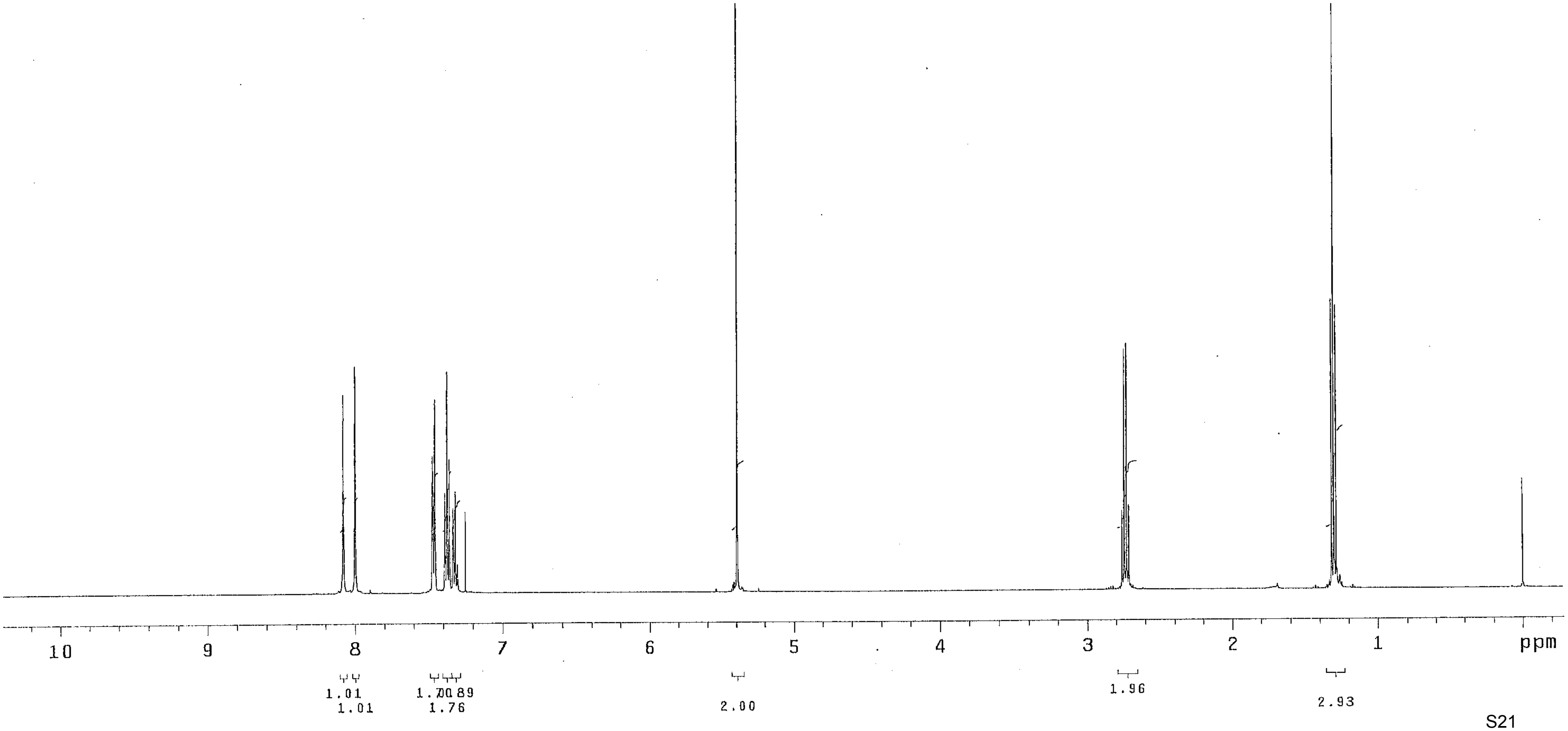

2-Benzyloxy-6-ethylpyrazine (1h)

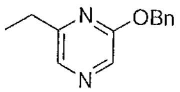

Sample: DBR-4-611
Sample ID: s_20120705_03
File: s_20120705_03/data/cdcl3_01.fid

Pulse Sequence: s2pul

Solvent: cdcl3
Temp. 26.0 C / 299.1 K
Operator: walkup
File: cdcl3_01
VNMRS-500 "NMR500"

Relax. delay 1.000 sec
Pulse 45.0 degrees
Acq. time 1.300 sec
Width 30487.8 Hz
256 repetitions
OBSERVE C13, 125.6576199 MHz
DECOUPLE H1, 499.7341220 MHz
Power 42 dB
continuously on
WALTZ-16 modulated
DATA PROCESSING
Line broadening 0.5 Hz
FT size 131072
Total time 9 min, 51 sec

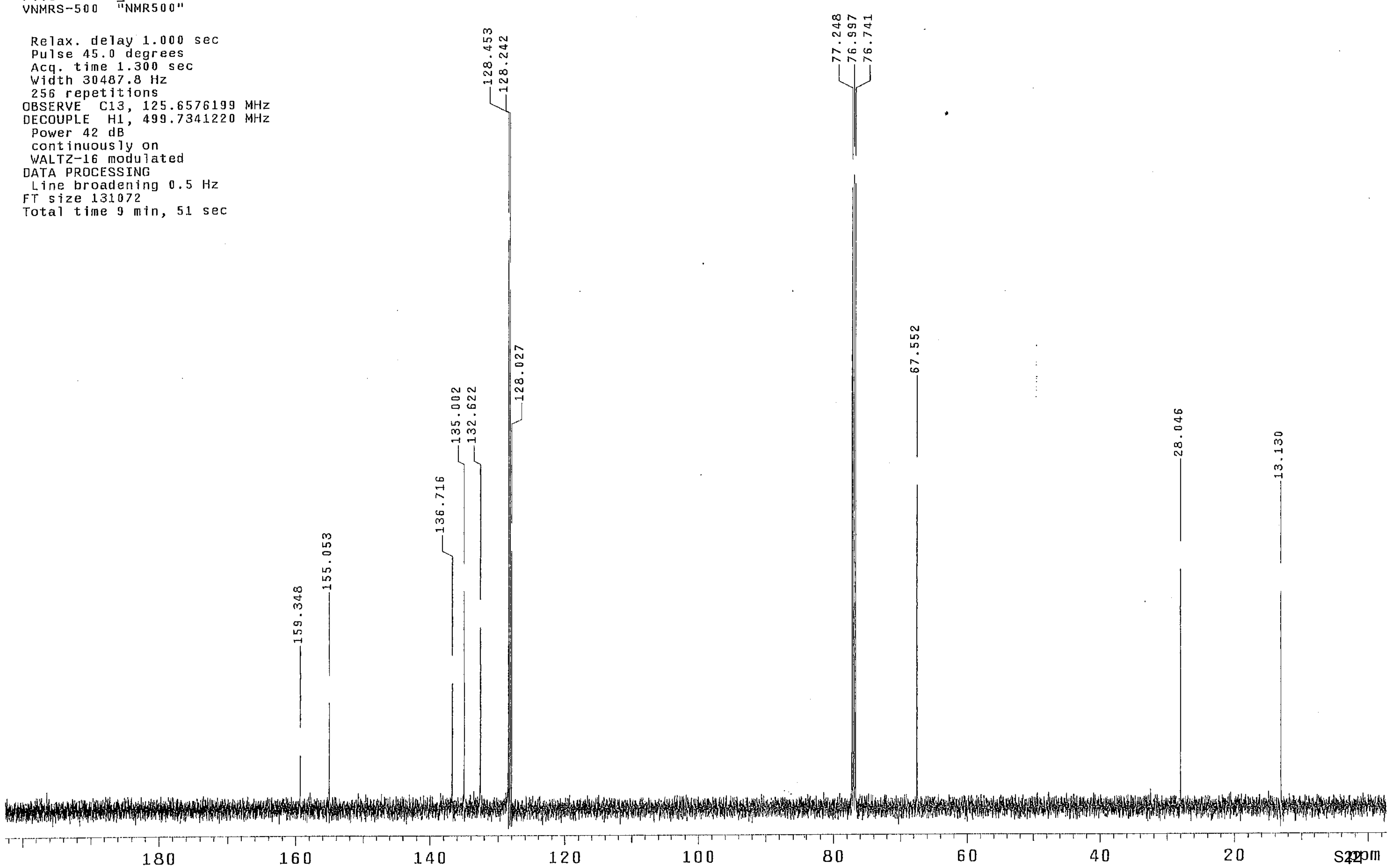

Sample: DBR-4-613
Sample ID: s_20120712_03
File: s_20120712_03/data/cdcl3_01.fid

Pulse Sequence: s2pul
Solvent: cdcl3
Temp. 26.0 C / 299.1 K
Operator: walkup
File: cdcl3_01
VNMRS-500 "NMR500"

Relax. delay 1.000 sec
Pulse 45.0 degrees
Acq. time 2.049 sec
Width 8012.8 Hz
8 repetitions
OBSERVE H1, 499.7316304 MHz
DATA PROCESSING
Resol. enhancement -0.0 Hz
FT size 65536
Total time 0 min, 31 sec

2-Benzyl-6-benzylozypyrazine (1i)

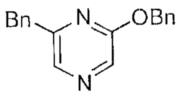

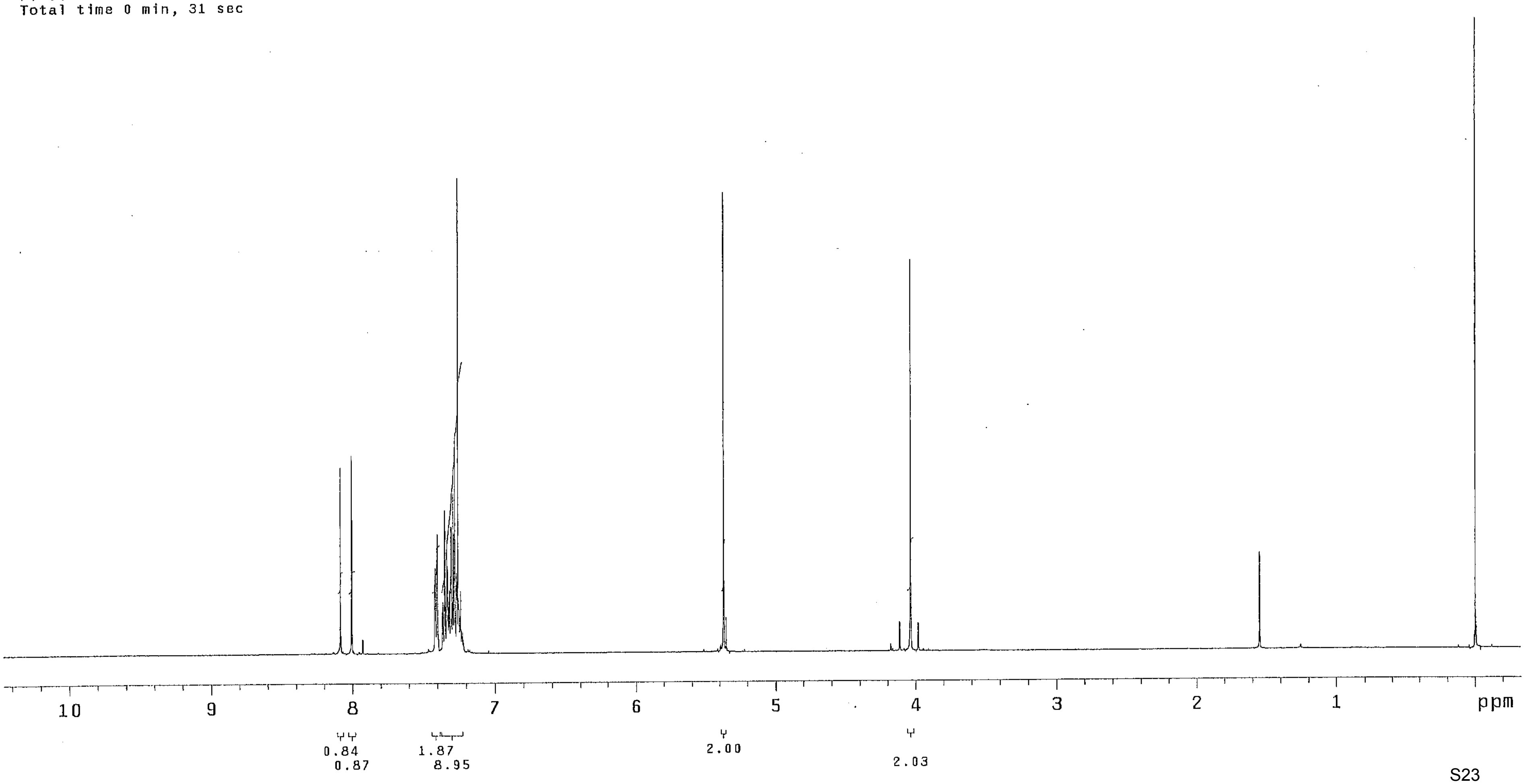

613

Sample: DBR-4-~~612~~
Sample ID: s_20120705_05
File: s_20120705_05/data/cdcl3_01.fid

Pulse Sequence: s2pul
Solvent: cdcl3
Temp. 26.0 C / 299.1 K
Operator: walkup
File: cdcl3_01
VNMRS-500 "NMR500"

Relax. delay 1.000 sec
Pulse 45.0 degrees
Acq. time 1.300 sec
Width 30487.8 Hz
256 repetitions
OBSERVE C13, 125.6576208 MHz
DECOUPLE H1, 499.7341220 MHz
Power 42 dB
continuously on
WALTZ-16 modulated
DATA PROCESSING
Line broadening 0.5 Hz
FT size 131072
Total time 9 min, 51 sec

2-Benzyl-6-benzylozypyrazine (1i)

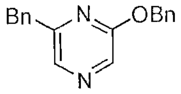

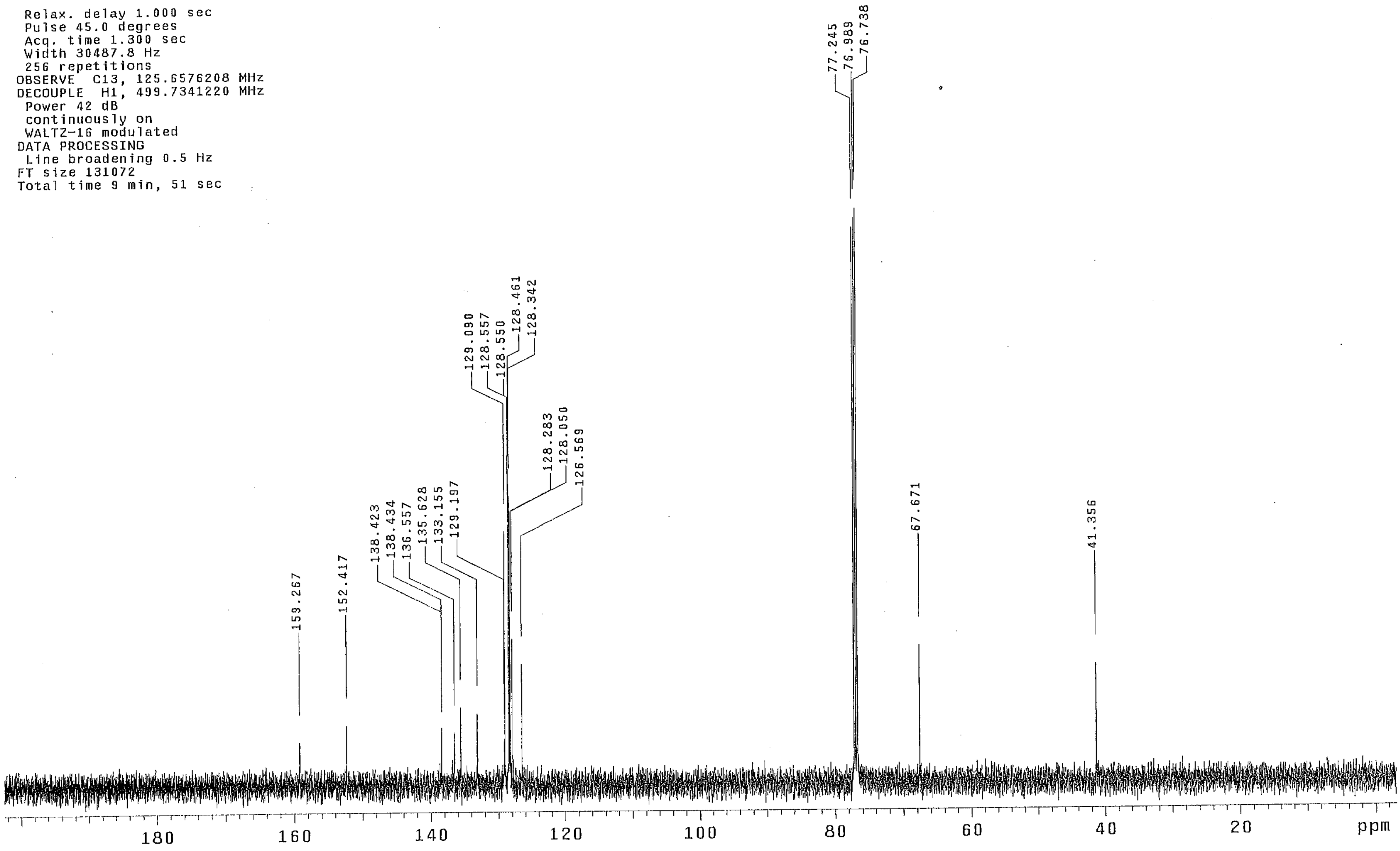

Sample: DBR-3-453
Sample ID: s_20101214_08
File: 00851.fid

Pulse Sequence: s2pul

Solvent: cdcl3
Temp. 26.0 C / 299.1 K
Operator: walkup
File: 00851
VNMRS-500 "NMR500"

Relax. delay 1.000 sec
Pulse 45.0 degrees
Acq. time 2.049 sec
Width 8012.8 Hz
8 repetitions
OBSERVE H1, 499.7316366 MHz
DATA PROCESSING
Resol. enhancement -0.0 Hz
FT size 65536
Total time 0 min, 31 sec

2-Benzyloxy-6-phenylpyrazine (1j)

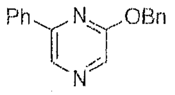

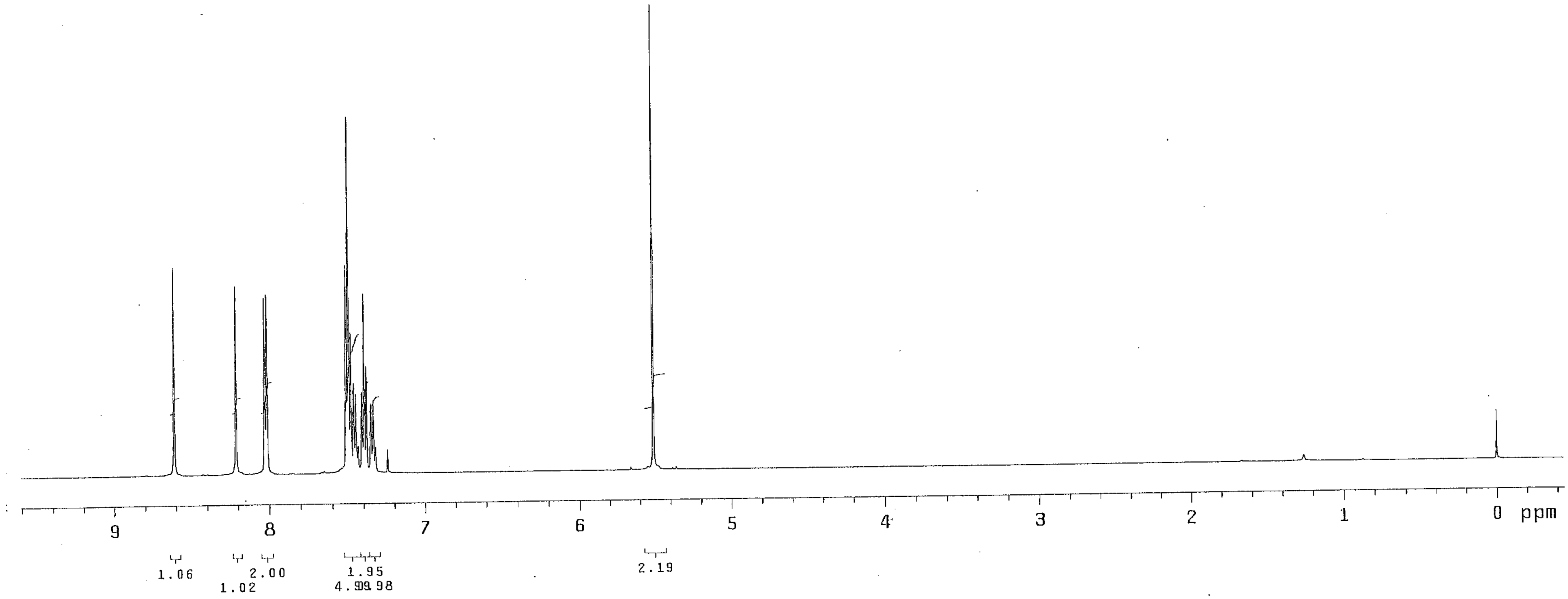

Sample: DBR-3-453
Sample ID: s_20101214_07
File: 00850.fid

Pulse Sequence: s2pul

Solvent: cdcl3
Temp. 26.0 C / 299.1 K
Operator: walkup
File: 00850
VNMRS-500 "NMR500"

Relax. delay 1.000 sec
Pulse 45.0 degrees
Acq. time 1.300 sec
Width 30487.8 Hz
256 repetitions
OBSERVE C13, 125.6576228 MHz
DECOUPLE H1, 499.7341220 MHz
Power 42 dB
continuously on
WALTZ-16 modulated
DATA PROCESSING
Line broadening 0.5 Hz
FT size 131072
Total time 9 min, 51 sec

2-Benzyloxy-6-phenylpyrazine (1j)

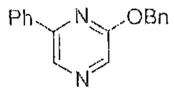

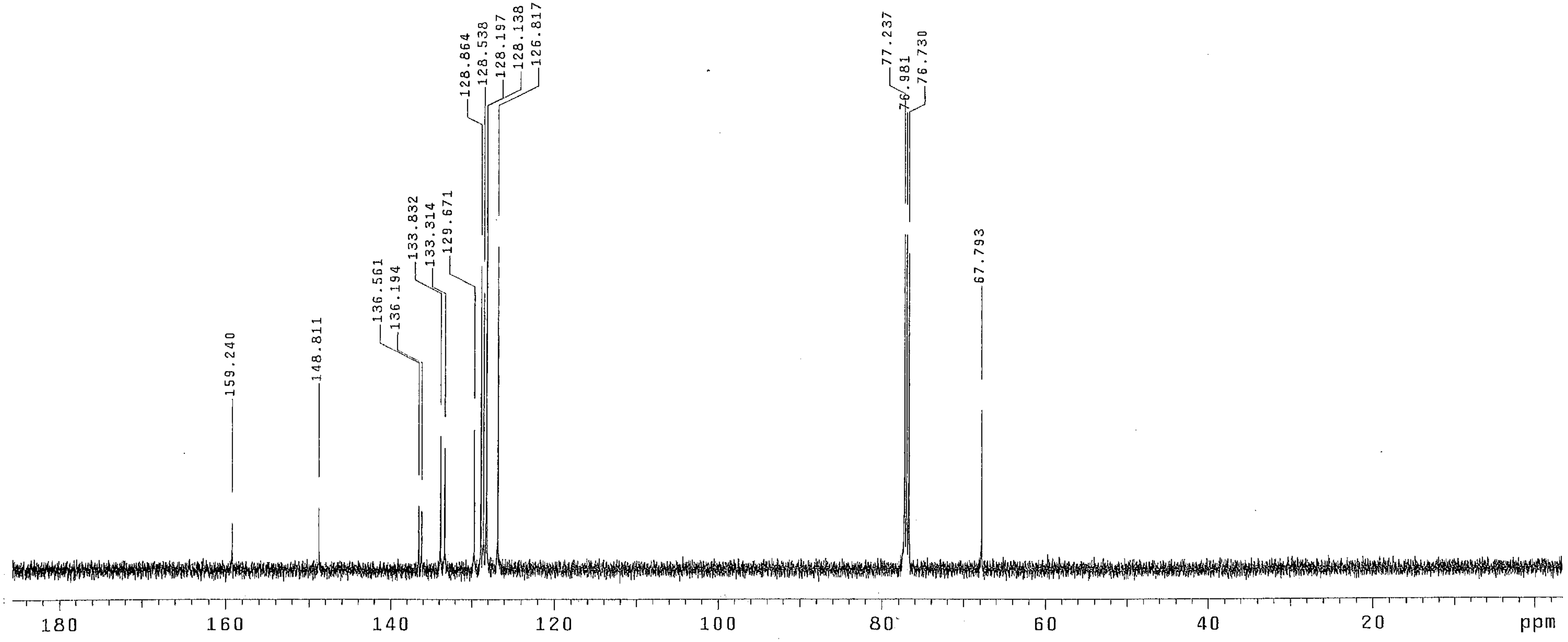

IPC 10 hr

Sample: VRS-7-57
Sample ID: s_20140113_01
File: s_20140113_01/data/cdcl3_01.fid

Pulse Sequence: s2pul

Solvent: cdcl3
Temp. 26.0 C / 299.1 K
Operator: walkup
File: cdcl3_01
VNMRS-500 "NMR500"

Relax. delay 1.000 sec
Pulse 45.0 degrees
Acq. time 2.049 sec
Width 8012.8 Hz
8 repetitions
OBSERVE H1, 499.7316285 MHz
DATA PROCESSING
Resol. enhancement -0.0 Hz
FT size 65536
Total time 0 min, 31 sec

Benzyl 6-chloropyrazine-2-carboxylate

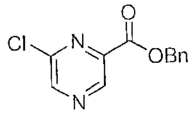

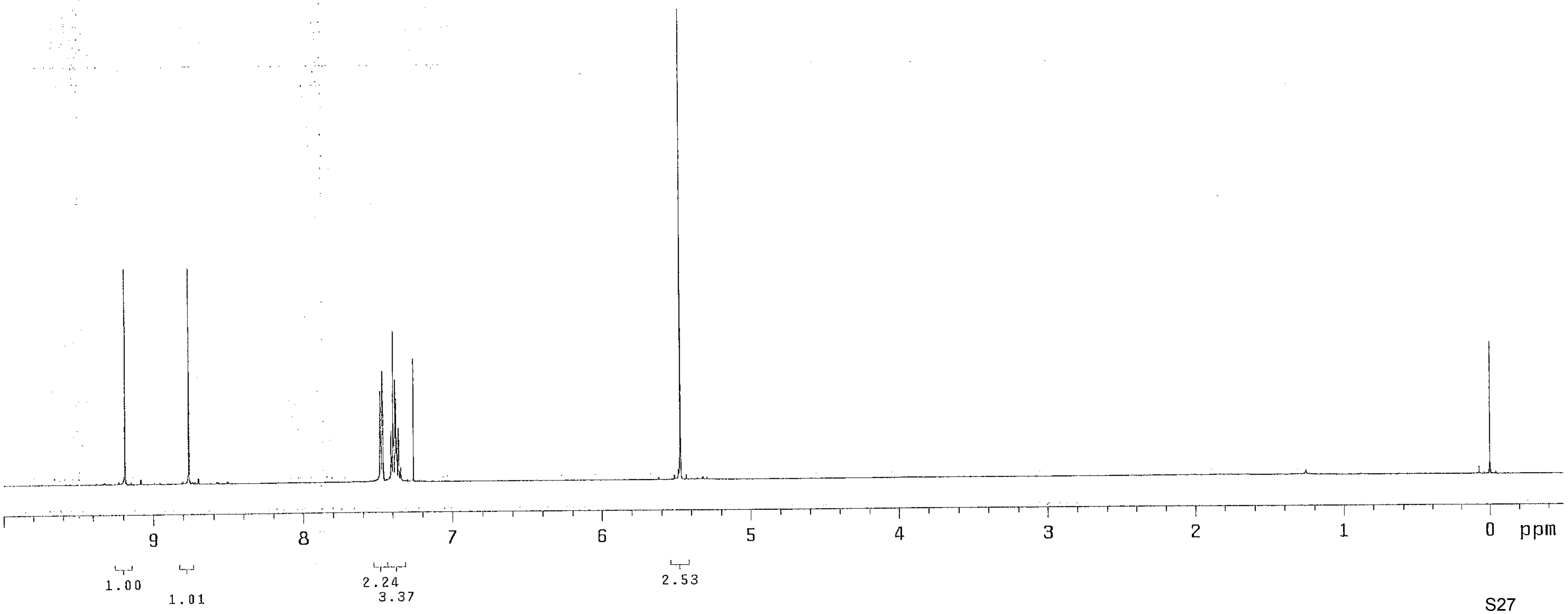

2-CBZ-6-chloropyrazine

Sample: VRS-7-57
Sample ID: s_20140113_02
File: s_20140113_02/data/cdcl3_01.fid

Pulse Sequence: s2pul

Solvent: cdcl3
Temp. 26.0 C / 299.1 K
Operator: walkup
File: cdcl3_01
VNMRS-500 "NMR500"

Relax. delay 1.000 sec
Pulse 45.0 degrees
Acq. time 1.300 sec
Width 30487.8 Hz
256 repetitions
OBSERVE C13, 125.6576166 MHz
DECOUPLE H1, 499.7341220 MHz
Power 42 dB
continuously on
WALTZ-16 modulated
DATA PROCESSING
Line broadening 0.5 Hz
FT size 131072
Total time 9 min, 51 sec

Benzyl 6-chloropyrazine-2-carboxylate

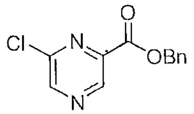

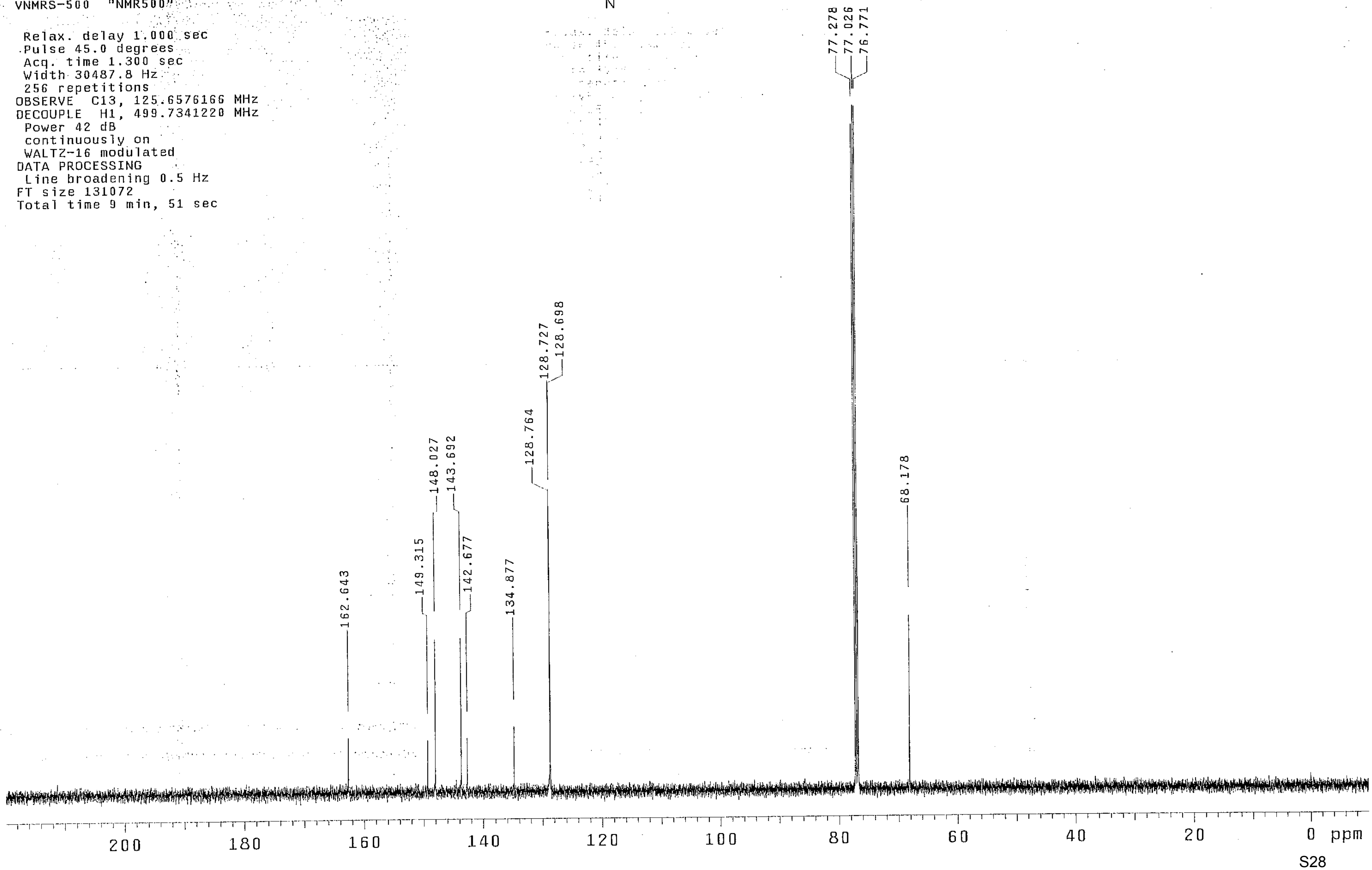

Band from ISCO

Sample: VRS-7-54
Sample ID: s_20140815_02
File: s_20140815_02/data/cdcl

Pulse Sequence: s2pul
Solvent: cdcl3
Temp. 26.0 C / 299.1 K
Operator: walkup
File: cdcl3_01
VNMRS-500 "NMR500"

Relax. delay 1.000 sec
Pulse 45.0 degrees
Acq. time 2.049 sec
Width 8012.8 Hz
8 repetitions
OBSERVE H1, 499.7316363 MHz
DATA PROCESSING
Resol. enhancement -0.0 Hz
FT size 65536
Total time 0 min, 31 sec

Benzyl 6-(benzyloxy)pyrazine-2-carboxylate (1k)

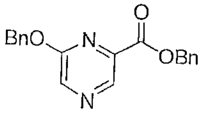

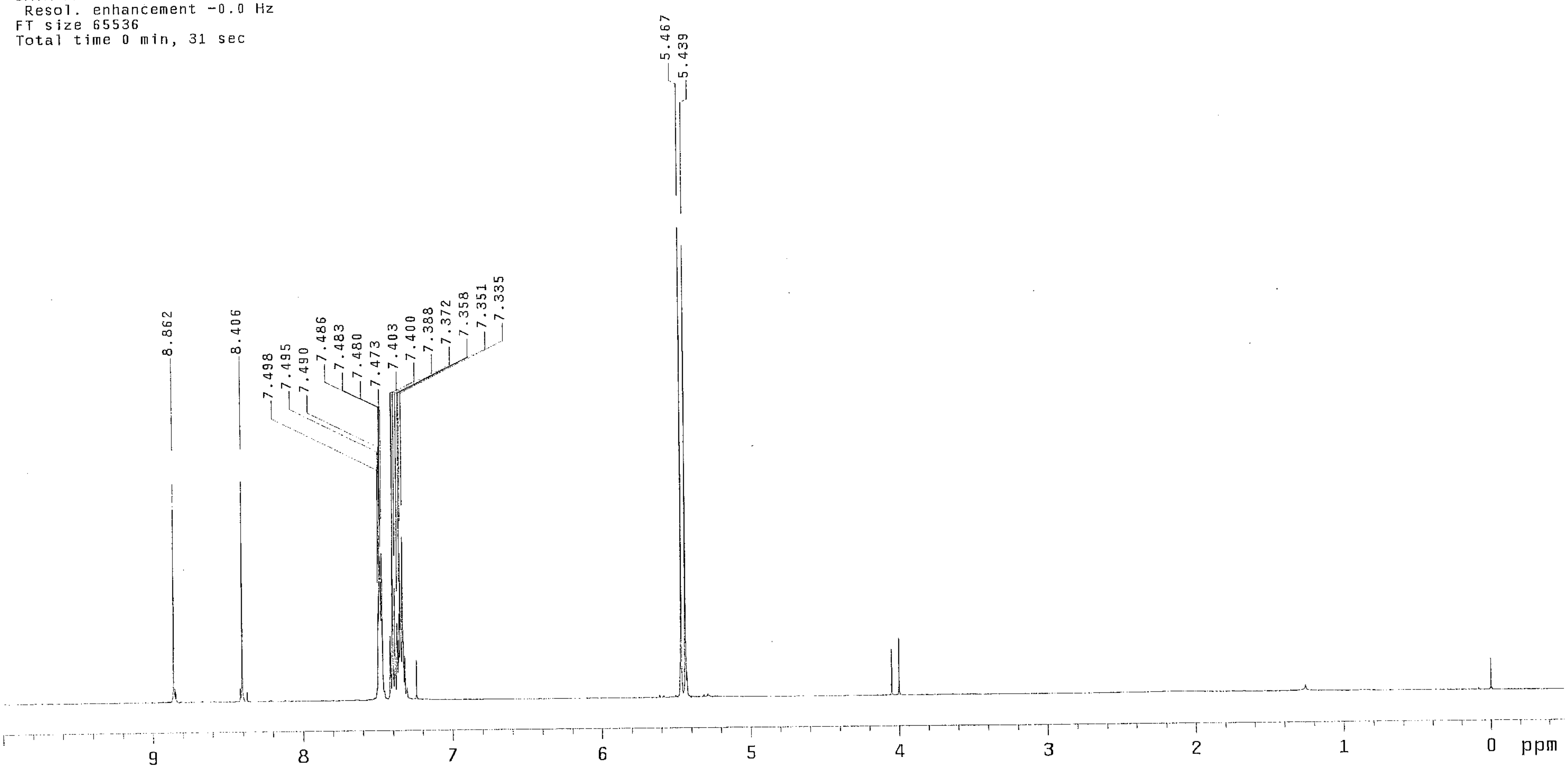

Purified

Sample: VRS-7-54
Sample ID: s_20140815_03
File: s_20140815_03/data/cdcl3_

Pulse Sequence: s2pul
Solvent: cdcl3
Temp. 26.0 C / 299.1 K
Operator: walkup
File: cdcl3_01
VNMRS-500 "NMR500"

Relax. delay 1.000 sec
Pulse 45.0 degrees
Acq. time 1.300 sec
Width 30487.8 Hz
256 repetitions
OBSERVE C13, 125.6576260 MHz
DECOUPLE H1, 499.7341220 MHz
Power 41 dB
continuously on
WALTZ-16 modulated
DATA PROCESSING
Line broadening 0.5 Hz
FT size 131072
Total time 9 min, 51 sec

Benzyl 6-(benzyloxy)pyrazine-2-carboxylate (1k)

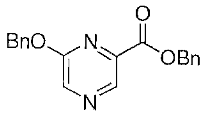

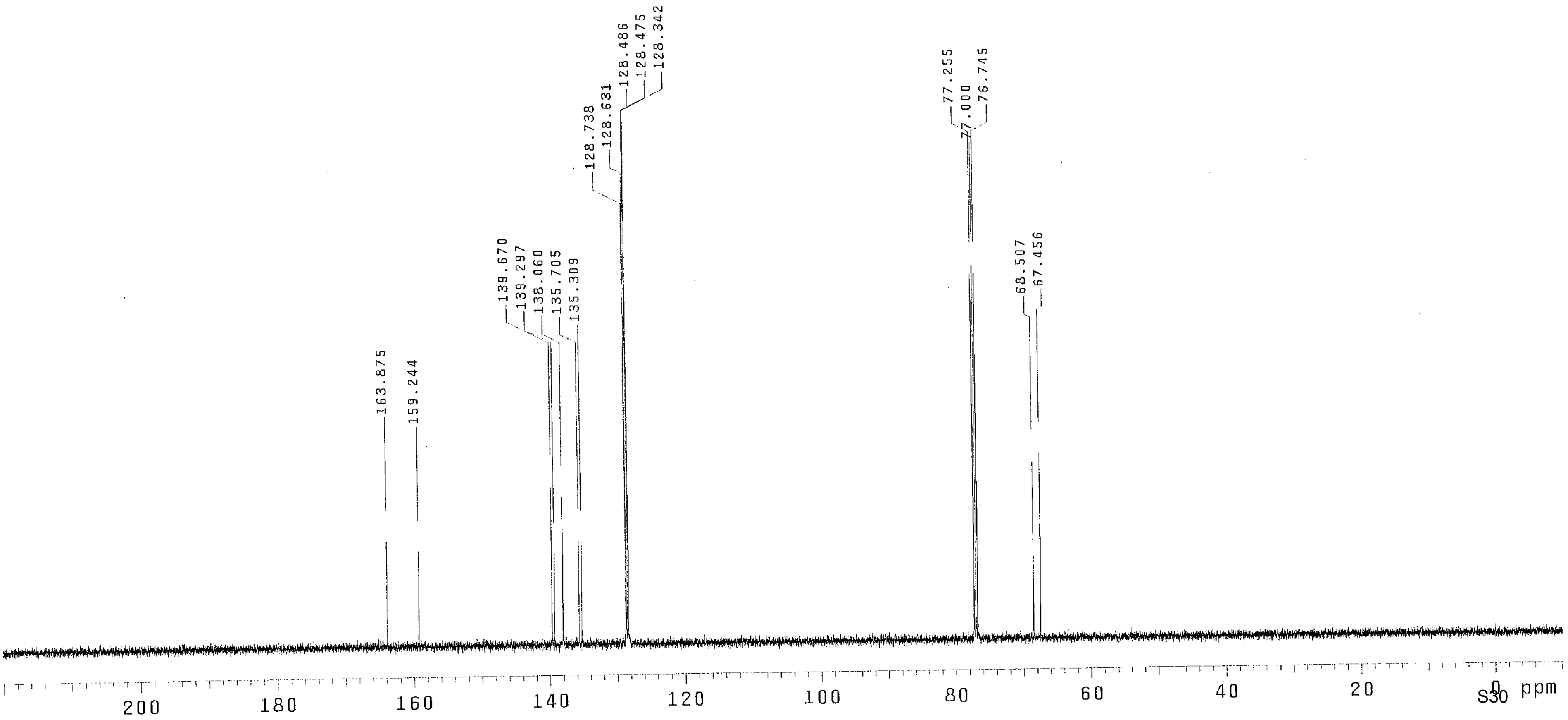

Purified

Sample: WH-1-4
Sample ID: s_20120611_08
File: s_20120611_08/data/cdcl3_01.fid

Pulse Sequence: s2pul
Solvent: cdcl3
Temp. 26.0 C / 299.1 K
Operator: walkup
File: cdcl3_01
VNMRS-500 "NMR500"

Relax. delay 1.000 sec
Pulse 45.0 degrees
Acq. time 2.049 sec
Width 8012.8 Hz
8 repetitions
OBSERVE H1, 499.7316441 MHz
DATA PROCESSING
Resol. enhancement -0.0 Hz
FT size 65536
Total time 0 min, 31 sec

1-Phenoxycarbonyl-2-phenyl-3-methoxy-1,2-dihydropyrazine (3a)

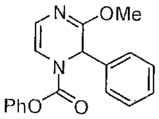

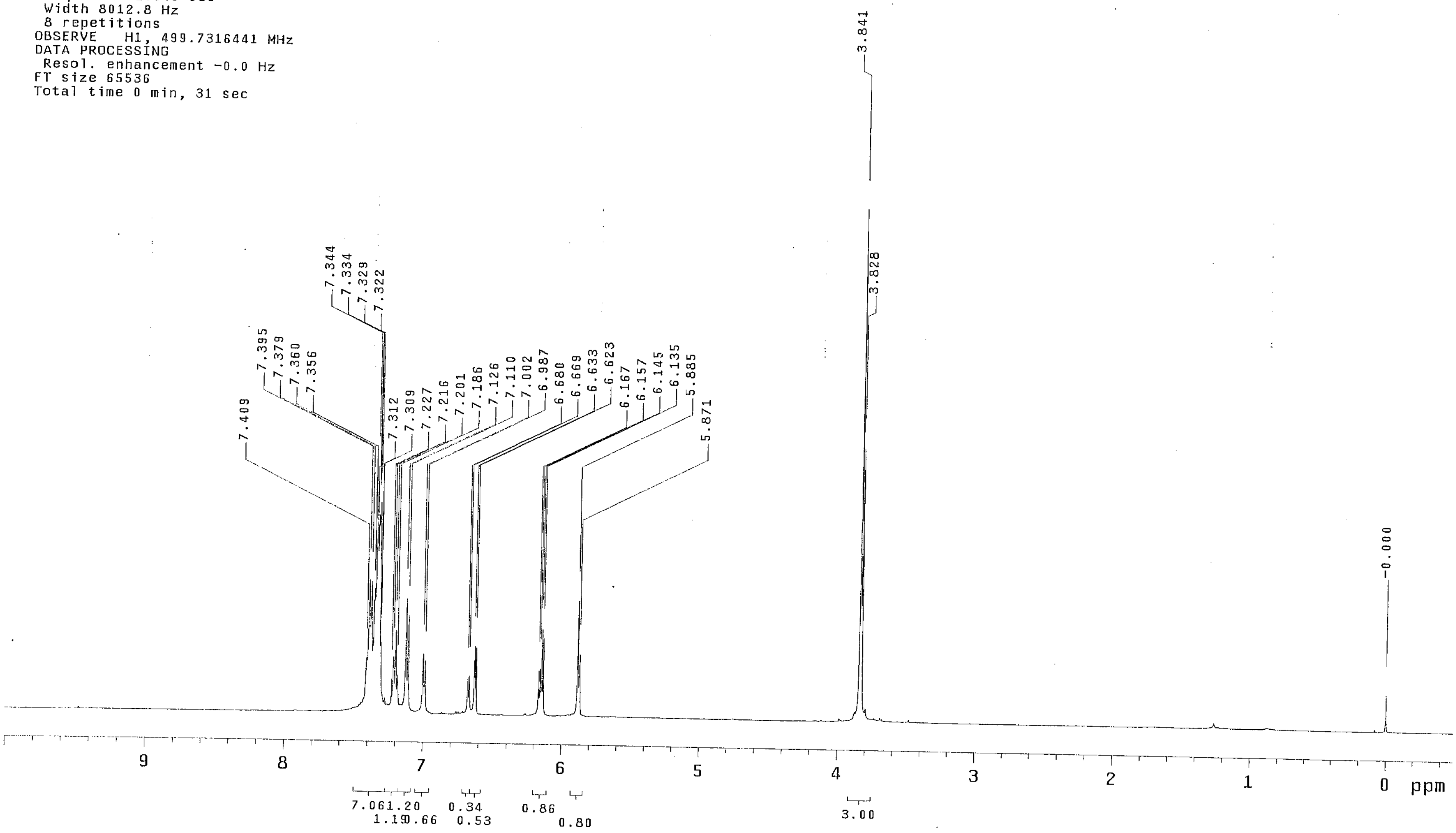

Purified

Sample: WH-1-4
File: exp

Pulse Sequence: s2pul

Solvent: cdcl3
Temp. 26.0 C / 299.1 K
Operator: walkup
VNMRS-500 "NMR500"

Relax. delay 1.000 sec
Pulse 45.0 degrees
Acq. time 1.300 sec
Width 30487.8 Hz
1280 repetitions
OBSERVE C13, 125.6576247 MHz
DECOUPLE H1, 499.7341220 MHz
Power 42 dB
continuously on
WALTZ-16 modulated
DATA PROCESSING
Line broadening 0.5 Hz
FT size 131072
Total time 16 hr, 25 min, 21 sec

1-Phenoxycarbonyl-2-phenyl-3-methoxy-1,2-dihydropyrazine (3a)

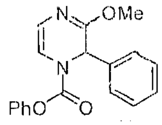

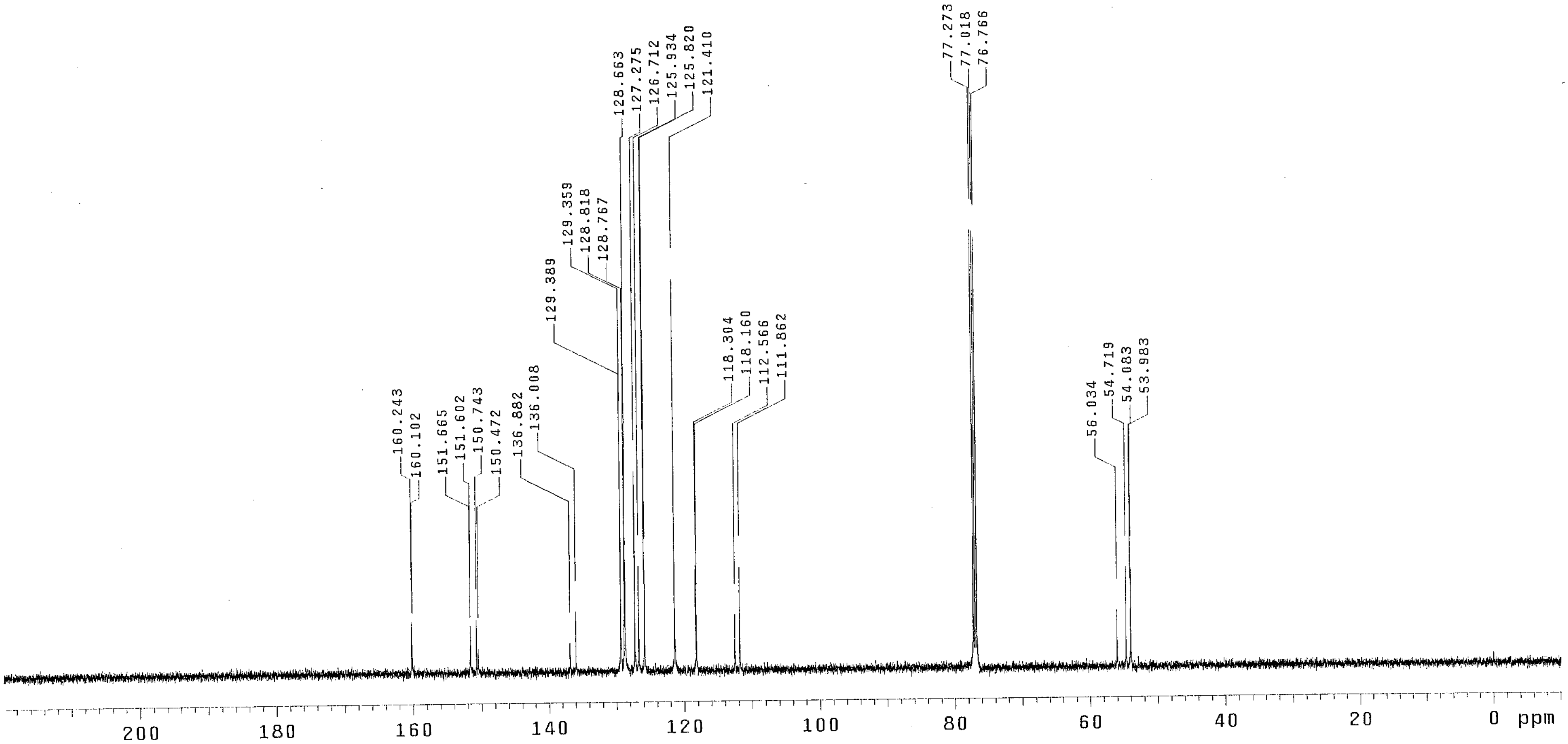

1-Phenoxycarbonyl-2-methyl-3-methoxy-1,2-dihydropyrazine

Sample: WH-1-21
Sample ID: s_20110703_04
File: /home/walkup/vnmrsys/d

Pulse Sequence: s2pul
Solvent: cdcl3
Temp. 26.0 C / 299.1 K
Operator: walkup
File: cdcl3_01
VNMRS-500 "NMR500"

Relax. delay 1.000 sec
Pulse 45.0 degrees
Acq. time 2.049 sec
Width 8012.8 Hz
8 repetitions
OBSERVE H1, 499.7316304 MHz
DATA PROCESSING
Resol. enhancement -0.0 Hz
FT size 65536
Total time 0 min, 31 sec

1-Phenoxycarbonyl-2-methyl-3-methoxy-1,2-dihydropyrazine (3b)

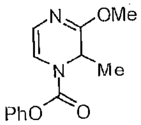

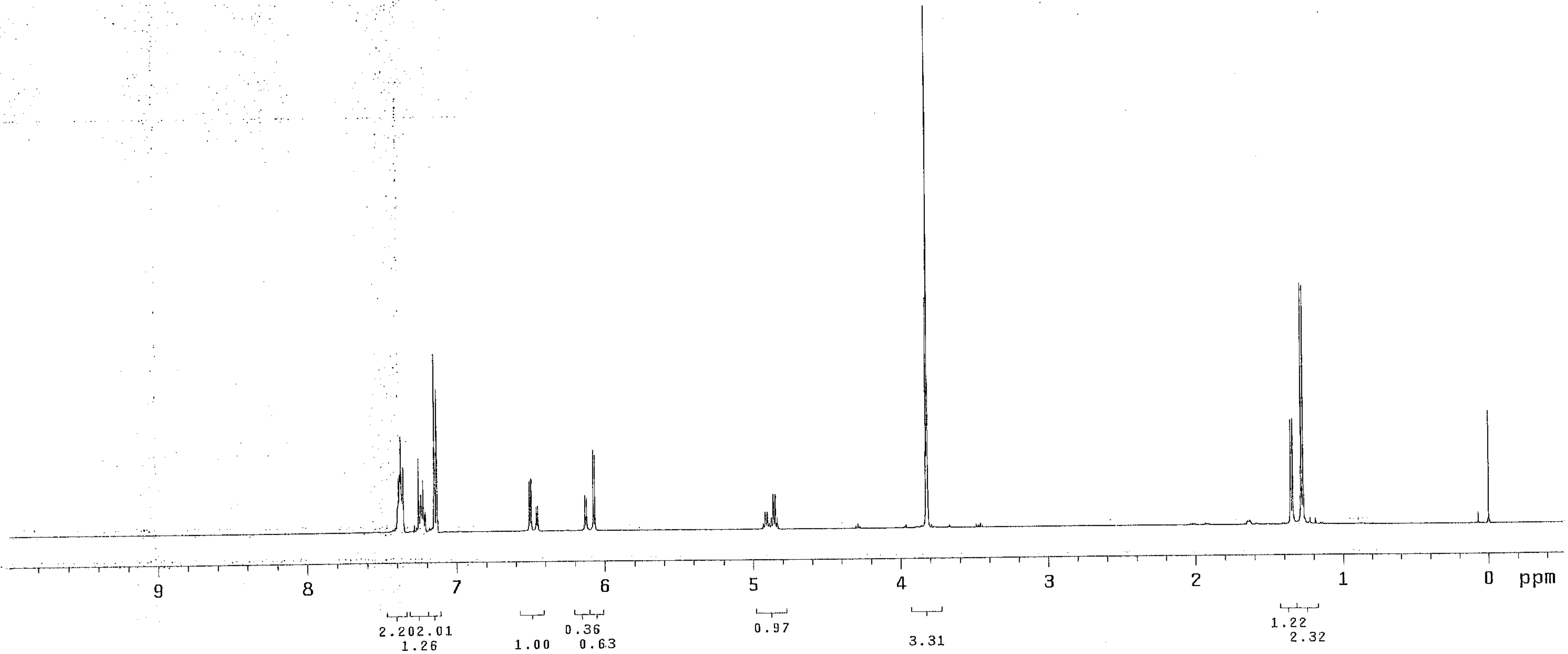

1-Phenoxycarbonyl-2-methyl-3-methoxy-1,2-dihydropyrazine

Sample: WH-1-21
Sample ID: s_20110703_03
File: /home/walkup/vnmrsys/data/

Pulse Sequence: s2pul
Solvent: cdcl3
Temp. 26.0 C / 299.1 K
Operator: walkup
File: cdcl3_01
VNMRS-500 "NMR500"

Relax. delay 1.000 sec
Pulse 45.0 degrees
Acq. time 1.300 sec
Width 30487.8 Hz
256 repetitions
OBSERVE C13, 125.6576325 MHz
DECOUPLE H1, 499.7341220 MHz
Power 42 dB
continuously on
WALTZ-16 modulated
DATA PROCESSING
Line broadening 0.5 Hz
FT size 131072
Total time 9 min, 51 sec

1-Phenoxycarbonyl-2-methyl-3-methoxy-1,2-dihydropyrazine (3b)

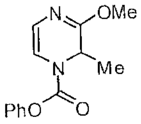

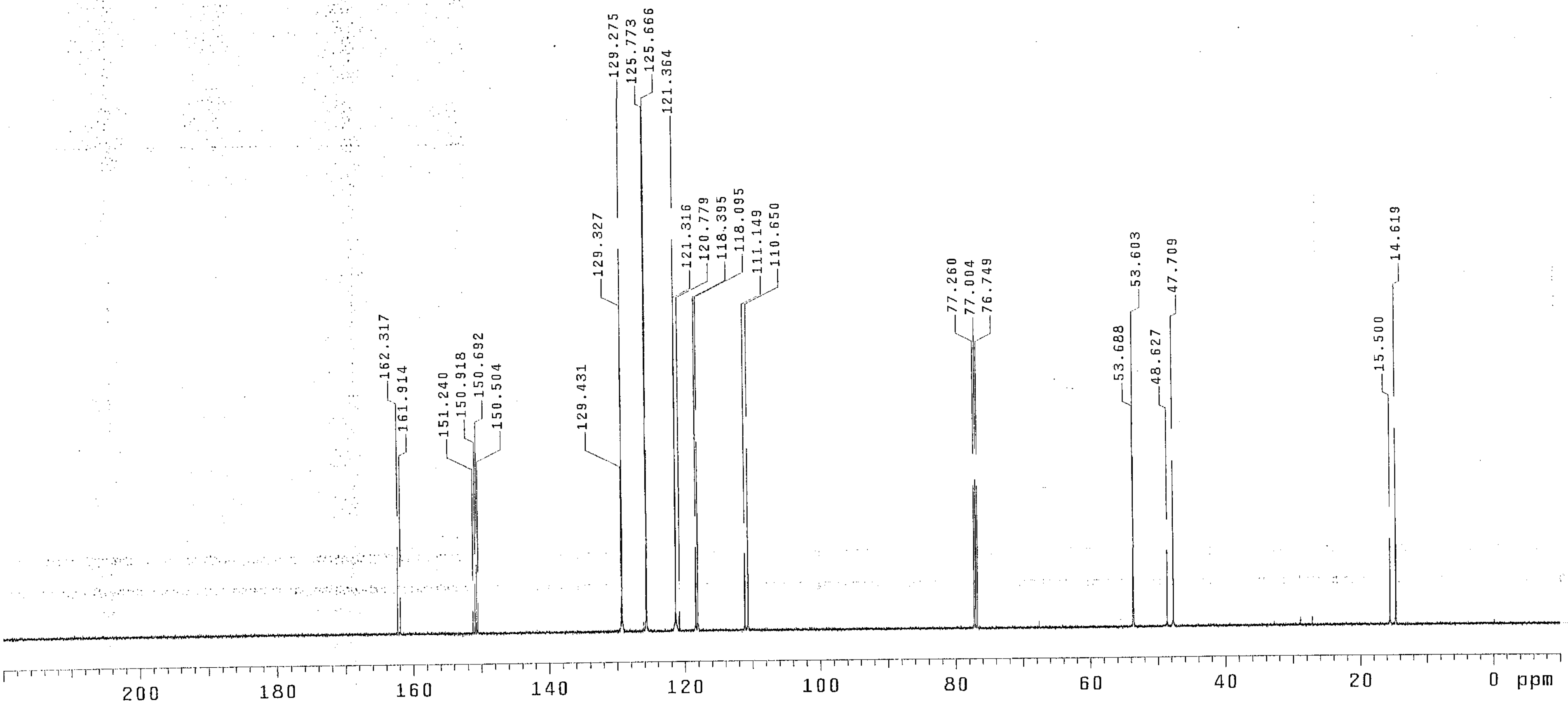

Purified (Reaction with Ethyl Grignard)

Sample: VRS-5-74B
Sample ID: s_20111122_02
File: /home/walkup/vnmrsys/data

Pulse Sequence: s2pul
Solvent: cdcl3
Temp. 26.0 C / 299.1 K
Operator: walkup
File: cdcl3_01
VNMRS-500 "NMR500"

Relax. delay 1.000 sec
Pulse 45.0 degrees
Acq. time 2.049 sec
Width 8012.8 Hz
8 repetitions
OBSERVE H1, 499.7316314 MHz
DATA PROCESSING
Resol. enhancement -0.0 Hz
FT size 65536
Total time 0 min, 31 sec

1-Phenoxycarbonyl-2-ethyl-3-methoxy-1,2-dihydropyrazine (3c)

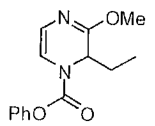

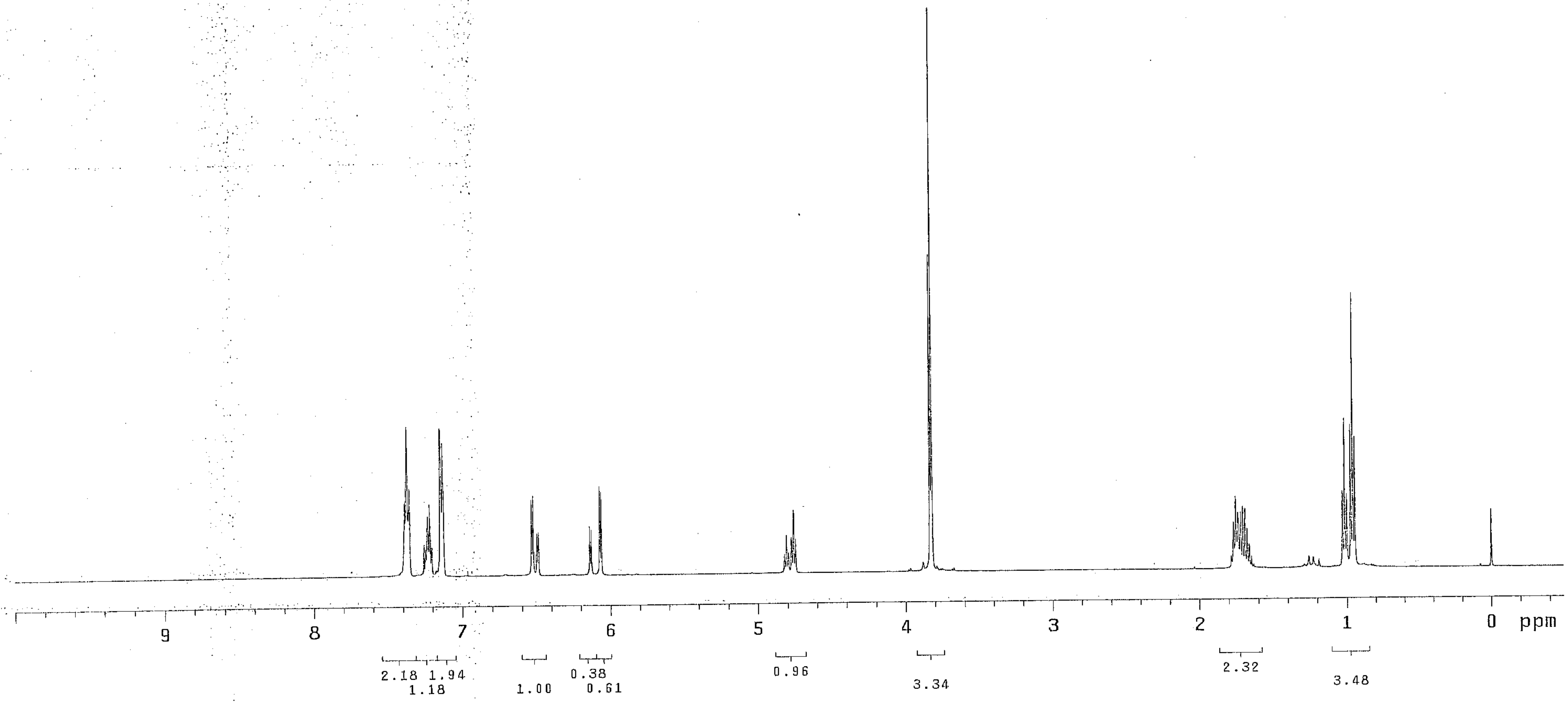

Purified (Reaction with Ethyl Grigna

Sample: VRS-5-74B
Sample ID: s_20111122_03
File: /home/walkup/vnmrsys/data/aut

Pulse Sequence: s2pul
Solvent: cdcl3
Temp. 26.0 C / 299.1 K
Operator: walkup
File: cdcl3_01
VNMRS-500 "NMR500"

Relax. delay 1.000 sec
Pulse 45.0 degrees
Acq. time 1.300 sec
Width 30487.8 Hz
256 repetitions
OBSERVE C13, 125.6576166 MHz
DECOUPLE H1, 499.7341220 MHz
Power 42 dB
continuously on
WALTZ-16 modulated
DATA PROCESSING
Line broadening 0.5 Hz
FT size 131072
Total time 9 min, 51 sec

1-Phenoxycarbonyl-2-ethyl-3-methoxy-1,2-dihydropyrazine (3c)

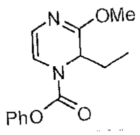

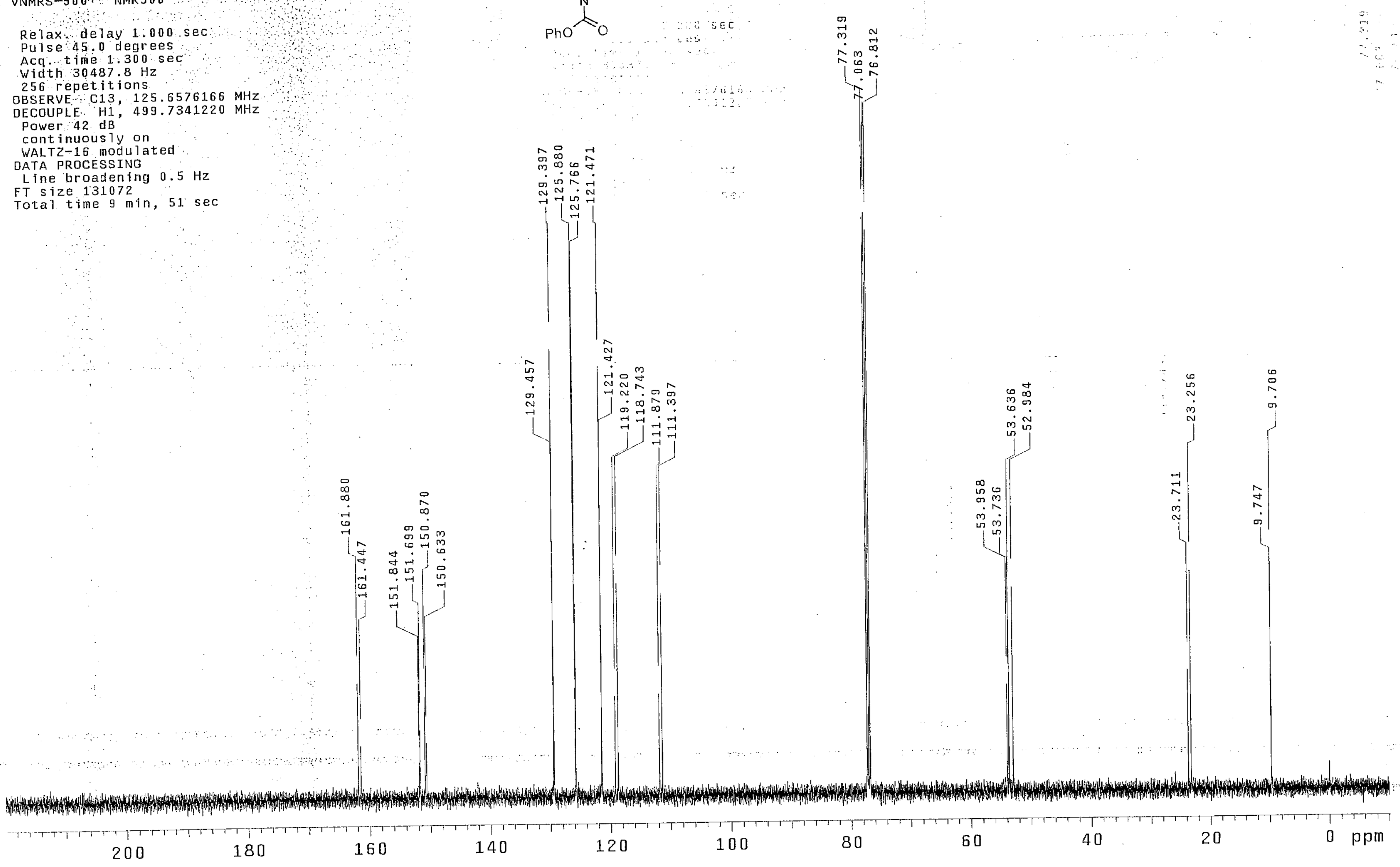

Fractions 17-25

Sample: YM-1-10
Sample ID: s_20120907_07
File: /home/walkup/vnmrsys/data

Pulse Sequence: s2pul

Solvent: cdcl3
Temp. 26.0 C / 299.1 K
Operator: walkup
File: cdcl3_01
VNMRS-500 "NMR500"

Relax. delay 1.000 sec
Pulse 45.0 degrees
Acq. time 2.049 sec
Width 8012.8 Hz
8 repetitions
OBSERVE H1, 499.7316334 MHz
DATA PROCESSING
Resol. enhancement -0.0 Hz
FT size 65536
Total time 0 min, 31 sec

1-Phenoxycarbonyl-2-isopropyl-3-methoxy-1,2-dihydropyrazine (3d)

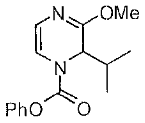

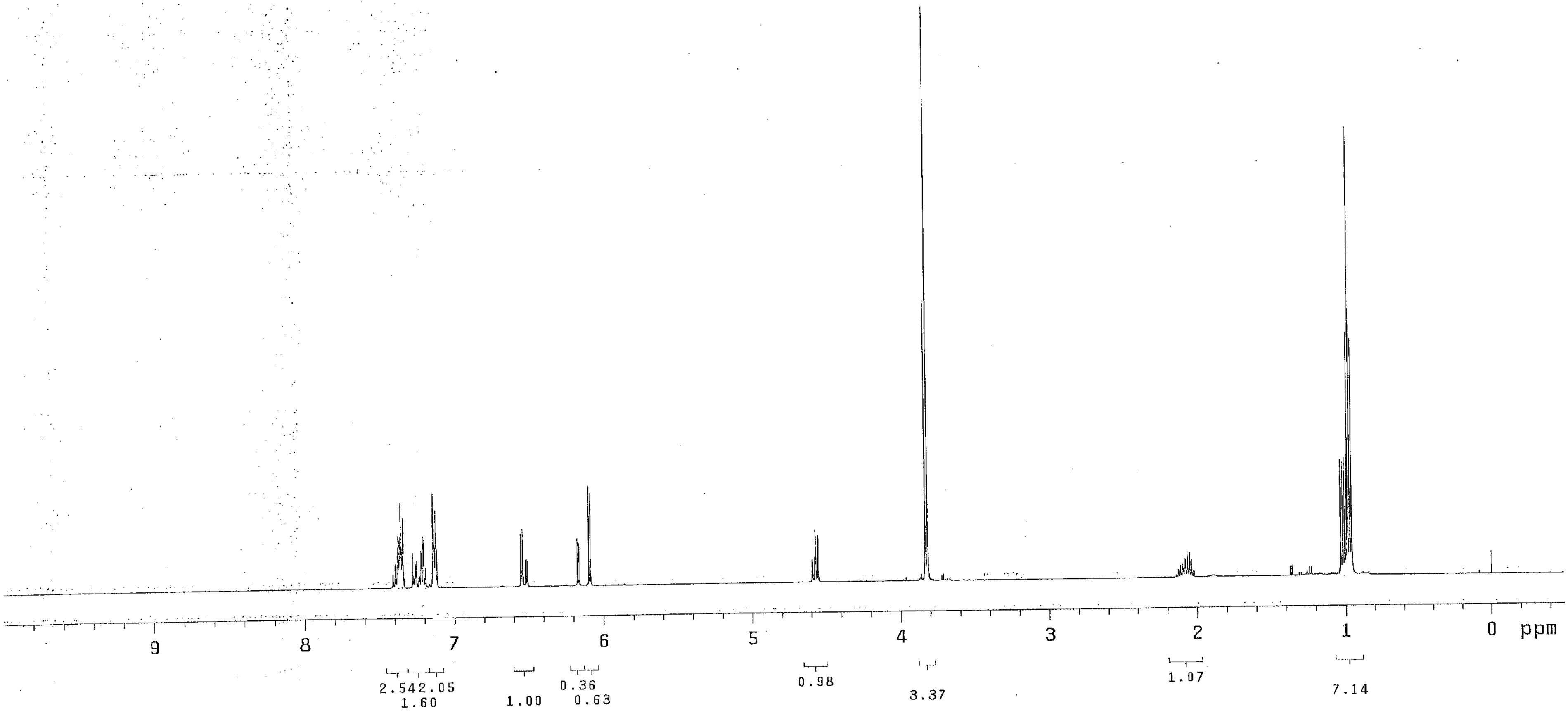

Fractions 17-25

Sample: YM-1-10
Sample ID: s_20120907_08
File: /home/walkup/vnmrsys/

Pulse Sequence: s2pul
Solvent: cdcl3
Temp. 26.0 C / 299.1 K
Operator: walkup
File: cdcl3_01
VNMRS-500 "NMR500"

Relax. delay 1.000 sec
Pulse 45.0 degrees
Acq. time 1.300 sec
Width 30487.8 Hz
256 repetitions
OBSERVE C13, 125.6576166 MHz
DECOUPLE H1, 499.7341220 MHz
Power 42 dB
continuously on
WALTZ-16 modulated
DATA PROCESSING
Line broadening 0.5 Hz
FT size 131072
Total time 9 min, 51 sec

1-Phenoxycarbonyl-2-isopropyl-3-methoxy-1,2-dihydropyrazine (3d)

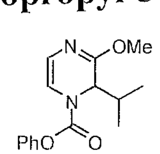

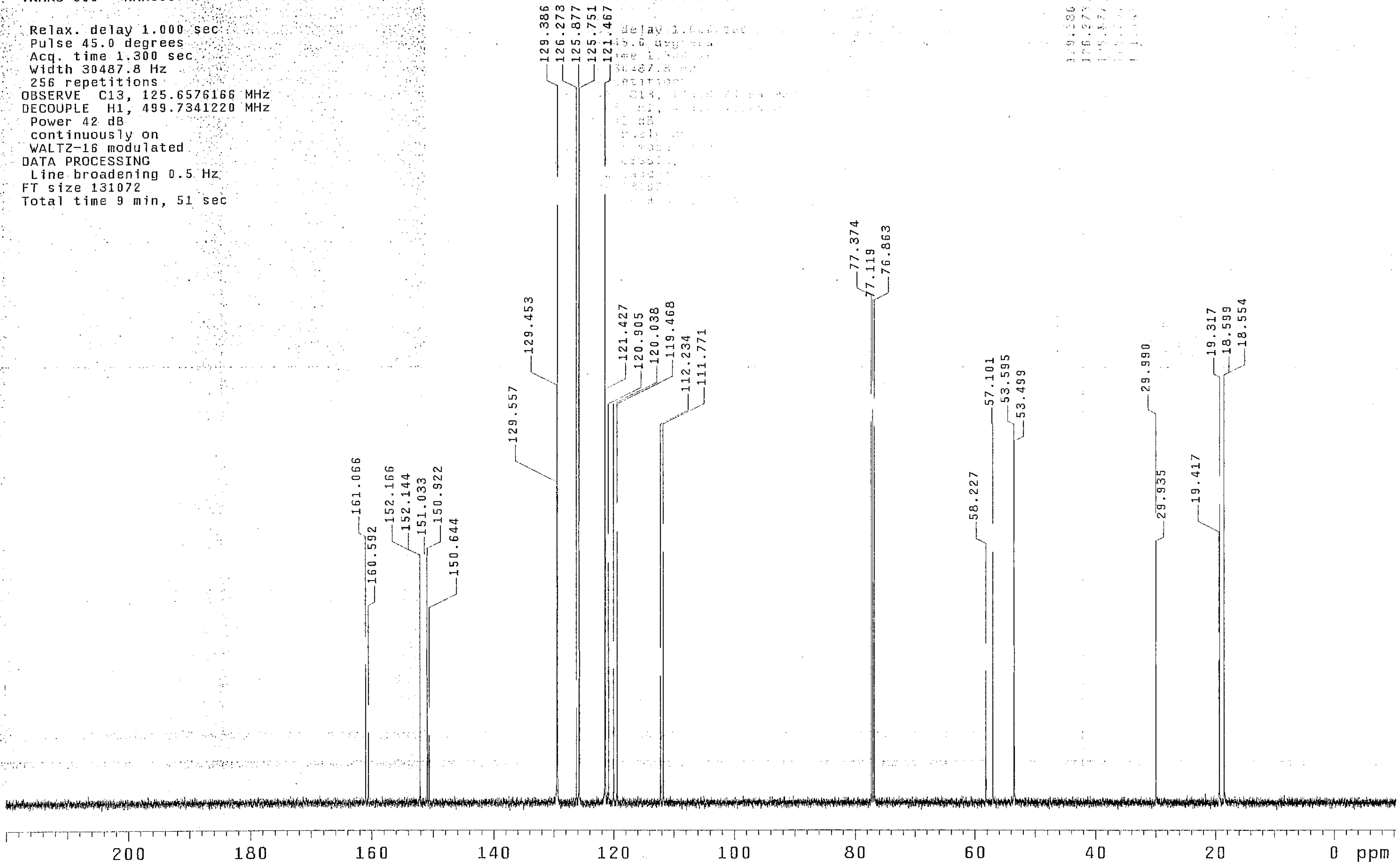

Band 1

Sample: YM-1-13
Sample ID: s_20120926_05
File: /home/walkup/vnmrsys/da

Pulse Sequence: s2pul

Solvent: cdcl3
Temp. 26.0 C / 299.1 K
Operator: walkup
File: cdcl3_01
VNMRS-500 "NMR500"

Relax. delay 1.000 sec
Pulse 45.0 degrees
Acq. time 2.049 sec
Width 8012.8 Hz
8 repetitions
OBSERVE H1, 499.7316295 MHz
DATA PROCESSING
Resol. enhancement -0.0 Hz
FT size 65536
Total time 0 min, 31 sec

1-Phenoxycarbonyl-2-cyclopentyl-3-methoxy-1,2-dihydropyrazine (3e)

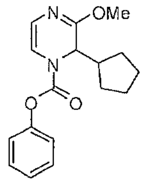

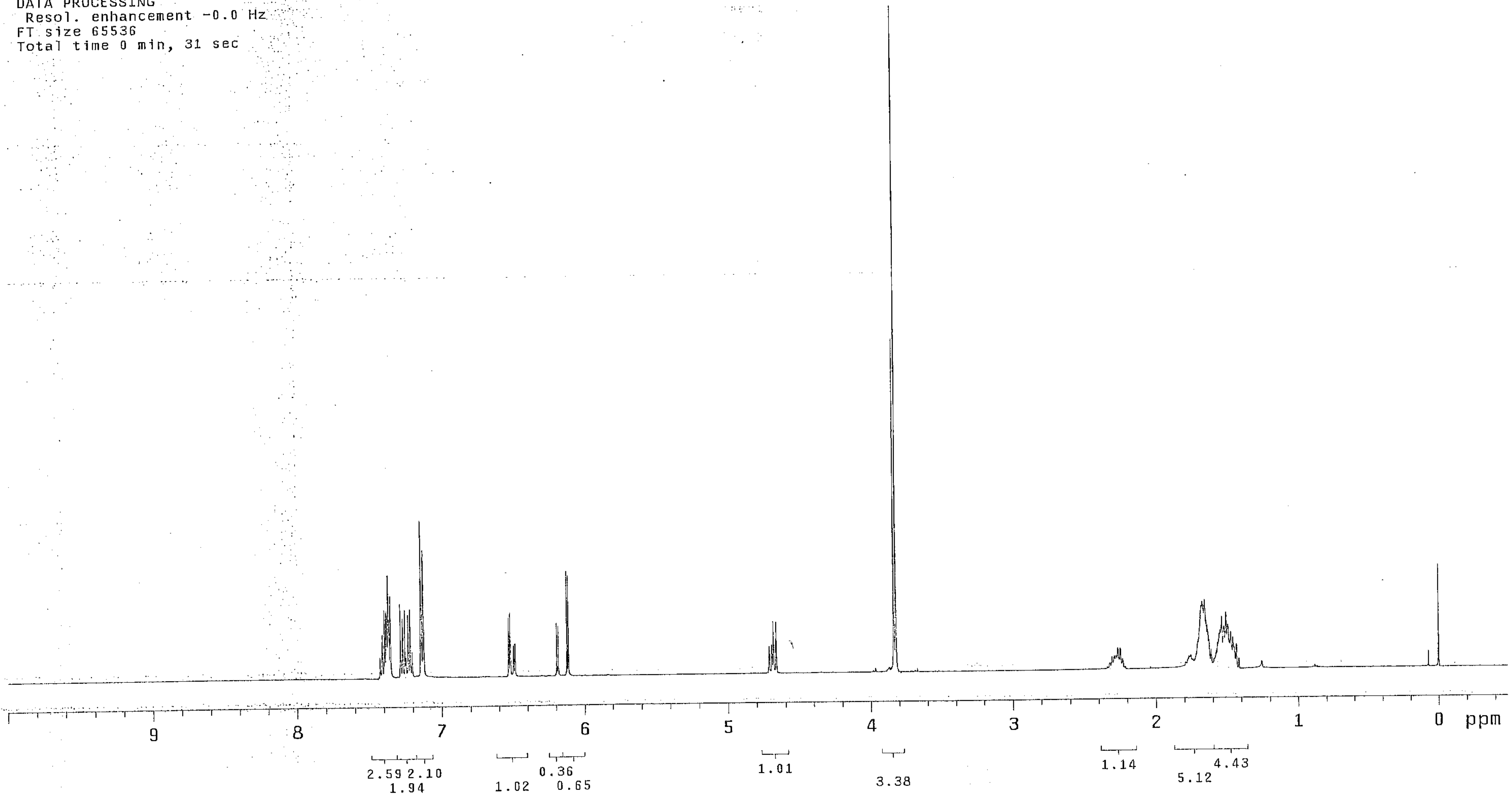

1-Phenoxycarbonyl-2-cyclopentyl-3-methoxy-1,2-dihydropyrazine (3e)

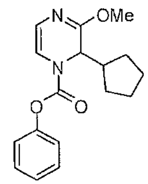

Band 1

Sample: YM-1-13
Sample ID: s_20120926_06
File: /home/walkup/vnmrsys/d

Pulse Sequence: s2pul
Solvent: cdcl3
Temp. 26.0 C / 299.1 K
Operator: walkup
File: cdcl3_01
VNMRS-500 "NMR500"

Relax. delay 1.000 sec
Pulse 45.0 degrees
Acq. time 1.300 sec
Width 30487.8 Hz
256 repetitions
OBSERVE C13, 125.6576166 MHz
DECOUPLE H1, 499.7341220 MHz
Power 42 dB
continuously on
WALTZ-16 modulated
DATA PROCESSING
Line broadening 0.5 Hz
FT size 131072
Total time 9 min, 51 sec

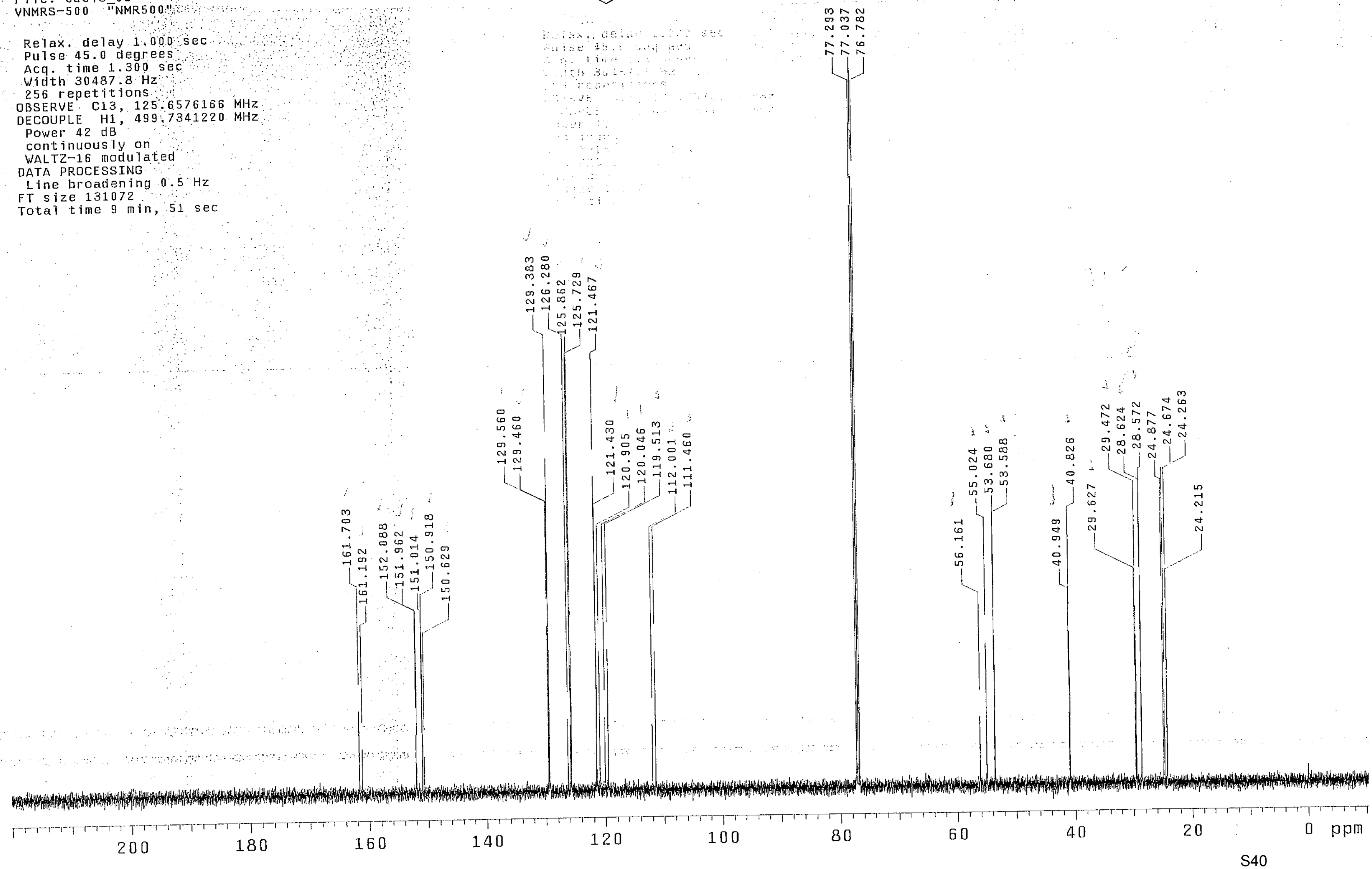

1-Phenoxycarbonyl-2-benzyl-3·

Sample: VRS-6-49B
Sample ID: s_20120611_10
File: s_20120611_10/data/cdc

Pulse Sequence: s2pul
Solvent: cdcl3
Temp. 26.0 C / 299.1 K
Operator: walkup
File: cdcl3_01
VNMRS-500 "NMR500"

Relax. delay 1.000 sec
Pulse 45.0 degrees
Acq. time 2.049 sec
Width 8012.8 Hz
8 repetitions
OBSERVE H1, 499.7316502 MHz
DATA PROCESSING
Resol. enhancement -0.0 Hz
FT size 65536
Total time 0 min, 31 sec

1-Phenoxycarbonyl-2-benzyl-3-methoxy-1,2-dihydropyrazine (3f)

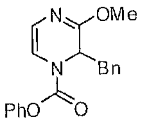

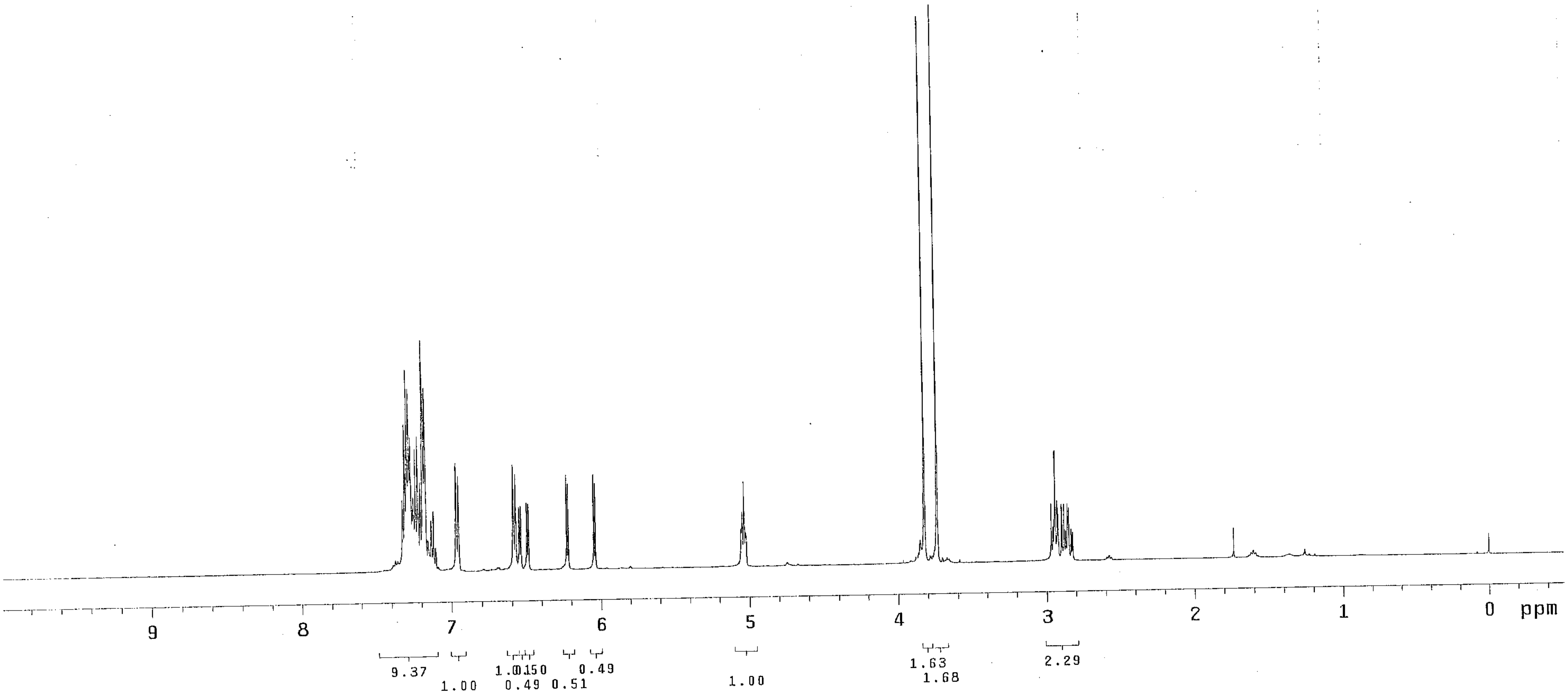

1-Phenoxycarbonyl-2-benzyl-3-methoxy-1,2-dihydropyrazine (3f)

1-Phenoxycarbonyl-2-benzyl-3

Sample: VRS-6-49B
Sample ID: s_20120611_11
File: s_20120611_11/data/cdc

Pulse Sequence: s2pul
Solvent: cdcl3
Temp. 26.0 C / 299.1 K
Operator: walkup
File: cdcl3_01
VNMRS-500 "NMR500"

Relax. delay 1.000 sec
Pulse 45.0 degrees
Acq. time 1.300 sec
Width 30487.8 Hz
256 repetitions
OBSERVE C13, 125.6576344 MHz
DECOUPLE H1, 499.7341220 MHz
Power 42 dB
continuously on
WALTZ-16 modulated
DATA PROCESSING
Line broadening 0.5 Hz
FT size 131072
Total time 9 min, 51 sec

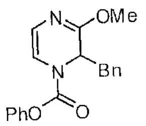

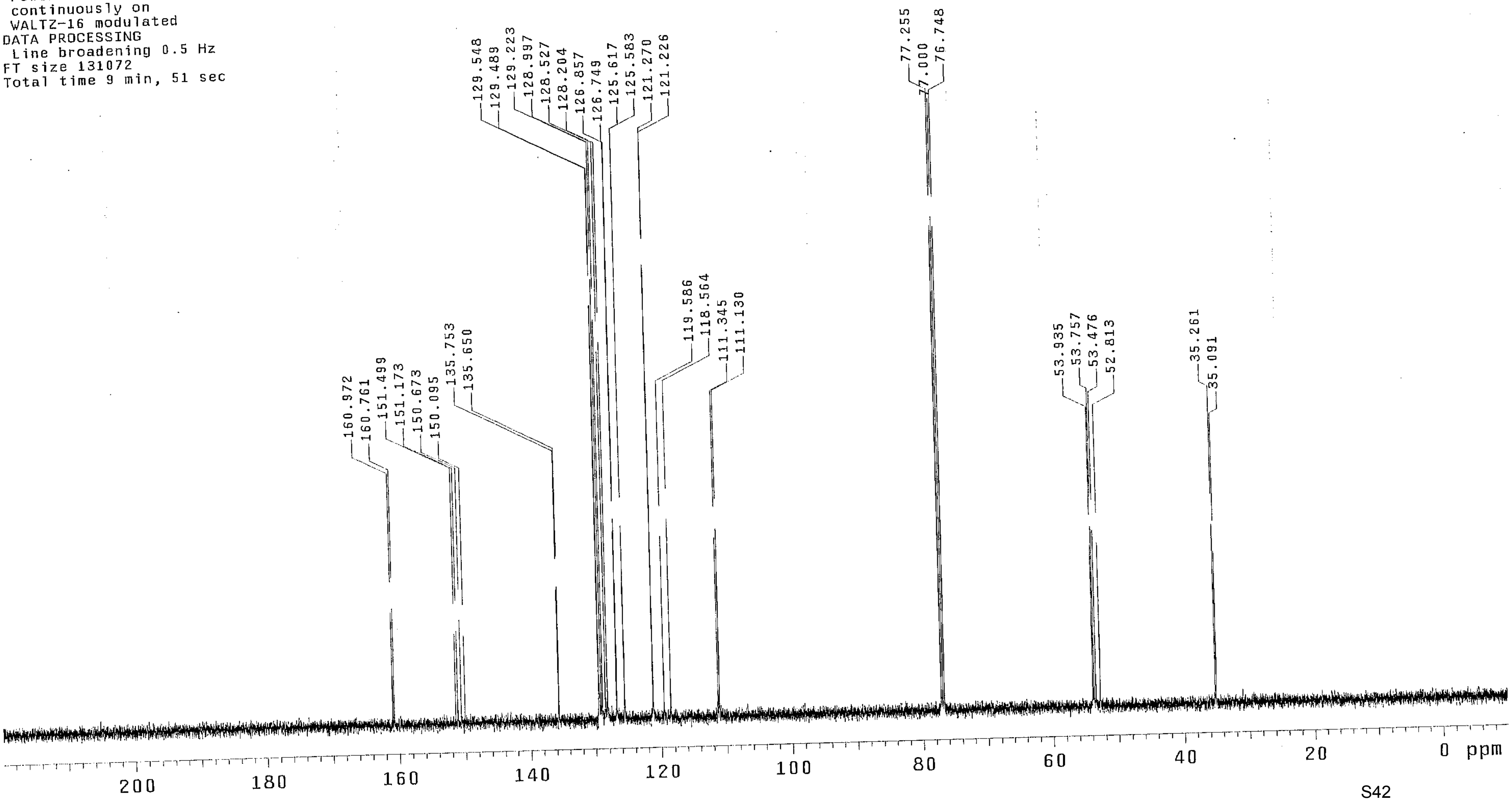

ReX'd

Sample: VRS-6-99B
Sample ID: s_20120823_01
File: s_20120823_01/data/cdcl3_01.

Pulse Sequence: s2pul

Solvent: cdcl3
Temp. 26.0 C / 299.1 K
Operator: walkup
File: cdcl3_01
VNMRS-500 "NMR500"

Relax. delay 1.000 sec
Pulse 45.0 degrees
Acq. time 2.049 sec
Width 8012.8 Hz
8 repetitions
OBSERVE H1, 499.7316336 MHz
DATA PROCESSING
Resol. enhancement -0.0 Hz
FT size 65536
Total time 0 min, 31 sec

1-Phenoxycarbonyl-2-(2-bromobenzyl)-3-methoxy-1,2-dihydropyrazine (3g)

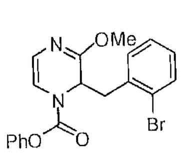

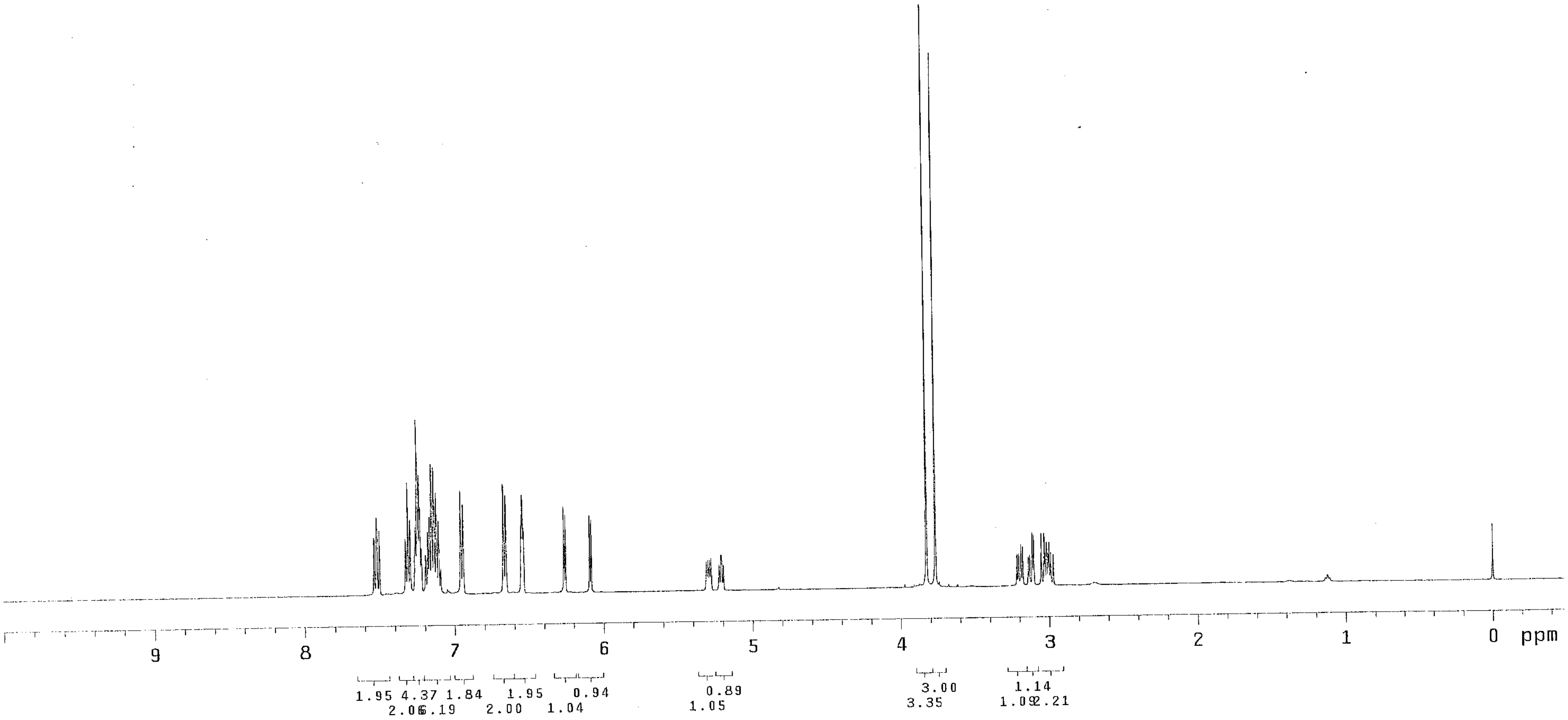

ReX'd

1-Phenoxycarbonyl-2-(2-bromobenzyl)-3-methoxy-1,2-dihydropyrazine (3g)

Sample: VRS-6-99B
Sample ID: s_20120823_02
File: s_20120823_02/data/cdc
Automation directory: /home/

Pulse Sequence: s2pul

Solvent: cdcl3
Temp. 26.0 C / 299.1 K
Operator: walkup
File: cdcl3_01
VNMRS-500 "NMR500"

Relax. delay 1.000 sec
Pulse 45.0 degrees
Acq. time 1.300 sec
Width 30487.8 Hz
256 repetitions
OBSERVE C13, 125.6576166 MHz
DECOUPLE H1, 499.7341220 MHz
Power 42 dB
continuously on
WALTZ-16 modulated
DATA PROCESSING
Line broadening 0.5 Hz
FT size 131072
Total time 9 min, 51 sec

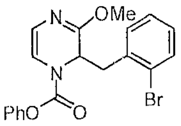

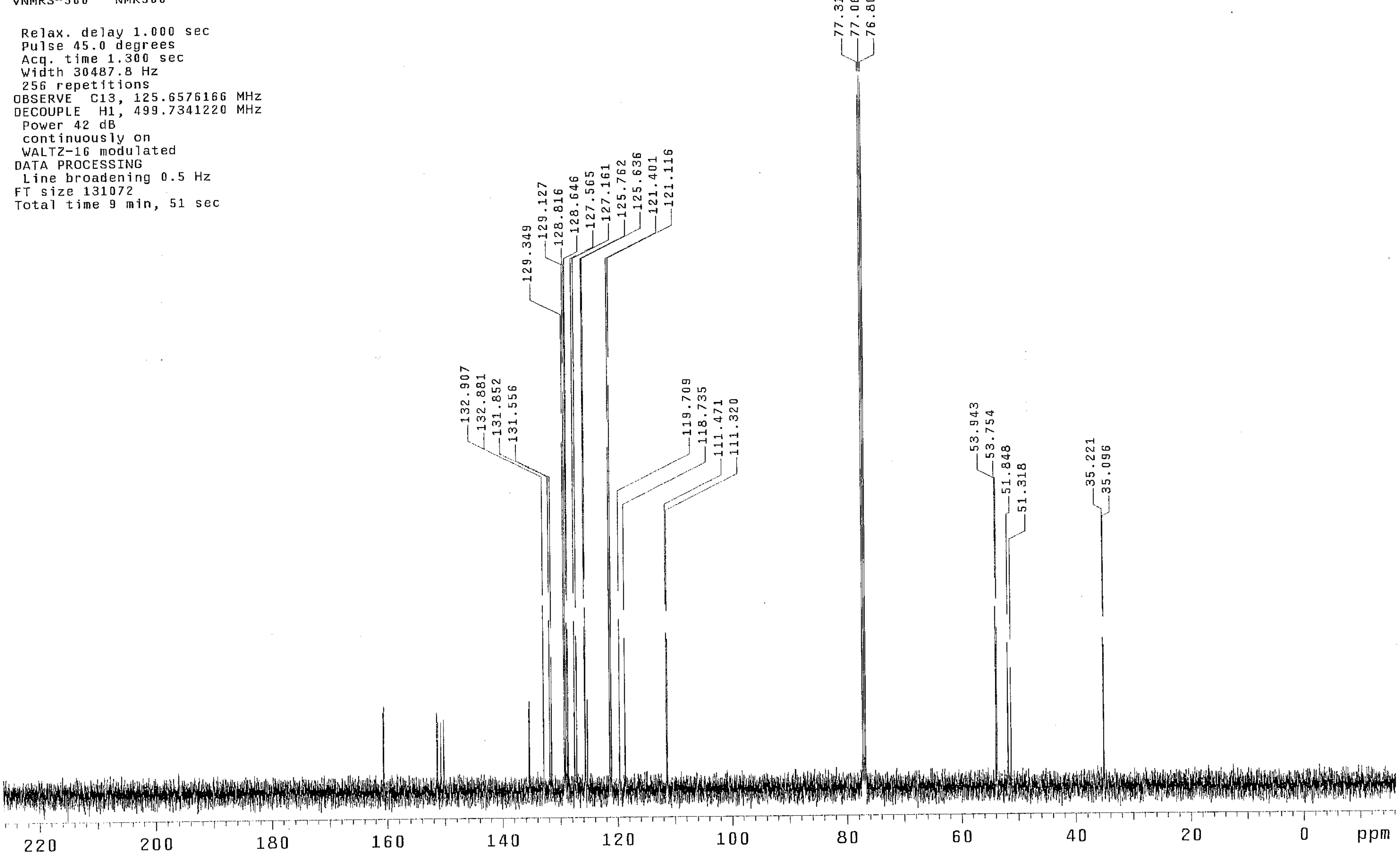

Target (ISCO)

Sample: VRS-6-48B
Sample ID: s_20140307_01
File: s_20140307_01/data/cdcl3_01.fid

Pulse Sequence: s2pul
Solvent: cdcl3
Ambient temperature
Operator: walkup
File: cdcl3_01
VNMRS-500 "NMR500"

Relax. delay 1.000 sec
Pulse 45.0 degrees
Acq. time 2.049 sec
Width 8012.8 Hz
8 repetitions
OBSERVE H1, 499.7316361 MHz
DATA PROCESSING
Resol. enhancement -0.0 Hz
FT size 65536
Total time 0 min, 31 sec

1-Phenoxycarbonyl-2-(4-chlorophenyl)-3-methoxy-1,2-dihydropyrazine (3h)

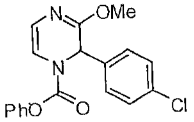

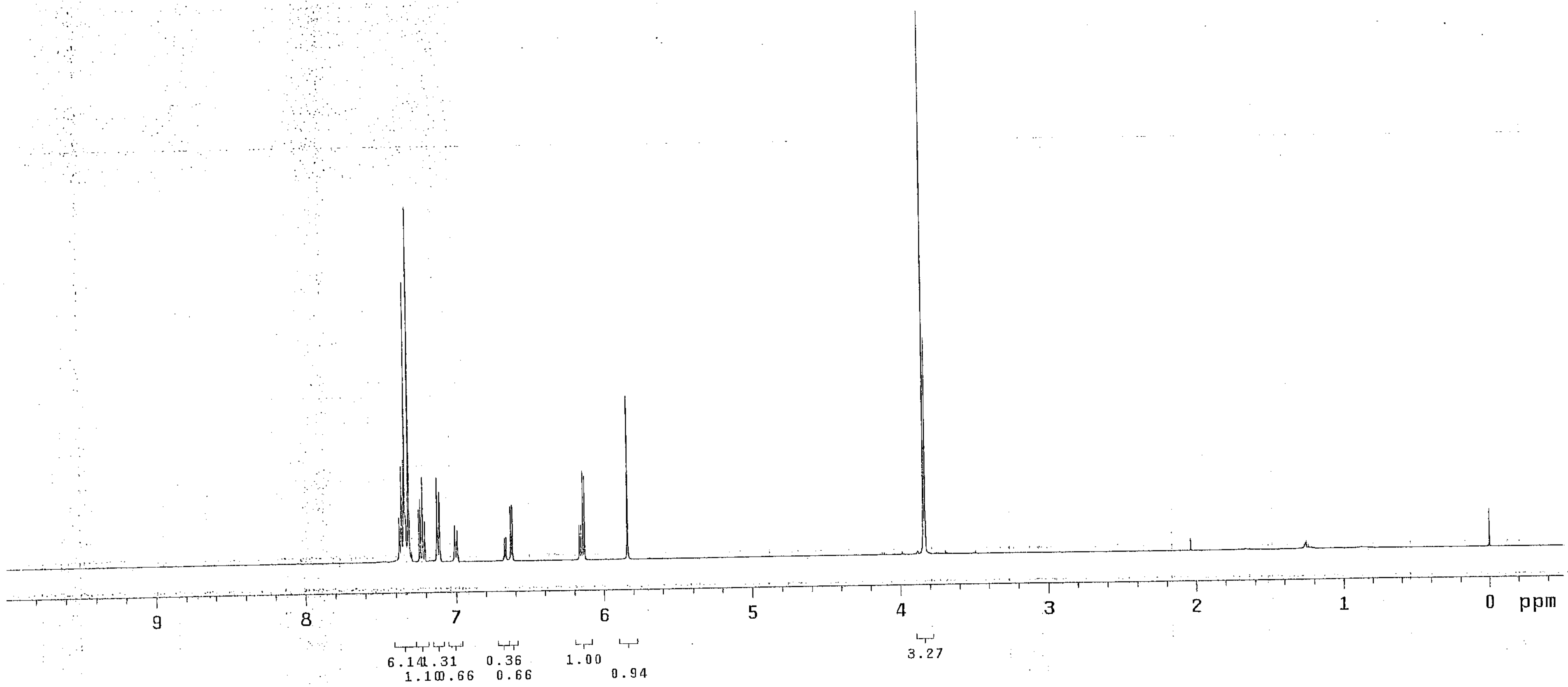

Target (ISCO)

Sample: VRS-6-48B
Sample ID: s_20140307_02
File: s_20140307_02/data/cdcl3_01.fid

Pulse Sequence: s2pul
Solvent: cdcl3
Ambient temperature
Operator: walkup
File: cdcl3_01
VNMRS-500 "NMR500"

Relax. delay 1.000 sec
Pulse 45.0 degrees
Acq. time 1.300 sec
Width 30487.8 Hz
256 repetitions
OBSERVE C13, 125.6576247 MHz
DECOUPLE H1, 499.7341220 MHz
Power 42 dB
continuously on
WALTZ-16 modulated
DATA PROCESSING
Line broadening 0.5 Hz
FT size 131072
Total time 9 min, 51 sec

1-Phenoxycarbonyl-2-(4-chlorophenyl)-3-methoxy-1,2-dihydropyrazine (3h)

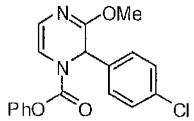

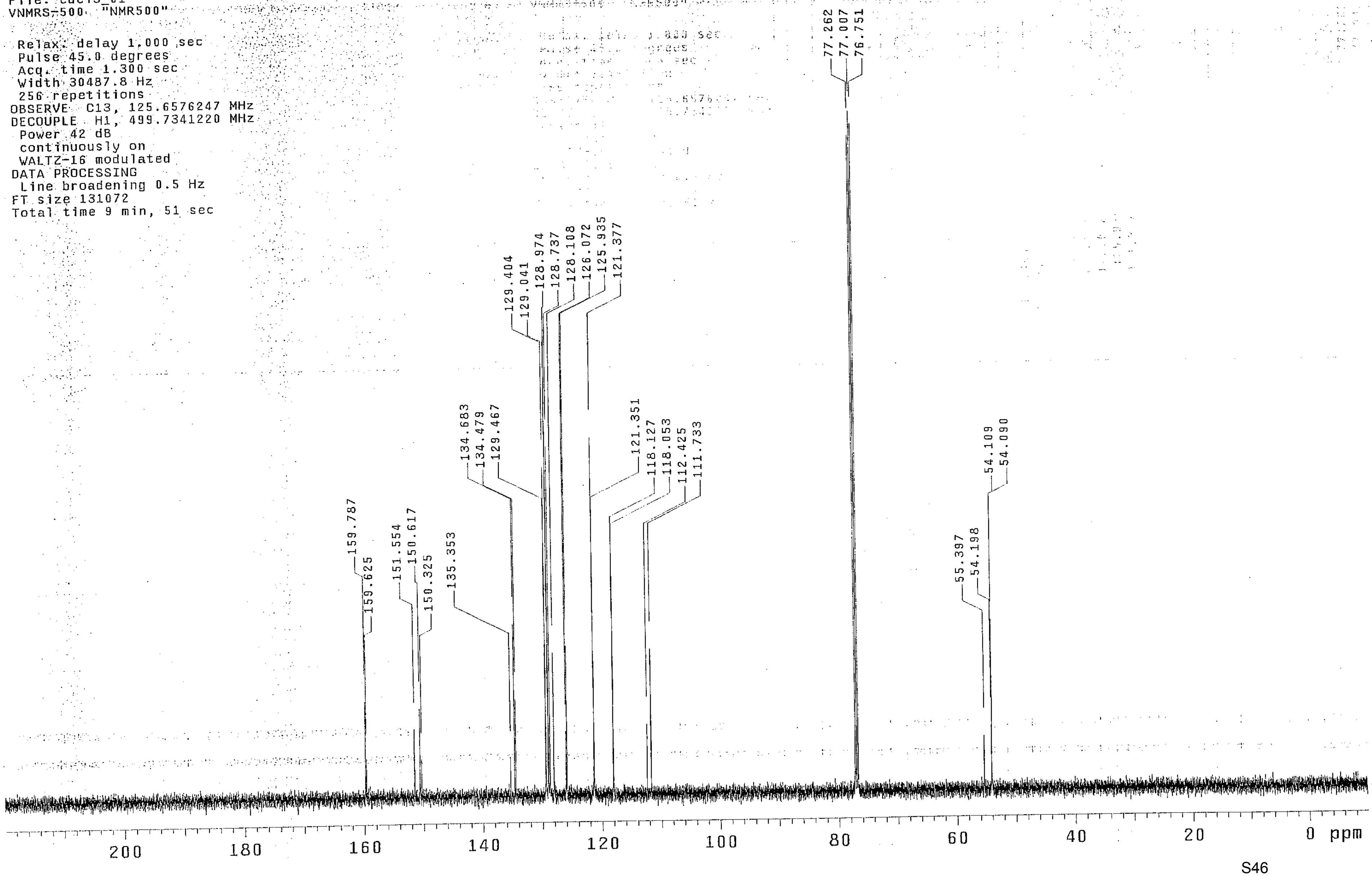

Purified

Sample: VRS-6-48A
Sample ID: s_20120613_02
File: s_20120613_02/data/cdcl3_01.fid

Pulse Sequence: s2pul

Solvent: cdcl3
Temp. 26.0 C / 299.1 K
Operator: walkup
File: cdcl3_01
VNMRS-500 "NMR500"

Relax. delay 1.000 sec
Pulse 45.0 degrees
Acq. time 2.049 sec
Width 8012.8 Hz
8 repetitions
OBSERVE H1, 499.7316493 MHz
DATA PROCESSING
Resol. enhancement -0.0 Hz
FT size 65536
Total time 0 min, 31 sec

1-Phenoxycarbonyl-2-(4-methoxyphenyl)-3-methoxy-1,2-dihydropyrazine (3i)

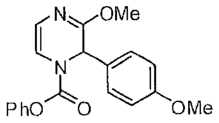

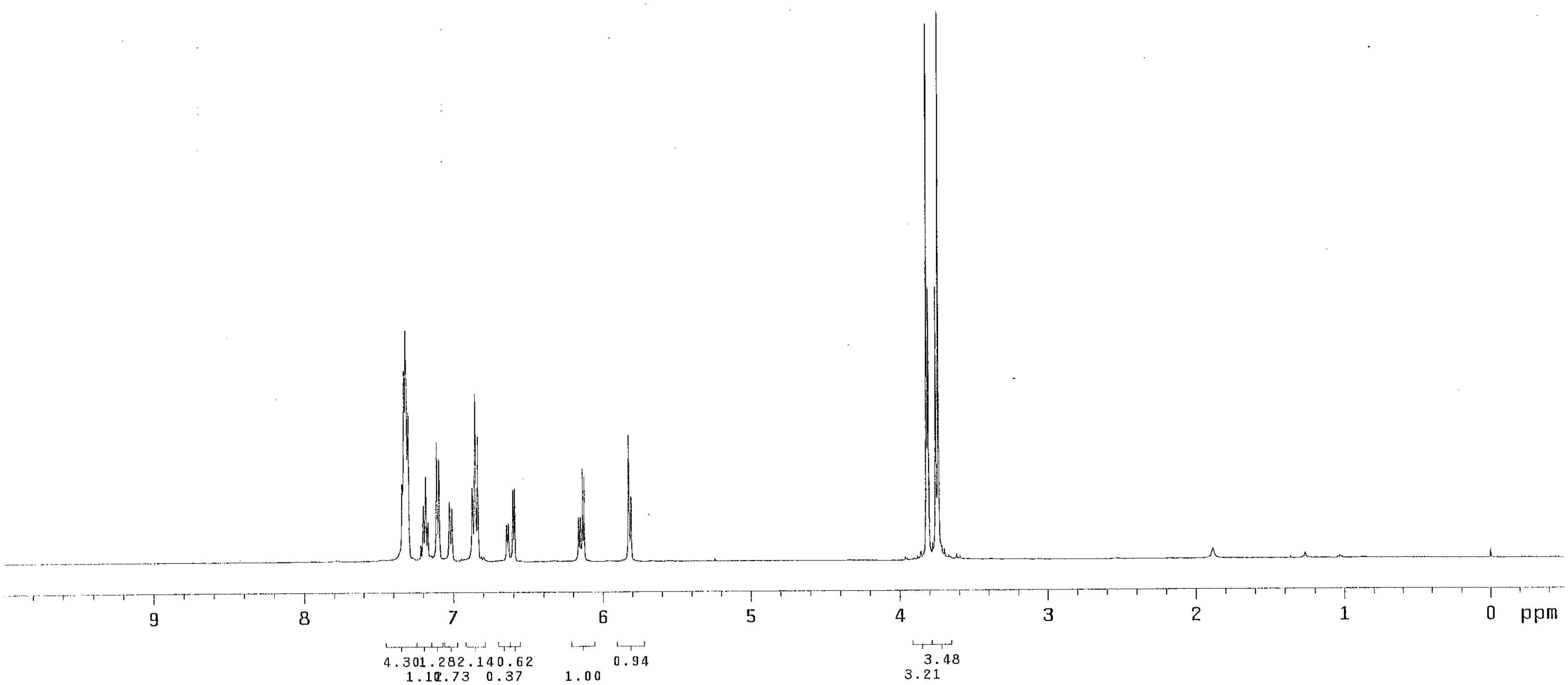

Purified

Sample: VRS-6-48A
Sample ID: s_20120613_03
File: s_20120613_03/data/cdcl3_01.fic

Pulse Sequence: s2pul
Solvent: cdcl3
Temp. 26.0 C / 299.1 K
Operator: walkup
File: cdcl3_01
VNMRS-500 "NMR500"

Relax. delay 1.000 sec
Pulse 45.0 degrees
Acq. time 1.300 sec
Width 30487.8 Hz
256 repetitions
OBSERVE C13, 125.6576166 MHz
DECOUPLE H1, 499.7341220 MHz
Power 42 dB
continuously on
WALTZ-16 modulated
DATA PROCESSING
Line broadening 0.5 Hz
FT size 131072
Total time 9 min, 51 sec

1-Phenoxycarbonyl-2-(4-methoxyphenyl)-3-methoxy-1,2-dihydropyrazine (3i)

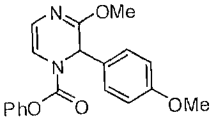

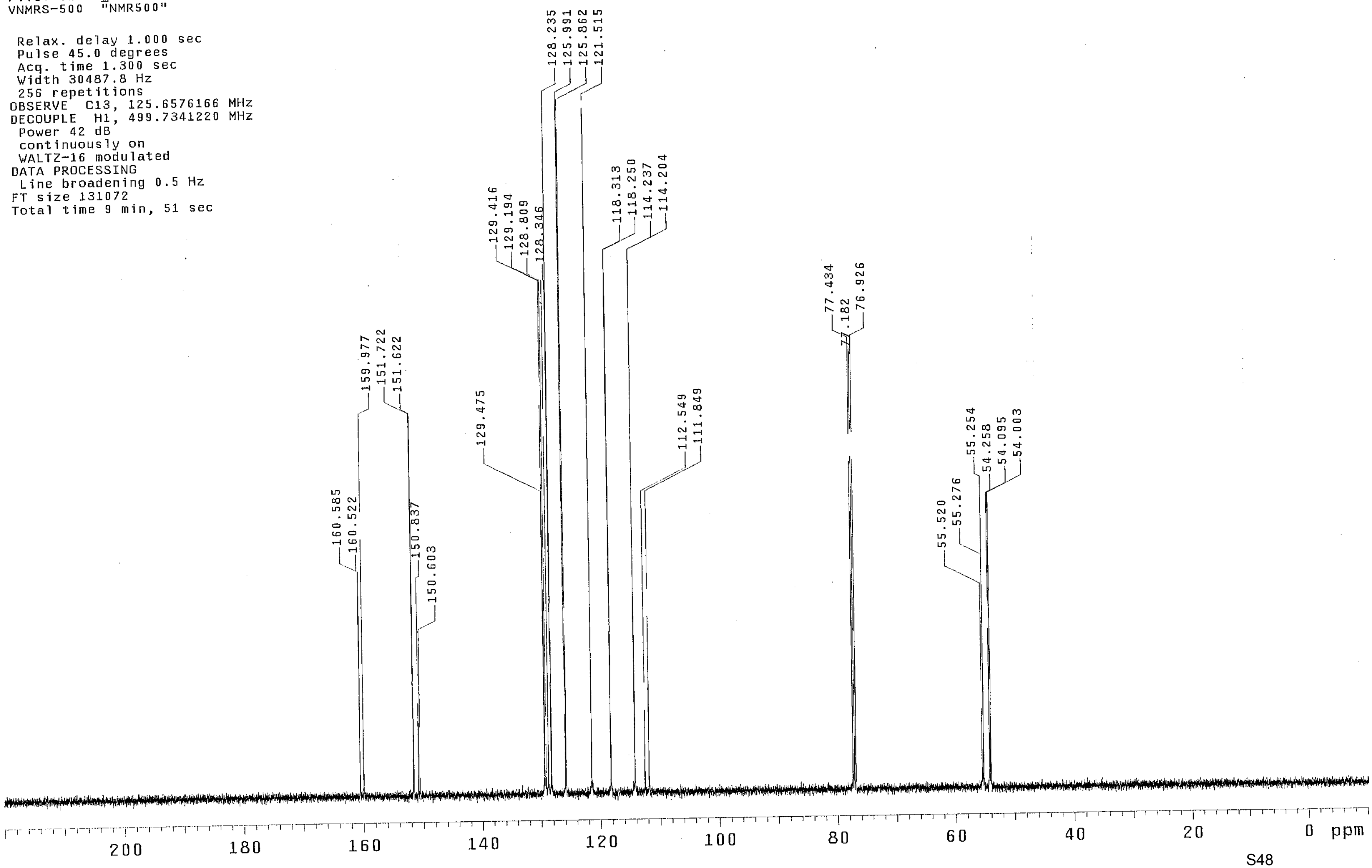

Target (ISCO)

Sample: VRS-6-49A
Sample ID: s_20140228_07
File: s_20140228_07/data/cdcl3_01.fi

Pulse Sequence: s2pul
Solvent: cdcl3
Ambient temperature
Operator: walkup
File: cdcl3_01
VNMRS-500 "NMR500"

Relax. delay 1.000 sec
Pulse 45.0 degrees
Acq. time 2.049 sec
Width 8012.8 Hz
8 repetitions
OBSERVE H1, 499.7316767 MHz
DATA PROCESSING
Resol. enhancement -0.0 Hz
FT size 65536
Total time 0 min, 31 sec

1-Phenoxycarbonyl-2-(2,4-dimethylphenyl)-3-methoxy-1,2-dihydropyrazine (3j)

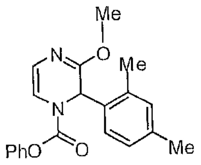

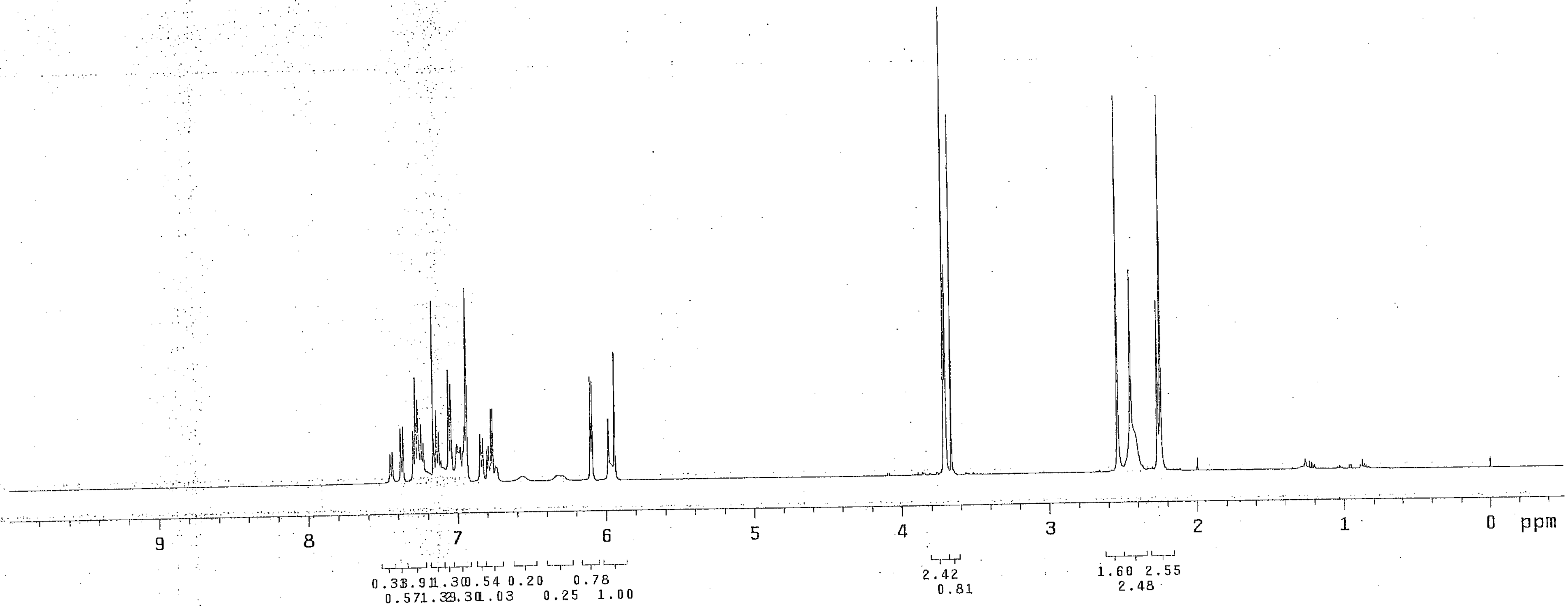

Target (ISCO)

Sample: VRS-6-49A
Sample ID: s_20140228_06
File: s_20140228_06/data/cdcl3_01.

Pulse Sequence: s2pul
Solvent: cdcl3
Ambient temperature
Operator: walkup
File: cdcl3_01
VNMRS-500 "NMR500"

Relax. delay 1.000 sec
Pulse 45.0 degrees
Acq. time 1.300 sec
Width 30487.8 Hz
256 repetitions
OBSERVE C13, 125.6576499 MHz
DECOUPLE H1, 499.7341220 MHz
Power 42 dB
continuously on
WALTZ-16 modulated
DATA PROCESSING
Line broadening 0.5 Hz
FT size 131072
Total time 9 min, 51 sec

1-Phenoxycarbonyl-2-(2,4-dimethylphenyl)-3-methoxy-1,2-dihydropyrazine (3j)

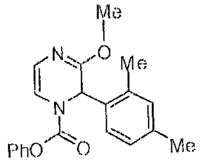

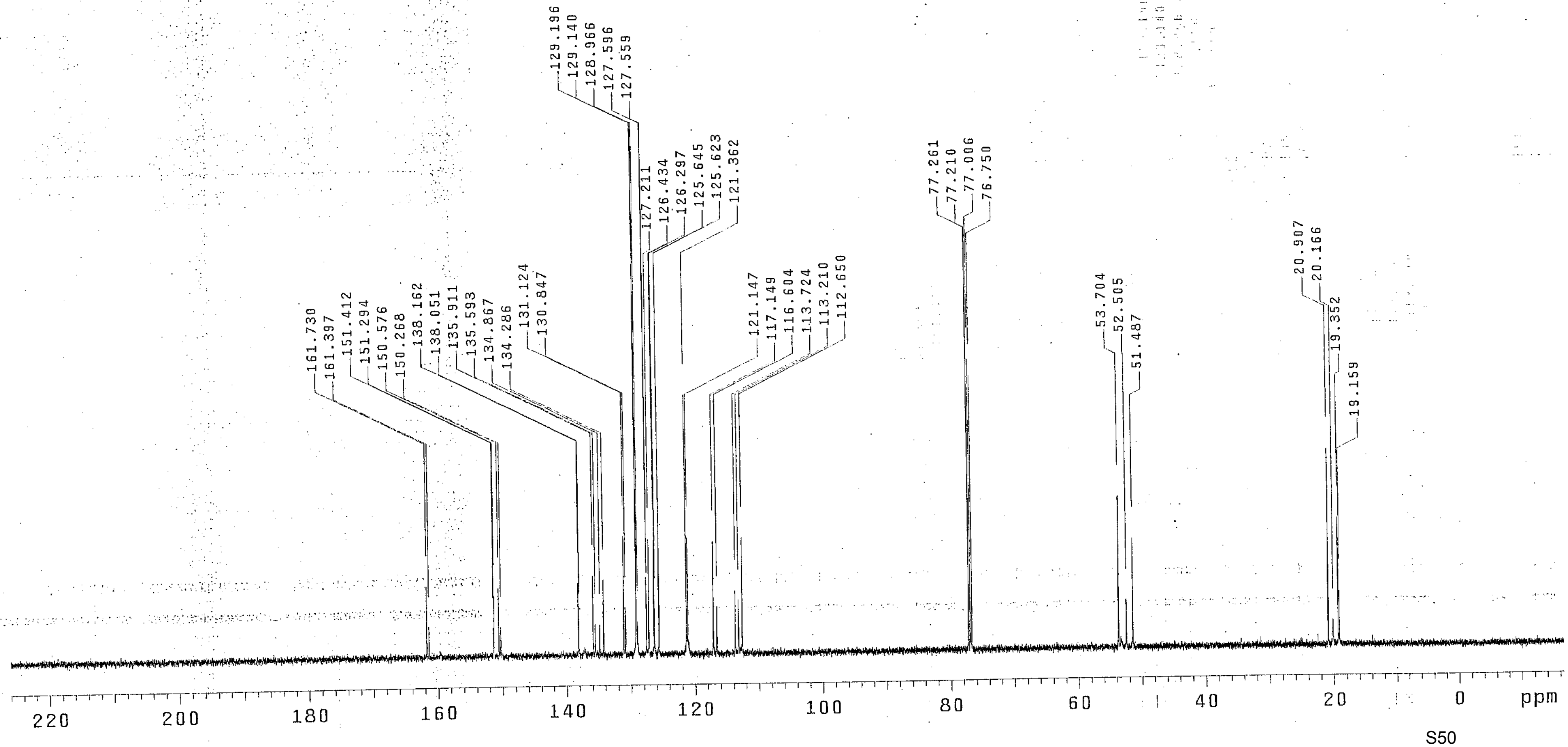

Purified

Sample: VRS-7-113A
Sample ID: s_20131121_01
File: /home/walkup/vnmrsys/data/auto_2012.02.02/s_20131121_01/data/cdcl3_01.fid

Pulse Sequence: s2pul
Solvent: cdcl3
Temp. 26.0 C / 299.1 K
Operator: walkup
File: cdcl3_01
VNMRS-500 "NMR500"

Relax. delay 1.000 sec
Pulse 45.0 degrees
Acq. time 2.049 sec
Width 8012.8 Hz
8 repetitions
OBSERVE H1, 499.7316356 MHz
DATA PROCESSING
Resol. enhancement -0.0 Hz
FT size 65536
Total time 0 min, 31 sec

1-Phenoxycarbonyl-2-methyl-3-benzyloxy-1,2-dihydropyrazine (4a).

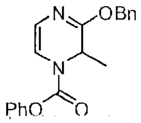

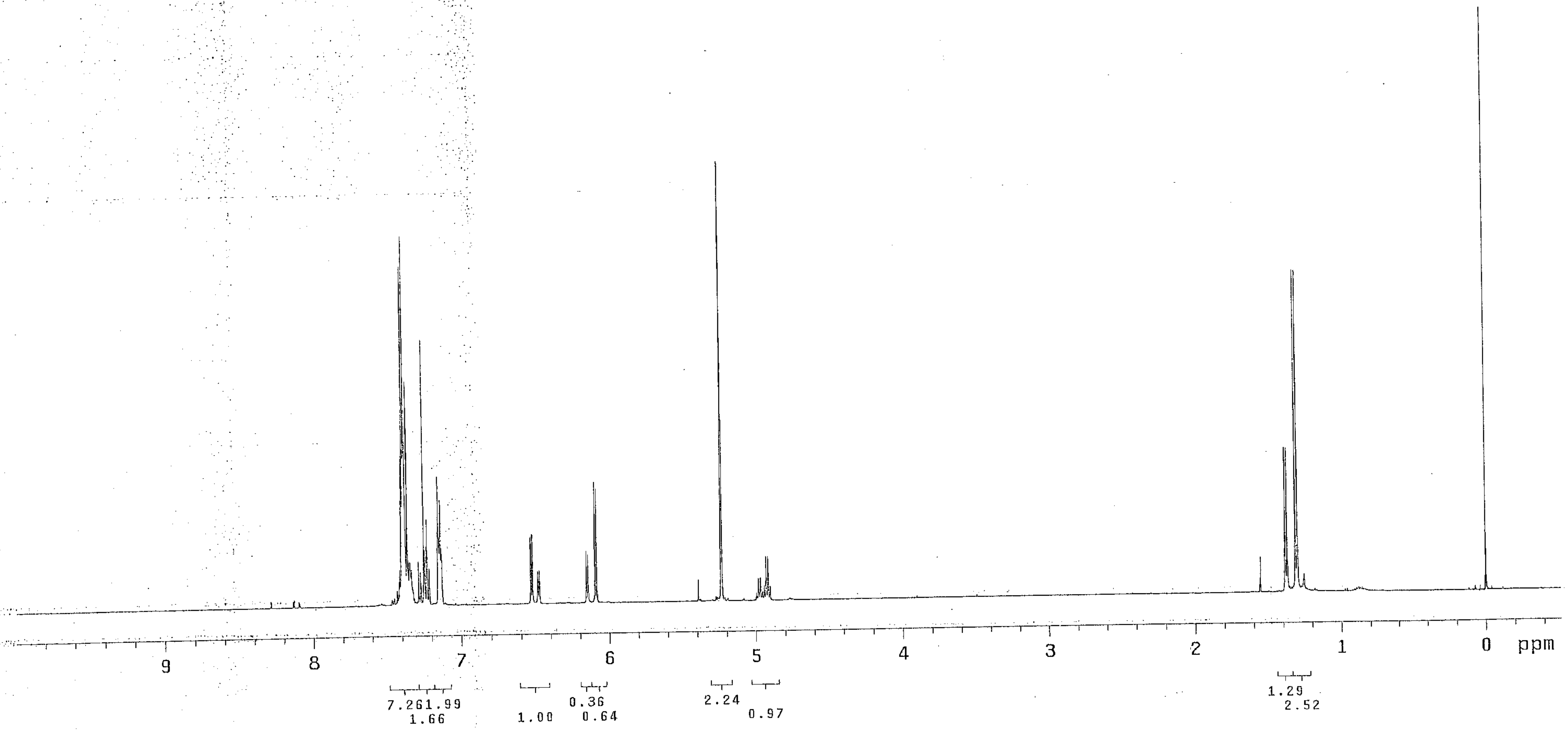

Target

Sample: VRS-7-113A
Sample ID: s_20140226_01
File: s_20140226_01/data/cdcl3_01.f

Pulse Sequence: s2pul
Solvent: cdcl3
Ambient temperature
Operator: walkup
File: cdcl3_01
VNMRS-500 "NMR500"

Relax. delay 1.000 sec
Pulse 45.0 degrees
Acq. time 1.300 sec
Width 30487.8 Hz
256 repetitions
OBSERVE C13, 125.6576318 MHz
DECOUPLE H1, 499.7341220 MHz
Power 42 dB
continuously on
WALTZ-16 modulated
DATA PROCESSING
Line broadening 0.5 Hz
FT size 131072
Total time 9 min, 51 sec

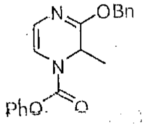

1-Phenoxycarbonyl-2-methyl-3-benzyloxy-1,2-dihydropyrazine (4a).

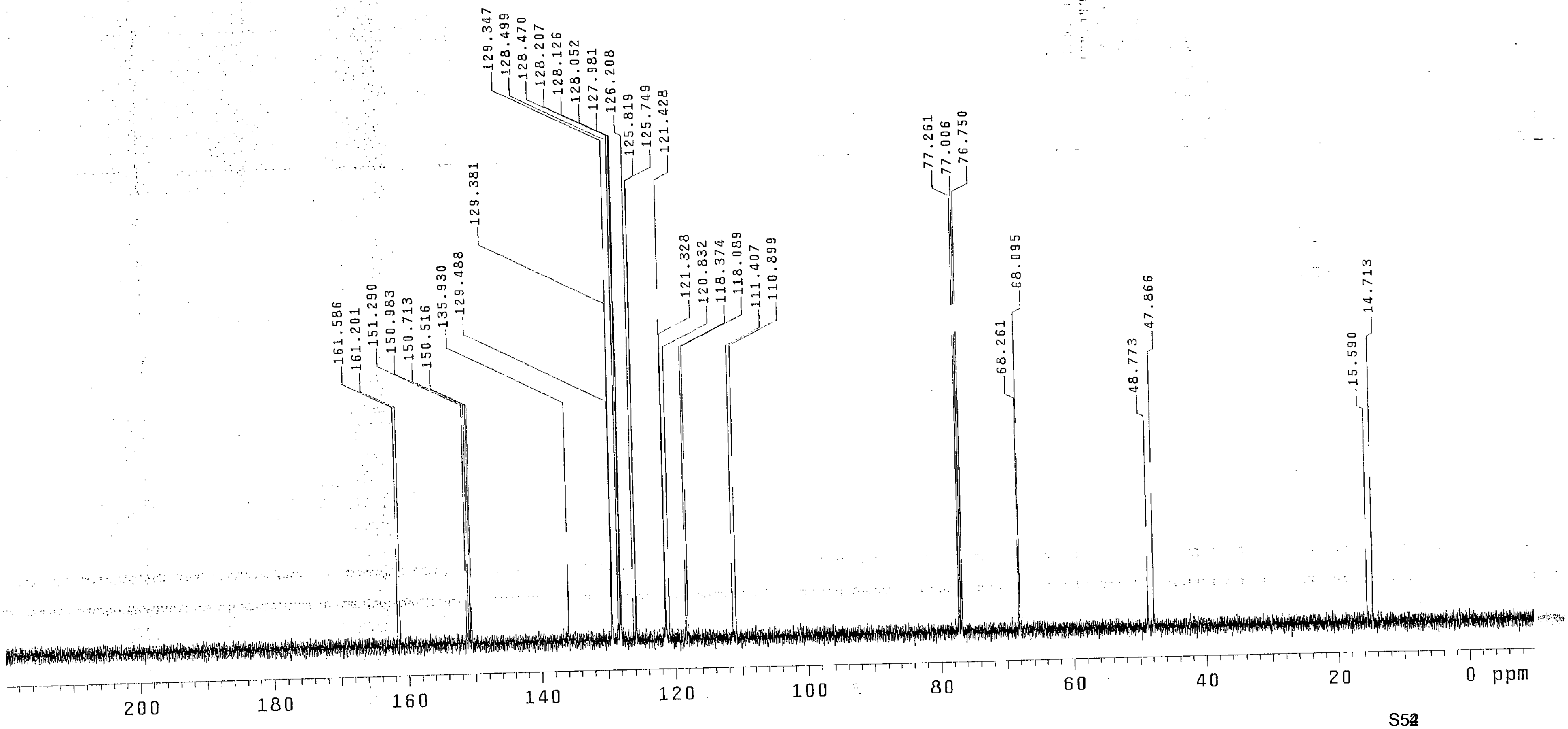

Purified

Sample: VRS-4-98
Sample ID: s_20140212_02
File: s_20140212_02/data/cdcl3_01.fid

Pulse Sequence: s2pul
Solvent: cdcl3
Temp. 26.0 C / 299.1 K
Operator: walkup
File: cdcl3_01
VNMRS-500 "NMR500"

Relax. delay 1.000 sec
Pulse 45.0 degrees
Acq. time 2.049 sec
Width 8012.8 Hz
8 repetitions
OBSERVE H1, 499.7316410 MHz
DATA PROCESSING
Resol. enhancement -0.0 Hz
FT size 65536
Total time 0 min, 31 sec

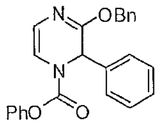

1-Phenoxycarbonyl-2-phenyl-3-benzyloxy-1,2-dihydropyrazine (4b)

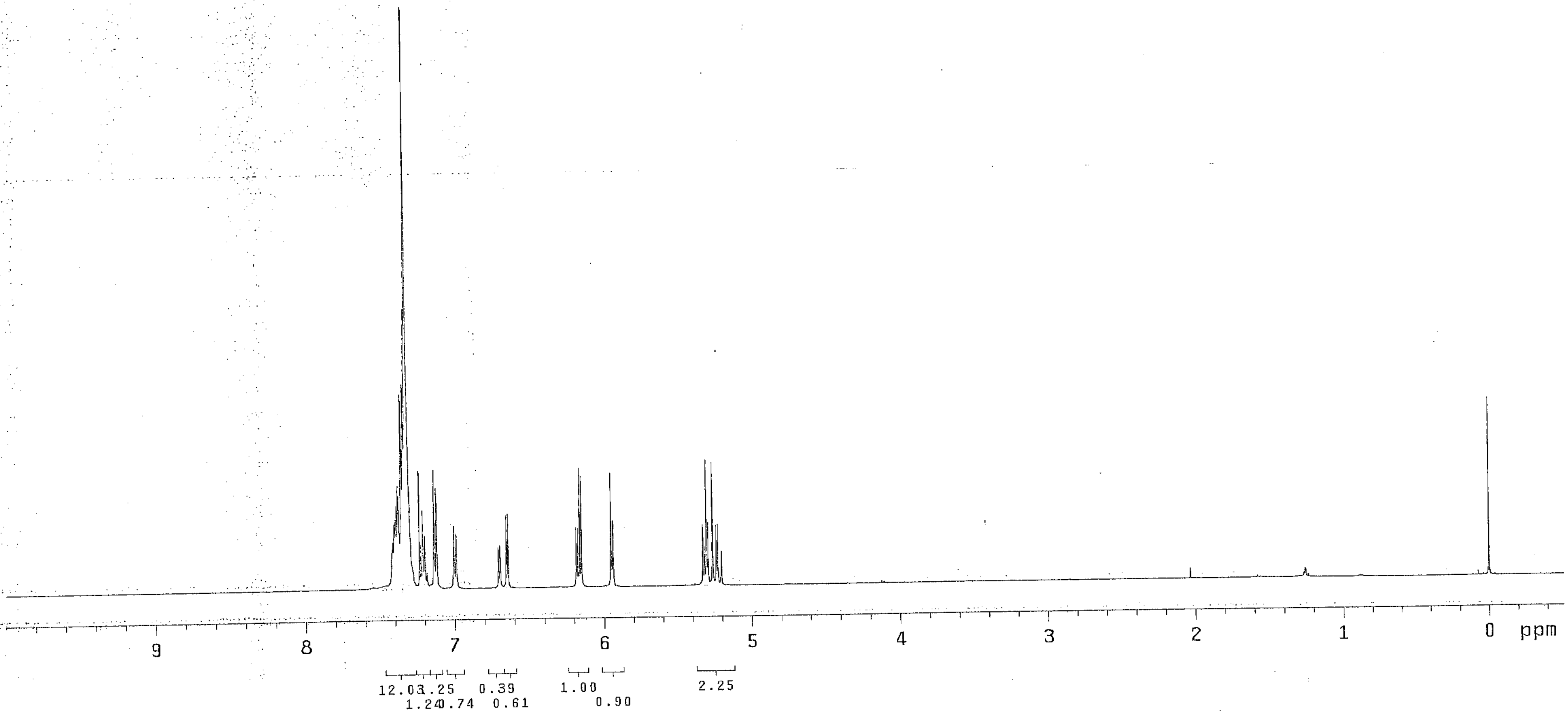

Purified

Sample: VRS-4-98
Sample ID: s_20140211_08
File: s_20140211_08/data/cdcl3_01.fid

Pulse Sequence: s2pul

Solvent: cdcl3
Temp. 26.0 C / 299.1 K
Operator: walkup
File: cdcl3_01
VNMRS-500 "NMR500"

Relax. delay 1.000 sec
Pulse 45.0 degrees
Acq. time 1.300 sec
Width 30487.8 Hz
256 repetitions
OBSERVE C13, 125.6576166 MHz
DECOUPLE H1, 499.7341220 MHz
Power 42 dB
continuously on
WALTZ-16 modulated
DATA PROCESSING
Line broadening 0.5 Hz
FT size 131072
Total time 9 min, 51 sec

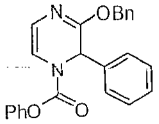

1-Phenoxycarbonyl-2-phenyl-3-benzyloxy-1,2-dihydropyrazine (4b)

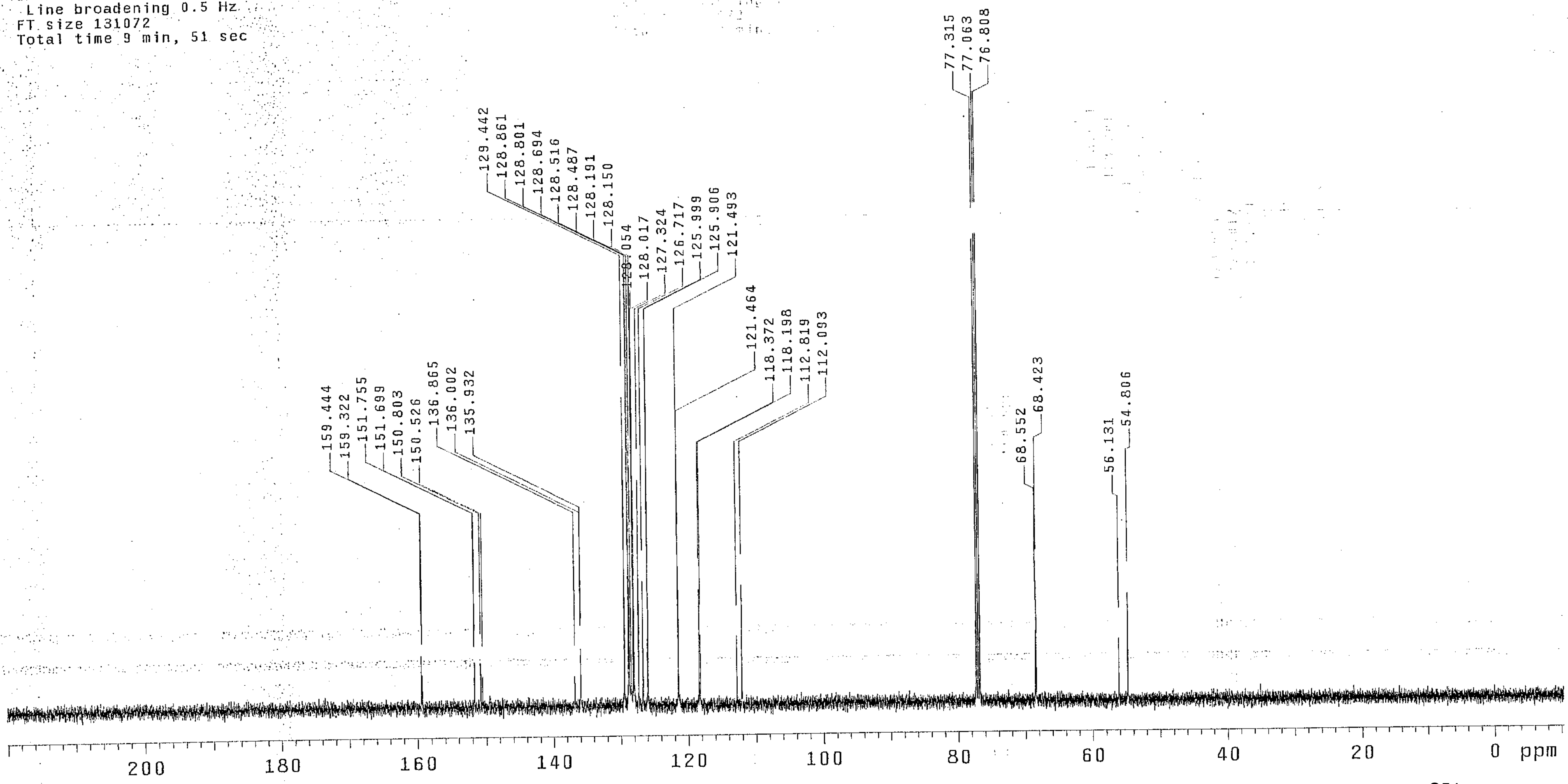

Purified

Sample: WH-1-28 (2)
Sample ID: s_20121005_02
File: /home/walkup/vnmrsys/(

Pulse Sequence: s2pul
Solvent: cdcl3
Temp. 26.0 C / 299.1 K
Operator: walkup
File: cdcl3_01
VNMRS-500 "NMR500"

Relax. delay 1.000 sec
Pulse 45.0 degrees
Acq. time 2.049 sec
Width 8012.8 Hz
8 repetitions
OBSERVE H1, 499.7316370 MHz
DATA PROCESSING
Resol. enhancement -0.0 Hz
FT size 65536
Total time 0 min, 31 sec

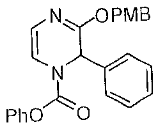

1-Phenoxycarbonyl-2-phenyl-3-(4-methoxybenzyloxy)-1,2-dihydropyrazine (5)

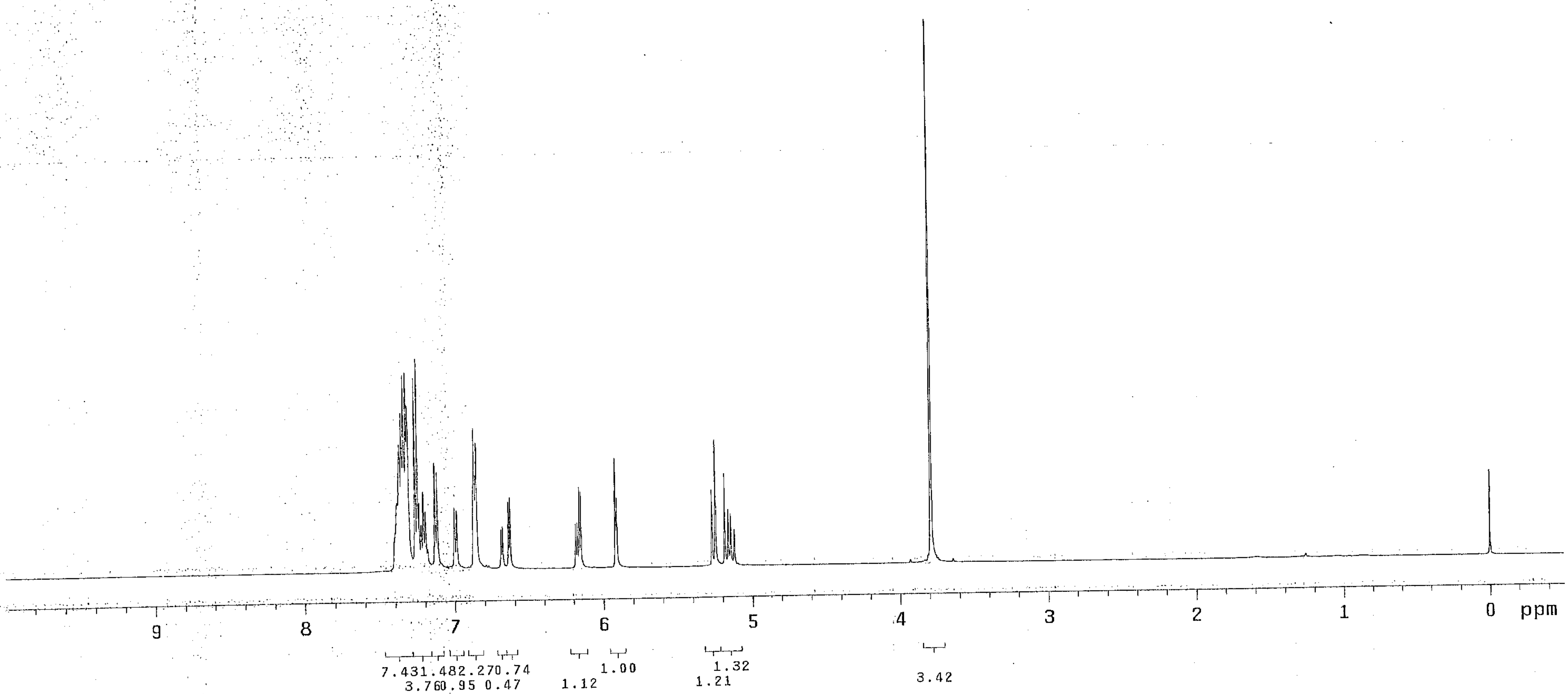

Purified

Sample: WH-1-28 (2)
Sample ID: s_20121003_07
File: /home/walkup/Val-NMRs/WH-1-28-

Pulse Sequence: s2pul
Solvent: cdcl3
Temp. 26.0 C / 299.1 K
Operator: walkup
File: WH-1-28-2
VNMRS-500 "NMR500"

Relax. delay 1.000 sec
Pulse 45.0 degrees
Acq. time 1.300 sec
Width 30487.8 Hz
7360 repetitions
OBSERVE C13, 125.6576274 MHz
DECOUPLE H1, 499.7341220 MHz
Power 42 dB
continuously on
WALTZ-16 modulated
DATA PROCESSING
Line broadening 0.5 Hz
FT size 131072
Total time 16 hr, 25 min, 21 sec

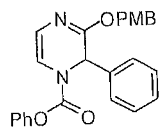

1-Phenoxycarbonyl-2-phenyl-3-(4-methoxybenzyloxy)-1,2-dihydropyrazine (5)

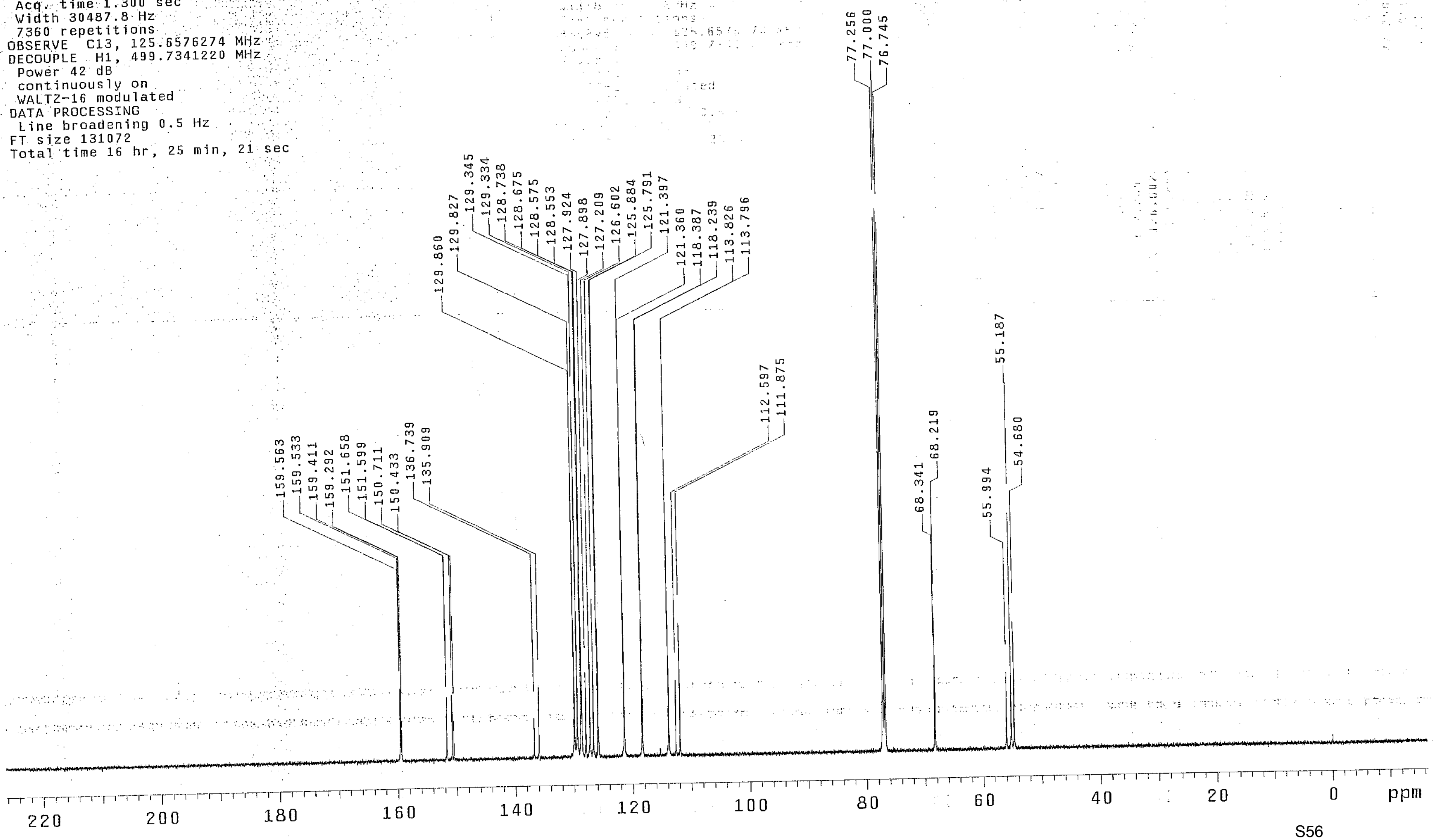

Purify

Sample: VRS-6-123a
Sample ID: s_20121025_06
File: /home/walkup/vnmrsys/data/au

Pulse Sequence: s2pul

Solvent: cdcl3
Temp. 26.0 C / 299.1 K
Operator: walkup
File: cdcl3_01
VNMRS-500 "NMR500"

Relax. delay 1.000 sec
Pulse 45.0 degrees
Acq. time 2.049 sec
Width 8012.8 Hz
8 repetitions
OBSERVE H1, 499.7316436 MHz
DATA PROCESSING
Resol. enhancement -0.0 Hz
FT size 65536
Total time 0 min, 31 sec

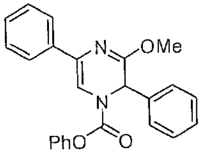

1-Phenoxycarbonyl-2,5-diphenyl-3-methoxy-1,2-dihydropyrazine (6)

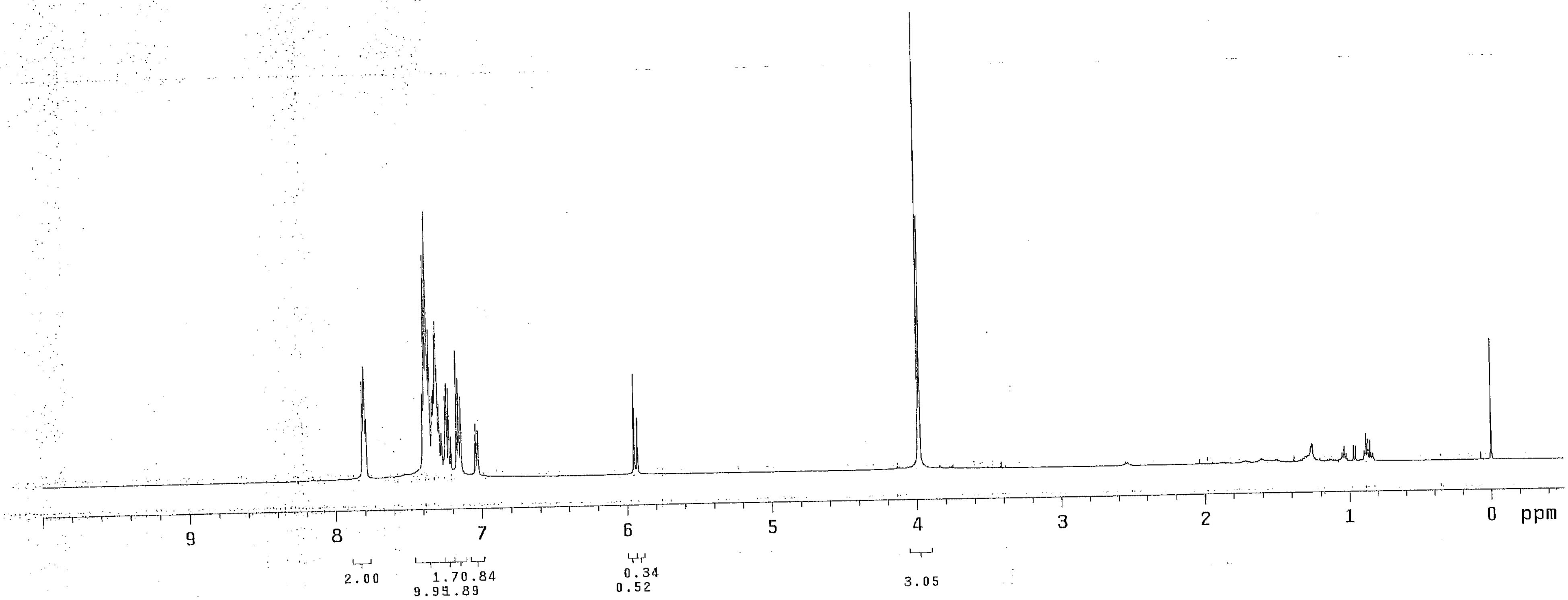

Target

Sample: VRS-6-123A
Sample ID: s_20140217_04
File: s_20140217_04/data/cdcl3_01.fid

Pulse Sequence: s2pul
Solvent: cdcl3
Temp. 26.0 C / 299.1 K
Operator: walkup
File: cdcl3_01
VNMRS-500 "NMR500"

Relax. delay 1.000 sec
Pulse 45.0 degrees
Acq. time 1.300 sec
Width 30487.8 Hz
256 repetitions
OBSERVE C13, 125.6576533 MHz
DECOUPLE H1, 499.7341220 MHz
Power 42 dB
continuously on
WALTZ-16 modulated
DATA PROCESSING
Line broadening 0.5 Hz
FT size 131072
Total time 9 min, 51 sec

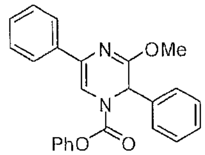

1-Phenoxycarbonyl-2,5-diphenyl-3-methoxy-1,2-dihydropyrazine (6)

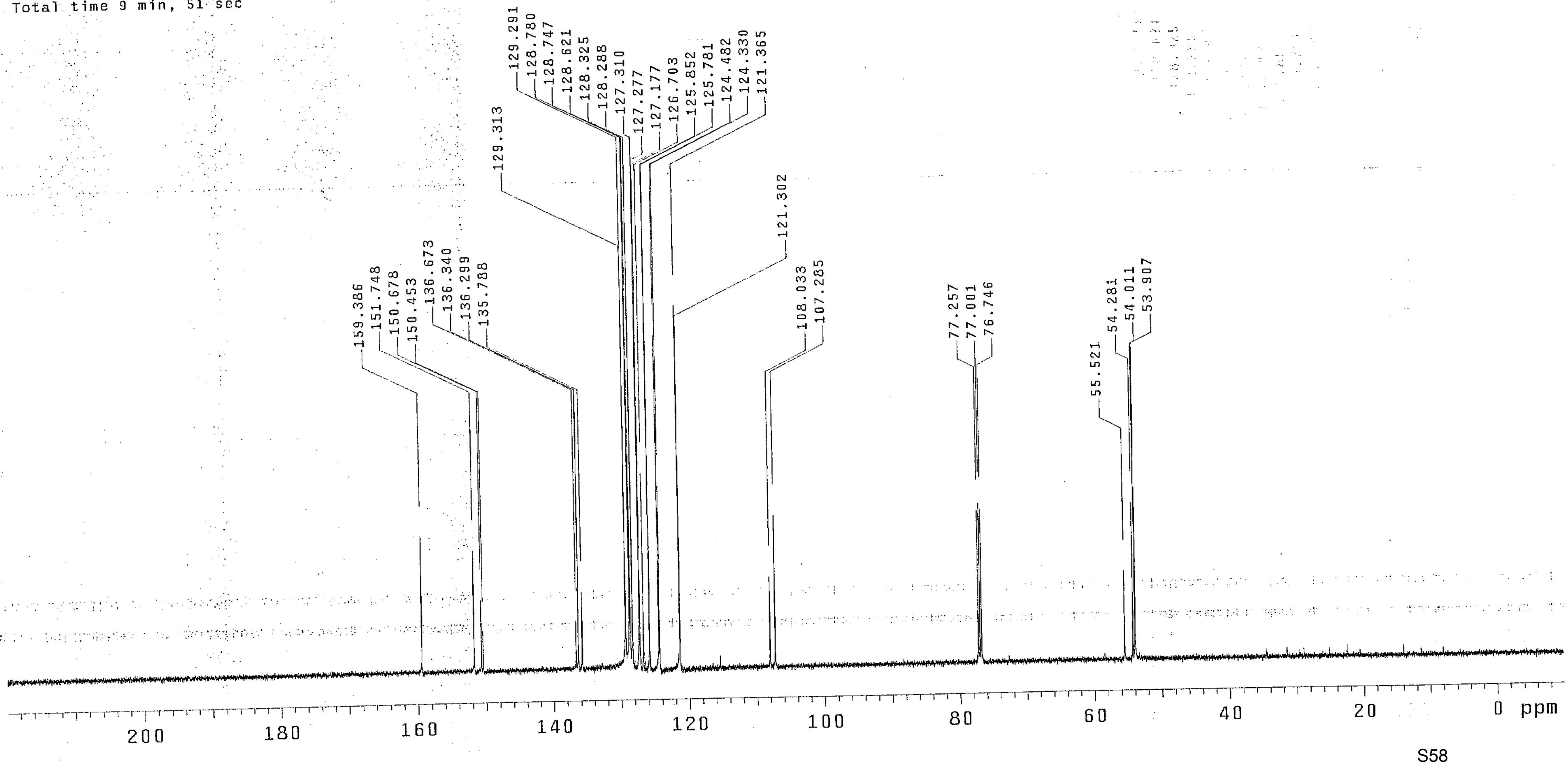

Target for ID

Sample: VRS-6-126A
Sample ID: s_20140220_01
File: s_20140220_01/data/cd

Pulse Sequence: s2pul
Solvent: cdcl3
Ambient temperature
Operator: walkup
File: cdcl3_01
VNMRS-500 "NMR500"

Relax. delay 1.000 sec
Pulse 45.0 degrees
Acq. time 2.049 sec
Width 8012.8 Hz
8 repetitions
OBSERVE H1, 499.7316314 MHz
DATA PROCESSING
Resol. enhancement -0.0 Hz
FT size 65536
Total time 0 min, 31 sec

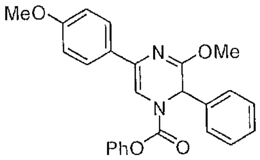

1-Phenoxycarbonyl-2-phenyl-3-methoxy-5-(4-methoxyphenyl)-1,2-dihydropyrazine (7a)

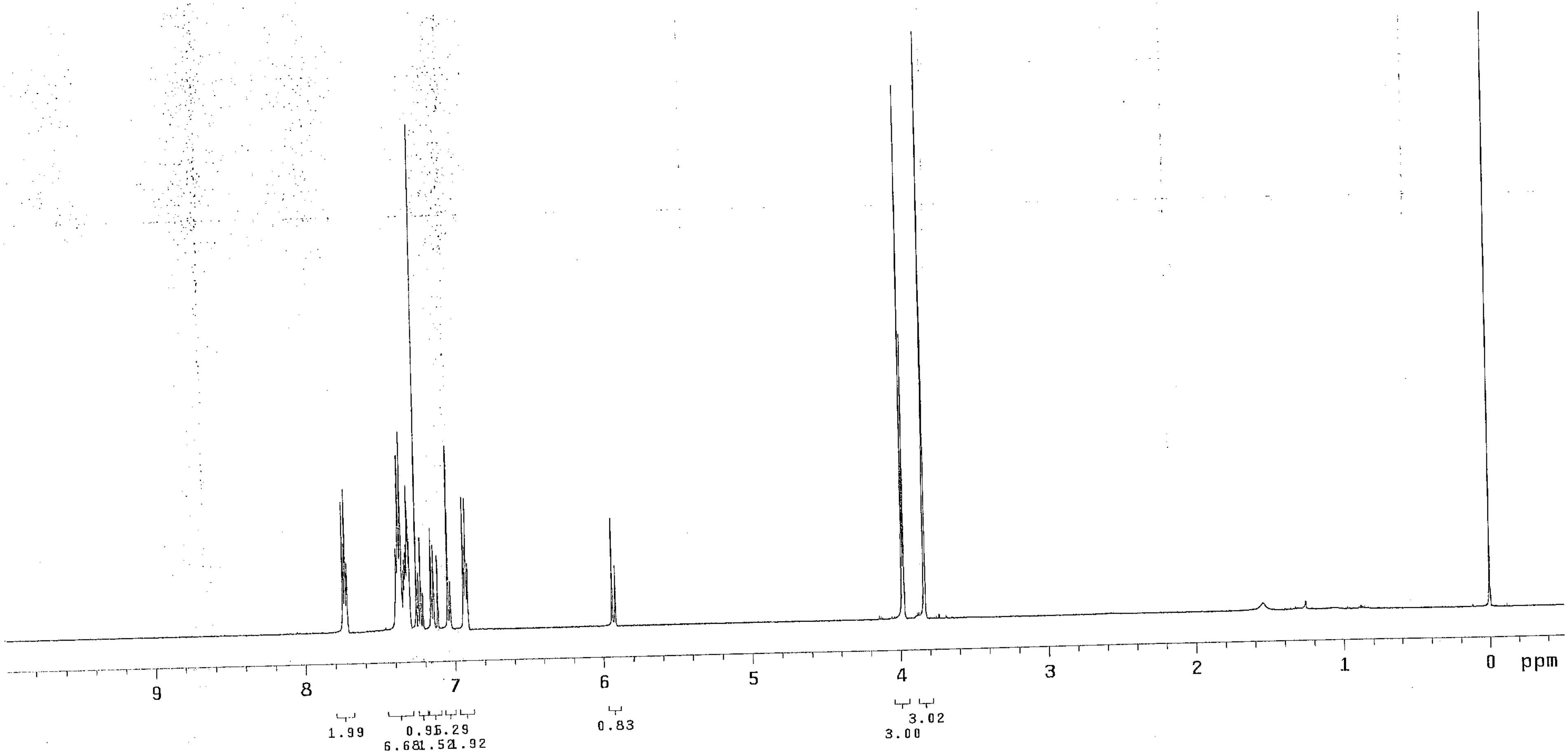

1-Phenoxycarbonyl-2-phenyl-3-methoxy-5-(4-methoxyphenyl)-1,2-dihydropyrazine (7a)

Target

Sample: VRS-6-126A
File: exp

Pulse Sequence: s2pul
Solvent: cdcl3
Ambient temperature
Operator: walkup
VNMRS-500 "NMR500"

Relax. delay 1.000 sec
Pulse 45.0 degrees
Acq. time 1.300 sec
Width 30487.8 Hz
1856 repetitions
OBSERVE C13, 125.6576166 MHz
DECOUPLE H1, 499.7341220 MHz
Power 42 dB
continuously on
WALTZ-16 modulated
DATA PROCESSING
Line broadening 0.5 Hz
FT size 131072
Total time 16 hr, 25 min, 21 sec

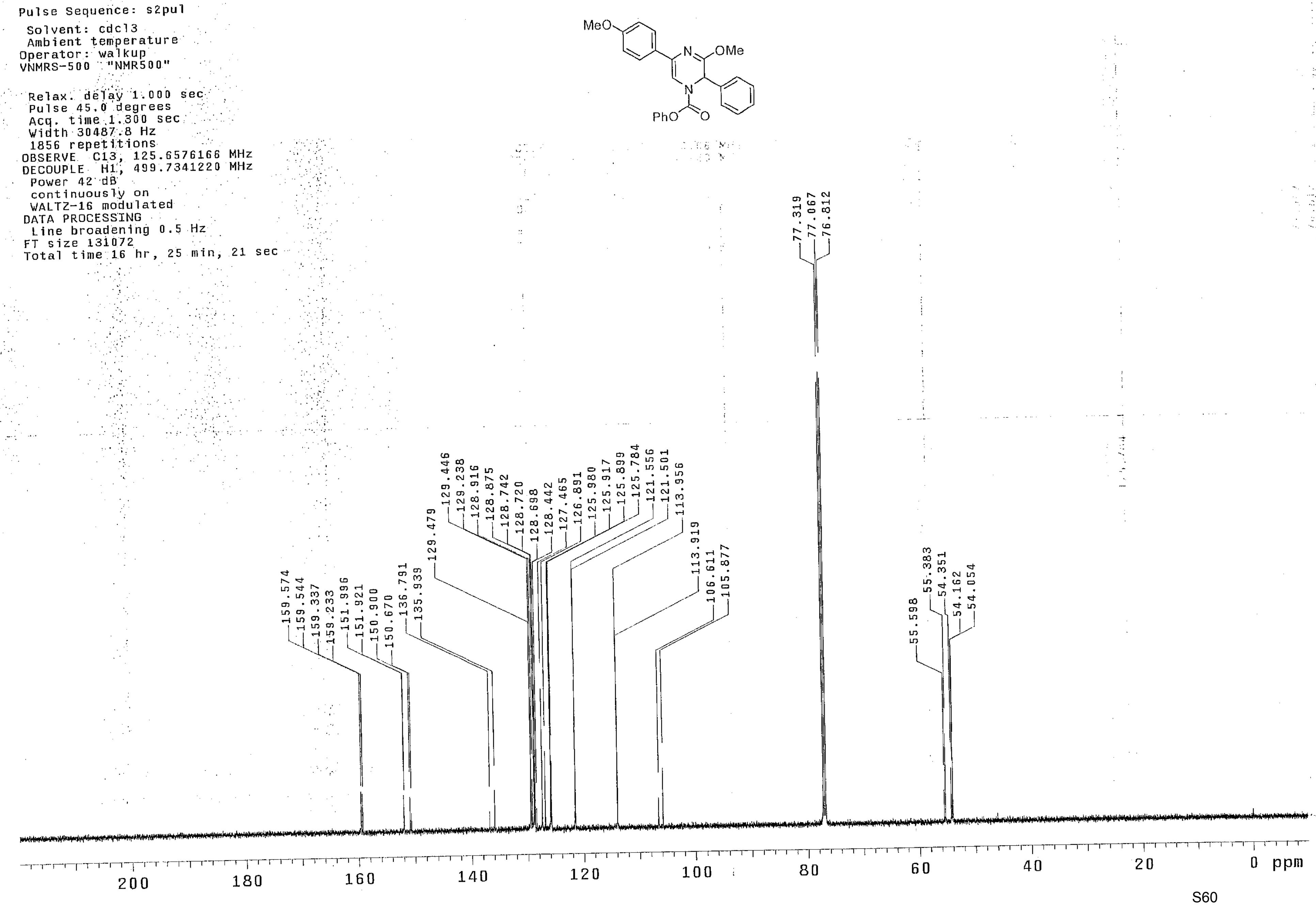

Purified

Sample: VRS-7-125B
Sample ID: s_20140531_01
File: s_20140531_01/data/cdcl3_0

Pulse Sequence: s2pul
Solvent: cdcl3
Temp. 26.0 C / 299.1 K
Operator: walkup
File: cdcl3_01
VNMRS-500 "NMR500"

Relax. delay 1.000 sec
Pulse 45.0 degrees
Acq. time 2.049 sec
Width 8012.8 Hz
8 repetitions
OBSERVE H1, 499.7316334 MHz
DATA PROCESSING
Resol. enhancement -0.0 Hz
FT size 65536
Total time 0 min, 31 sec

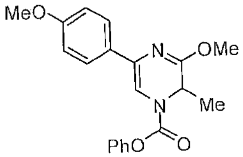

1-Phenoxycarbonyl-2-methyl-3-methoxy-5-(4-methoxyphenyl)-1,2-dihydropyrazine (7b)

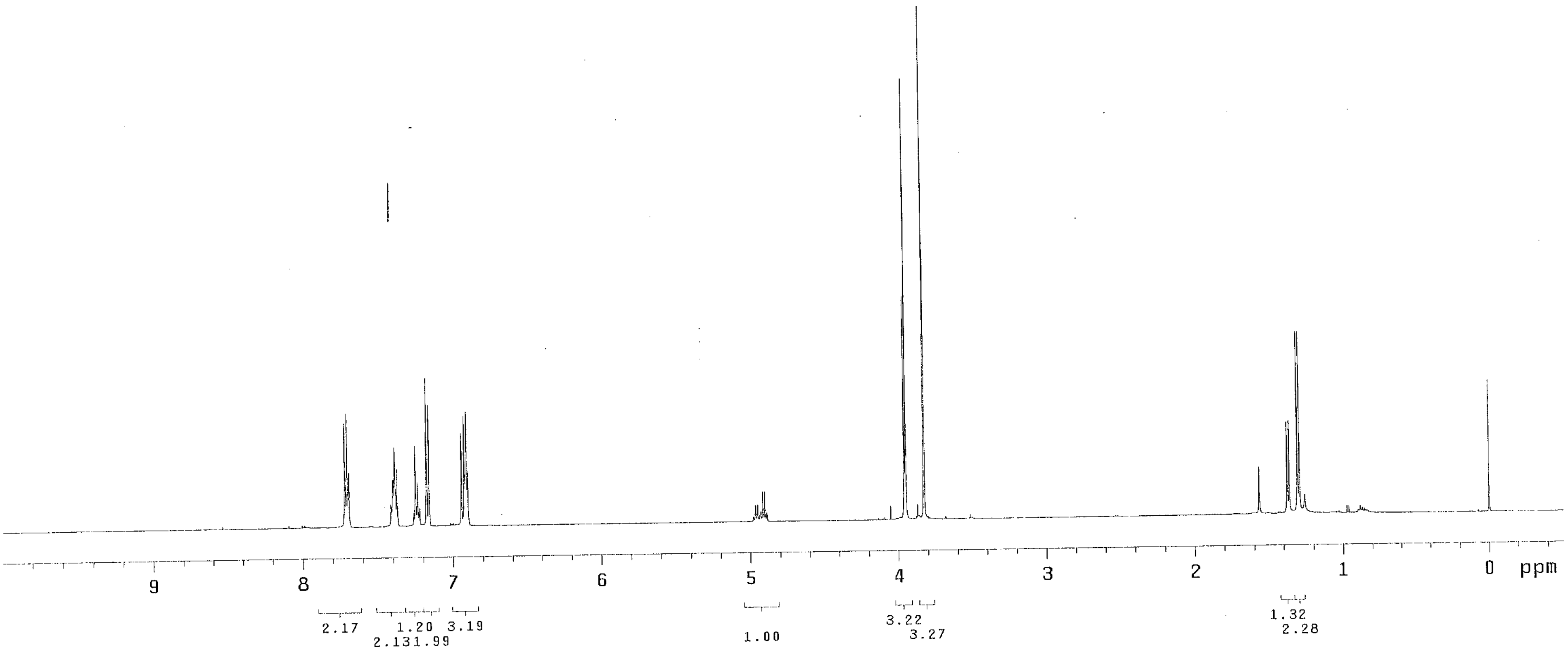

1-Phenoxycarbonyl-2-methyl-3-methoxy-5-(4-methoxyphenyl)-1,2-dihydropyrazine (7b)

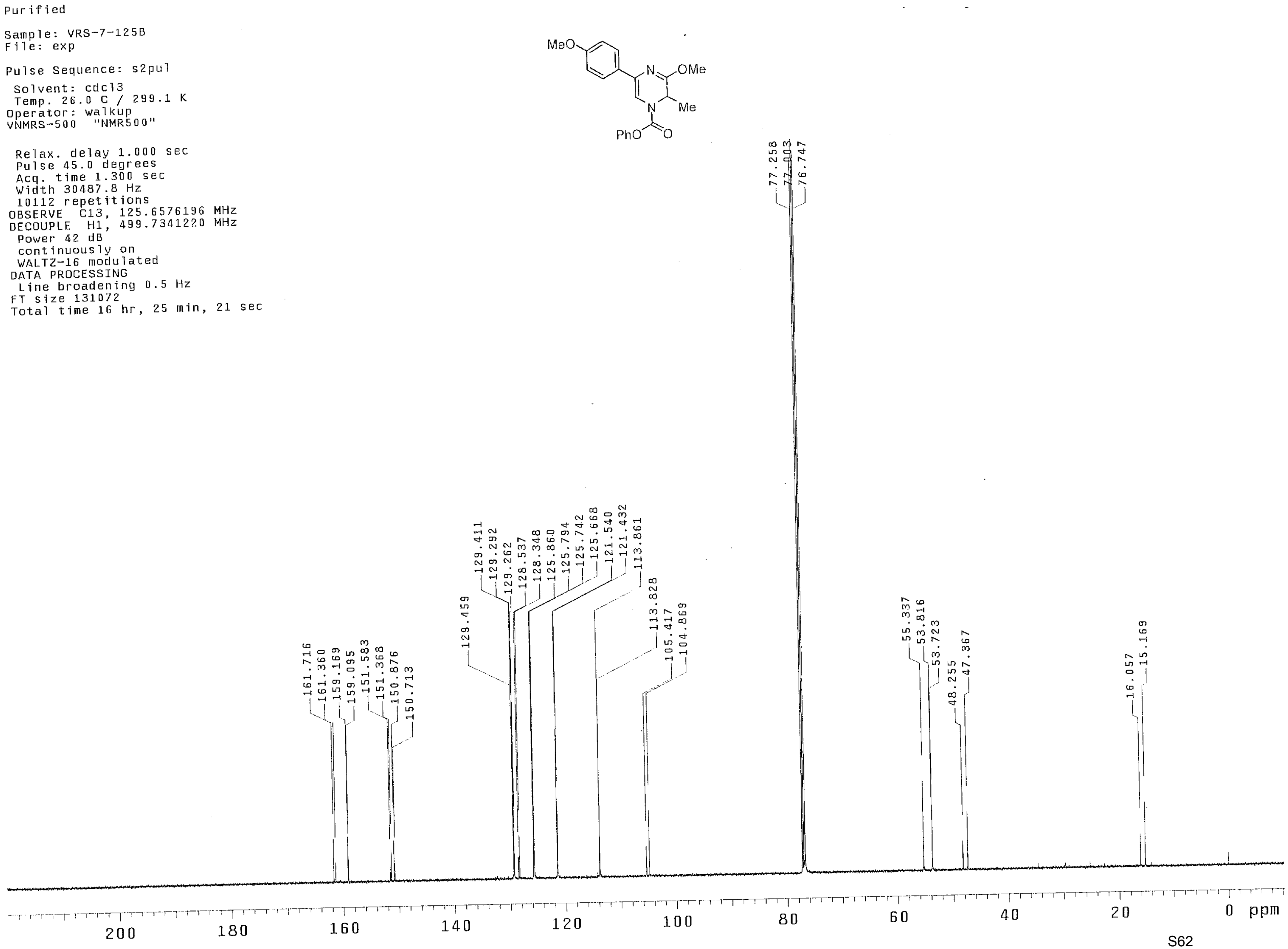

Purified

Sample: VRS-6-108
Sample ID: s_20140416_05
File: s_20140416_05/data/cdcl3_01.fid

Pulse Sequence: s2pul
Solvent: cdcl3
Ambient temperature
Operator: walkup
File: cdcl3_01
VNMRS-500 "NMR500"

Relax. delay 1.000 sec
Pulse 45.0 degrees
Acq. time 2.049 sec
Width 8012.8 Hz
8 repetitions
OBSERVE H1, 499.7316392 MHz
DATA PROCESSING
Resol. enhancement -0.0 Hz
FT size 65536
Total time 0 min, 31 sec

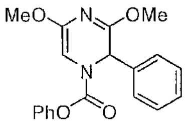

1-Phenoxycarbonyl-2-phenyl-3,5-dimethoxy-1,2-dihydropyrazine (8)

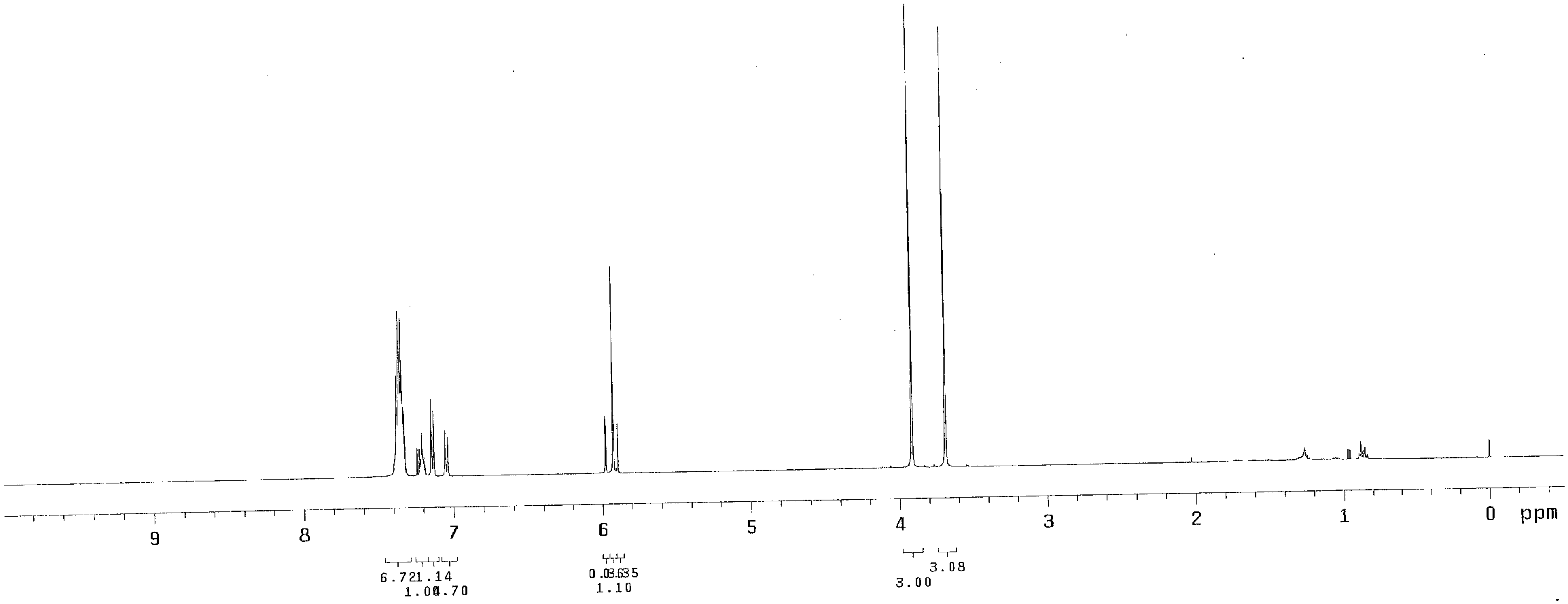

Purified

Sample: VRS-6-108
Sample ID: s_20140416_04
File: s_20140416_04/data/cdcl3_01.fid

Pulse Sequence: s2pul
Solvent: cdcl3
Ambient temperature
Operator: walkup
File: cdcl3_01
VNMRS-500 "NMR500"

Relax. delay 1.000 sec
Pulse 45.0 degrees
Acq. time 1.300 sec
Width 30487.8 Hz
256 repetitions
OBSERVE C13, 125.6576335 MHz
DECOUPLE H1, 499.7341220 MHz
Power 42 dB
continuously on
WALTZ-16 modulated
DATA PROCESSING
Line broadening 0.5 Hz
FT size 131072
Total time 9 min, 51 sec

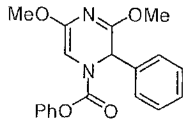

1-Phenoxycarbonyl-2-phenyl-3,5-dimethoxy-1,2-dihydropyrazine (8)

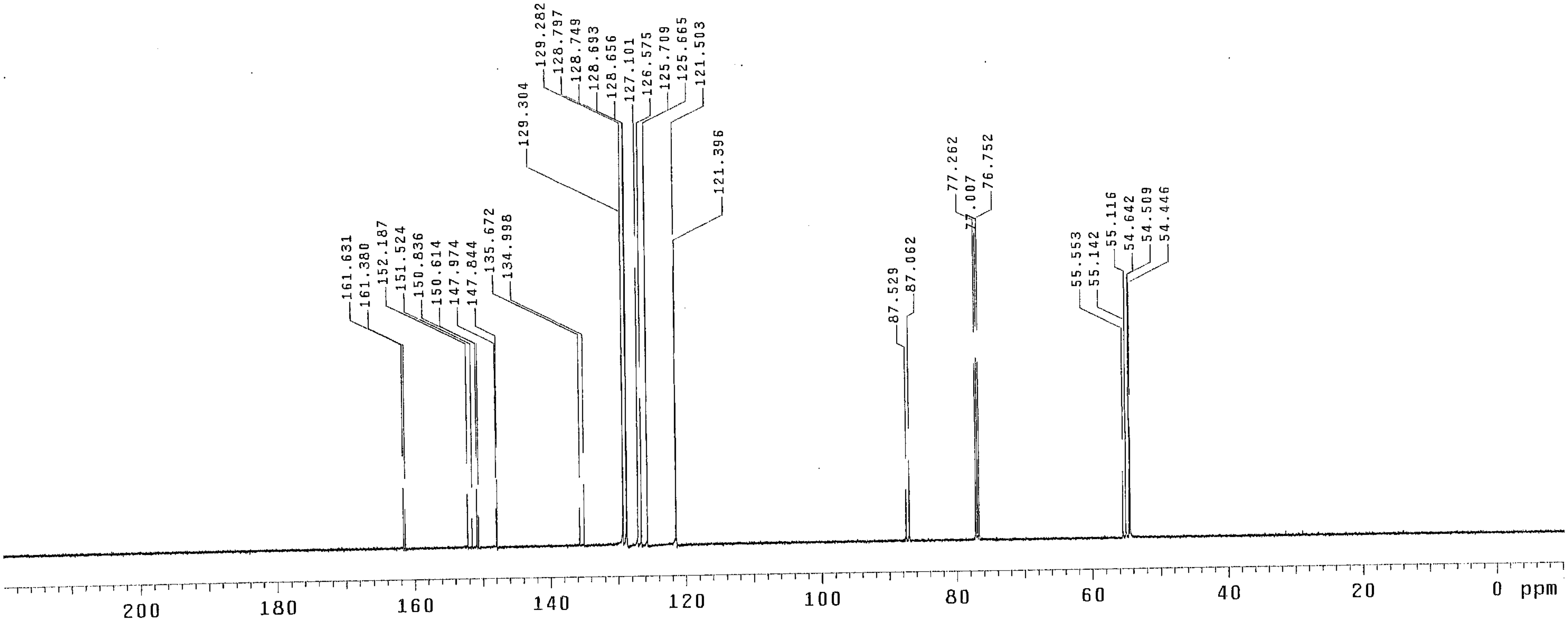

Purified

Sample: VRS-6-133
Sample ID: s_20121214_06
File: /home/walkup/vnmrsys/data/auto_2012.02.02/s_20121214_06/data/cdcl3_01.fid

Pulse Sequence: s2pul
Solvent: cdcl3
Temp. 26.0 C / 299.1 K
Operator: walkup
File: cdcl3_01
VNMRS-500 "NMR500"

Relax. delay 1.000 sec
Pulse 45.0 degrees
Acq. time 2.049 sec
Width 8012.8 Hz
8 repetitions
OBSERVE H1, 499.7316659 MHz
DATA PROCESSING
Resol. enhancement -0.0 Hz
FT size 65536
Total time 0 min, 31 sec

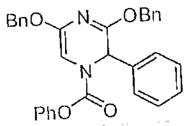

1-Phenoxycarbonyl-2-phenyl-3,5-dibenzyloxy-1,2-dihydropyrazine (9a)

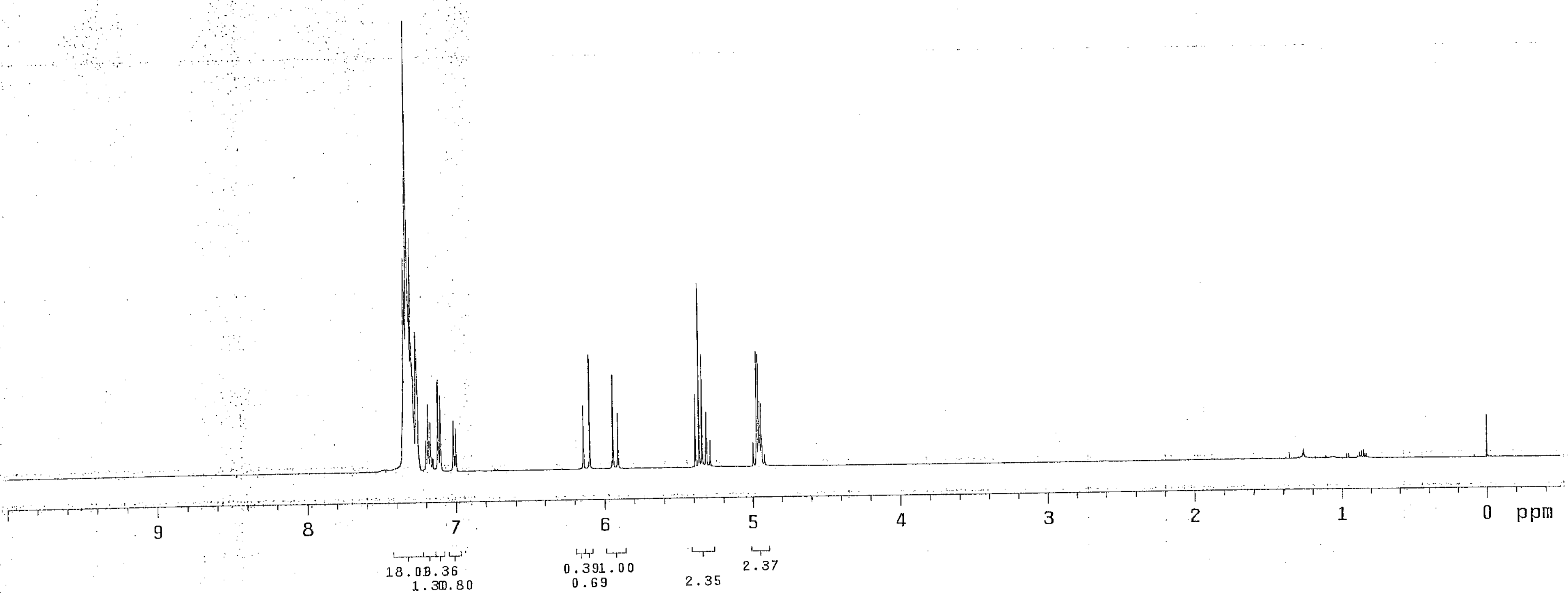

1-Phenoxycarbonyl-2-phenyl-3,5-dibenzyloxy-1,2-dihydropyrazine (9a)

Purified

Sample: VRS-6-133
Sample ID: s_20121214_07
File: /home/walkup/vnmrsys

Pulse Sequence: s2pul
Solvent: cdcl3
Temp. 26.0 C / 299.1 K
Operator: walkup
File: cdcl3_01
VNMRS-500 "NMR500"

Relax. delay 1.000 sec
Pulse 45.0 degrees
Acq. time 1.300 sec
Width 30487.8 Hz
256 repetitions
OBSERVE C13, 125.6576349 MHz
DECOUPLE H1, 499.7341220 MHz
Power 42 dB
continuously on
WALTZ-16 modulated
DATA PROCESSING
Line broadening 0.5 Hz
FT size 131072
Total time 9 min, 51 sec

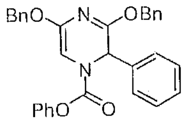

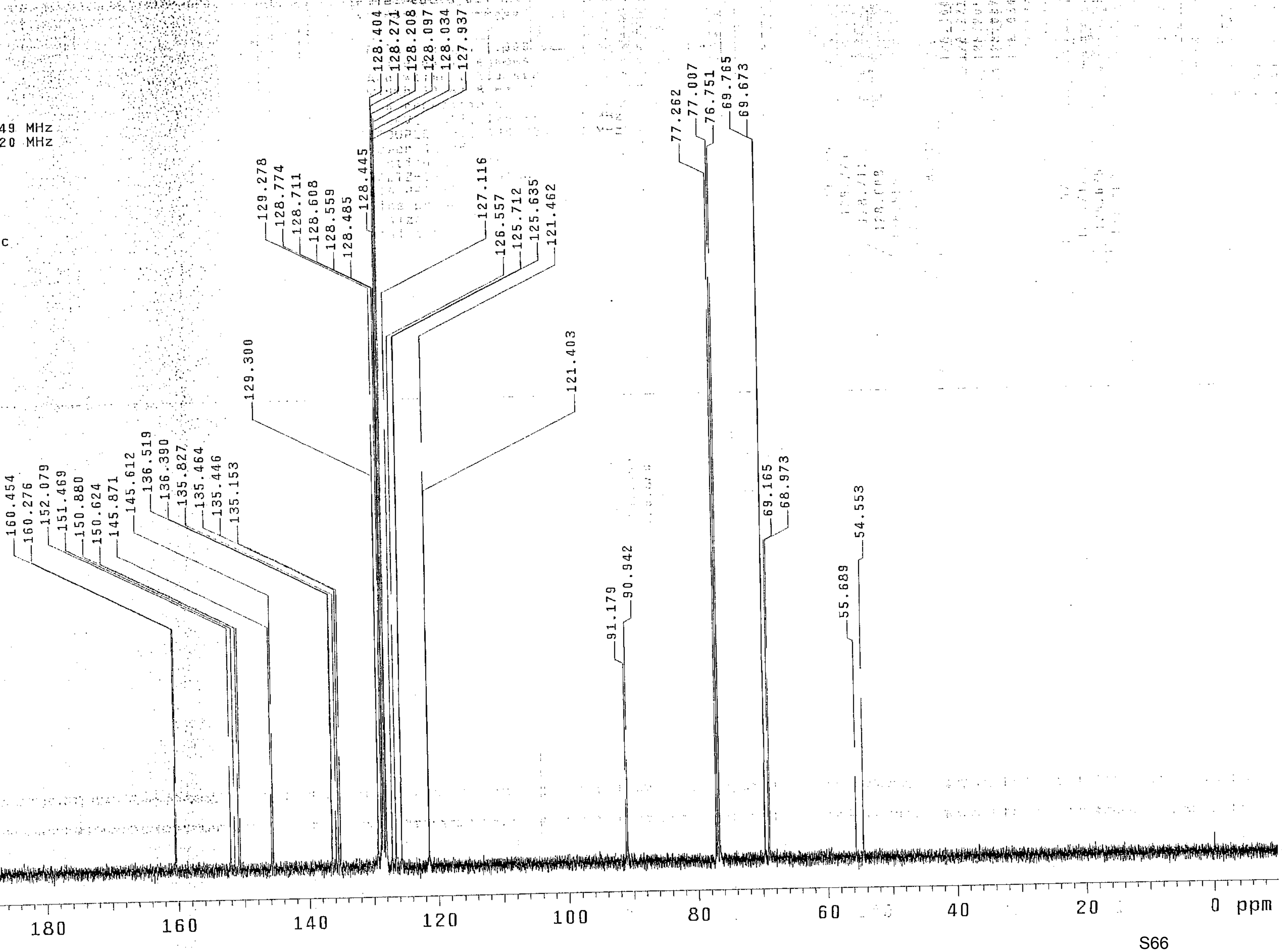

1-Phenoxycarbonyl-2-(4-methoxyphenyl)-3,5-dibenzyloxy-1,2-dihydropyrazine (9b)

target (ISCO)

Sample: VRS-7-109A
Sample ID: s_20140227_10
File: s_20140227_10/data/cdcl3_0

Pulse Sequence: s2pul
Solvent: cdcl3
Ambient temperature
Operator: walkup
File: cdcl3_01
VNMRS-500 "NMR500"

Relax. delay 1.000 sec
Pulse 45.0 degrees
Acq. time 2.049 sec
Width 8012.8 Hz
8 repetitions
OBSERVE H1, 499.7316378 MHz
DATA PROCESSING
Resol. enhancement -0.0 Hz
FT size 65536
Total time 0 min, 31 sec

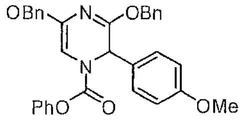

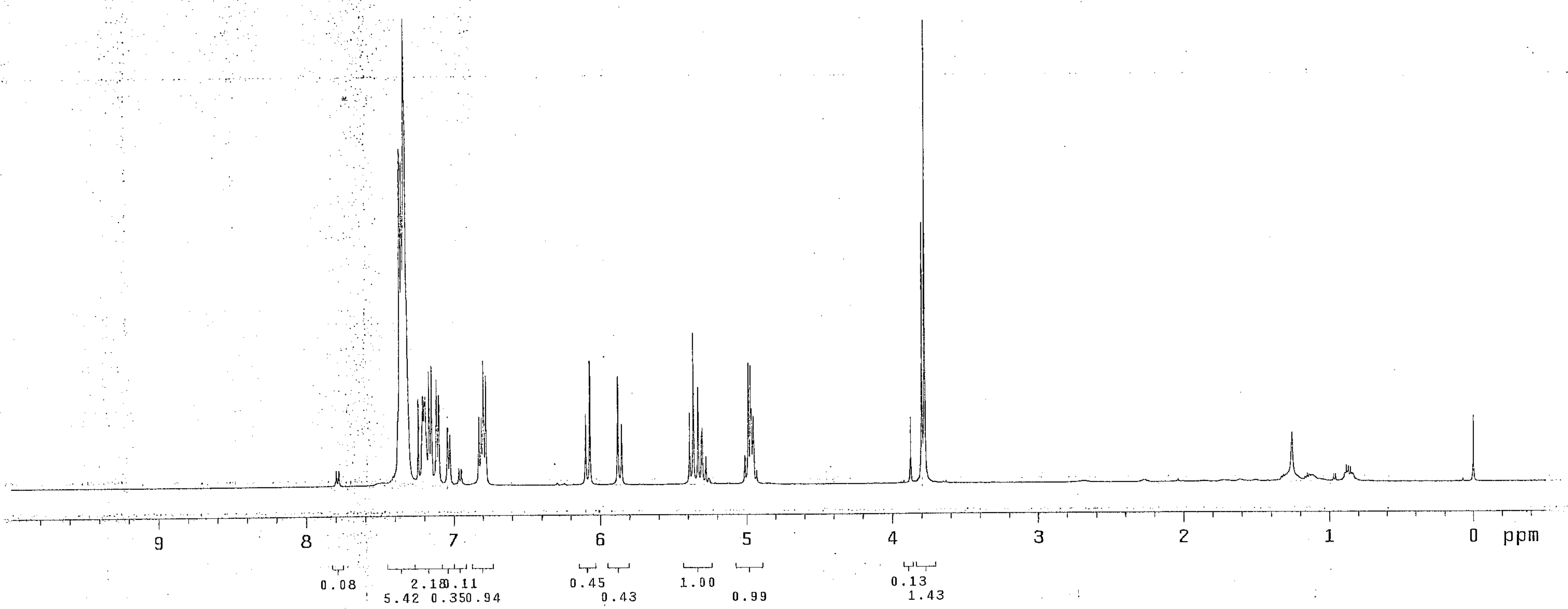

1-Phenoxycarbonyl-2-(4-methoxyphenyl)-3,5-dibenzyloxy-1,2-dihydropyrazine (9b)

Target

Sample: VRS-7-109A
Sample ID: s_20140222_02
File: s_20140222_02/data/cd[illegible]

Pulse Sequence: s2pul
Solvent: cdcl3
Ambient temperature
Operator: walkup
File: cdcl3_01
VNMRS-500 "NMR500"

Relax. delay 1.000 sec
Pulse 45.0 degrees
Acq. time 1.300 sec
Width 30487.8 Hz
256 repetitions
OBSERVE C13, 125.6576493 MHz
DECOUPLE H1, 499.7341220 MHz
Power 42 dB
continuously on
WALTZ-16 modulated
DATA PROCESSING
Line broadening 0.5 Hz
FT size 131072
Total time 9 min, 51 sec

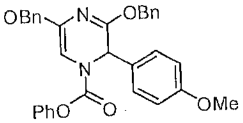

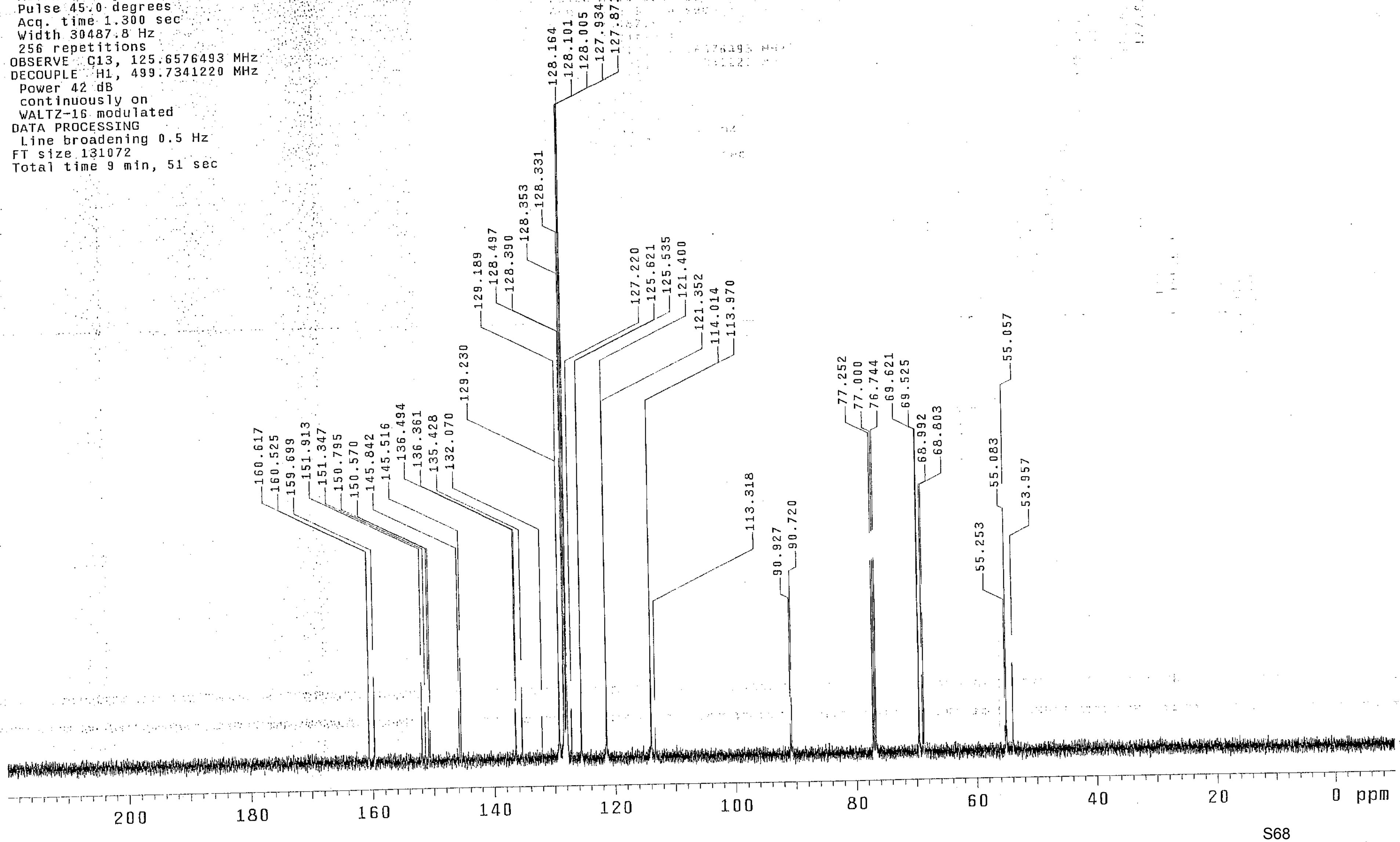

Purified Sec I

Sample: VRS-7-116
Sample ID: s_20131126_03
File: s_20131126_03/data/cdcl3_01.fid

Pulse Sequence: s2pul
Solvent: cdcl3
Temp. 26.0 C / 299.1 K
Operator: walkup
File: cdcl3_01
VNMRS-500 "NMR500"

Relax. delay 1.000 sec
Pulse 45.0 degrees
Acq. time 2.049 sec
Width 8012.8 Hz
8 repetitions
OBSERVE H1, 499.7316405 MHz
DATA PROCESSING
Resol. enhancement -0.0 Hz
FT size 65536
Total time 0 min, 31 sec

1-Phenoxycarbonyl-2-methyl-3-(benzyloxy)-5-ethyl-1,2-dihydropyrazine (10)

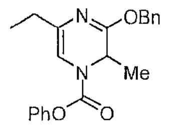

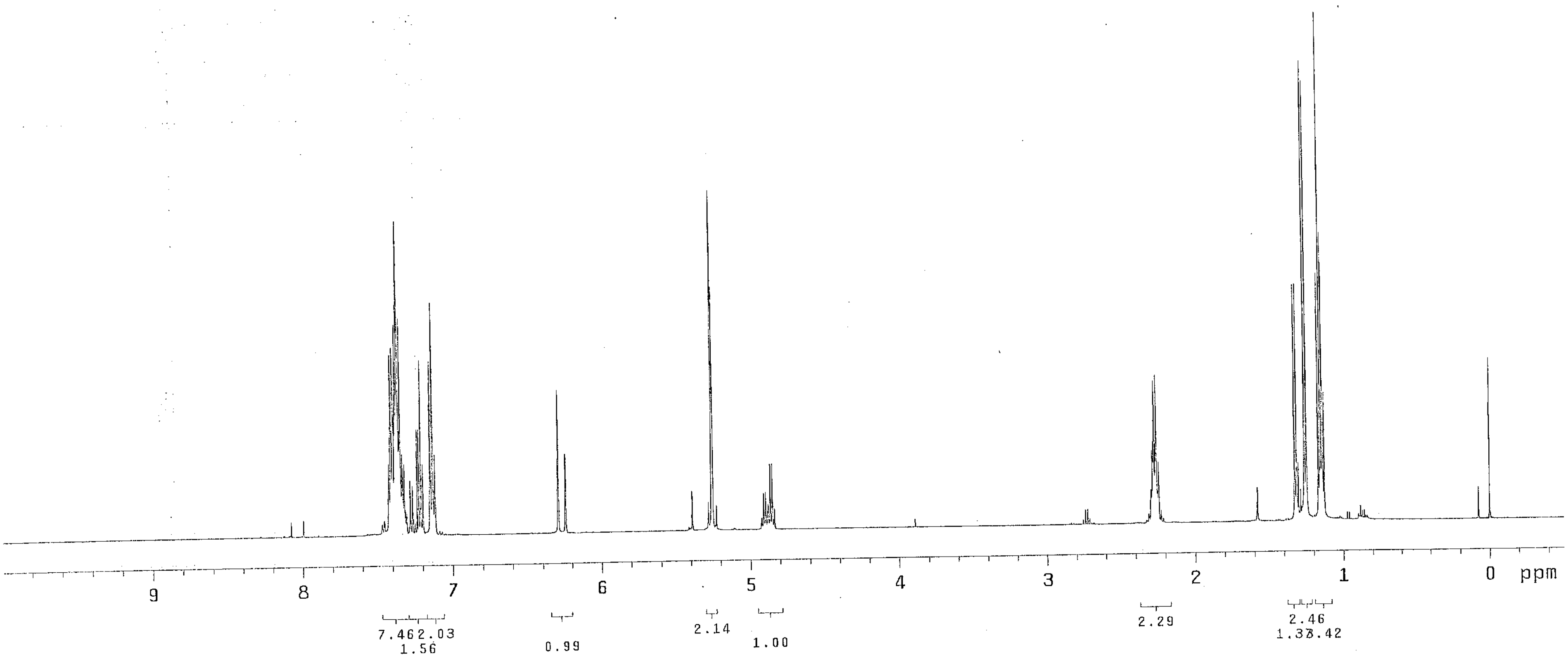

1-Phenoxycarbonyl-2-methyl-3-(benzyloxy)-5-ethyl-1,2-dihydropyrazine (10)

Isolated

Sample: VRS-7-116
Sample ID: s_20140108_03
File: s_20140108_03/data/cdcl3

Pulse Sequence: s2pul
Solvent: cdcl3
Temp. 26.0 C / 299.1 K
Operator: walkup
File: cdcl3_01
VNMRS-500 "NMR500"

Relax. delay 1.000 sec
Pulse 45.0 degrees
Acq. time 1.300 sec
Width 30487.8 Hz
256 repetitions
OBSERVE C13, 125.6576166 MHz
DECOUPLE H1, 499.7341220 MHz
Power 42 dB
continuously on
WALTZ-16 modulated
DATA PROCESSING
Line broadening 0.5 Hz
FT size 131072
Total time 9 min, 51 sec

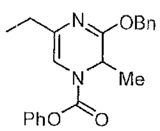

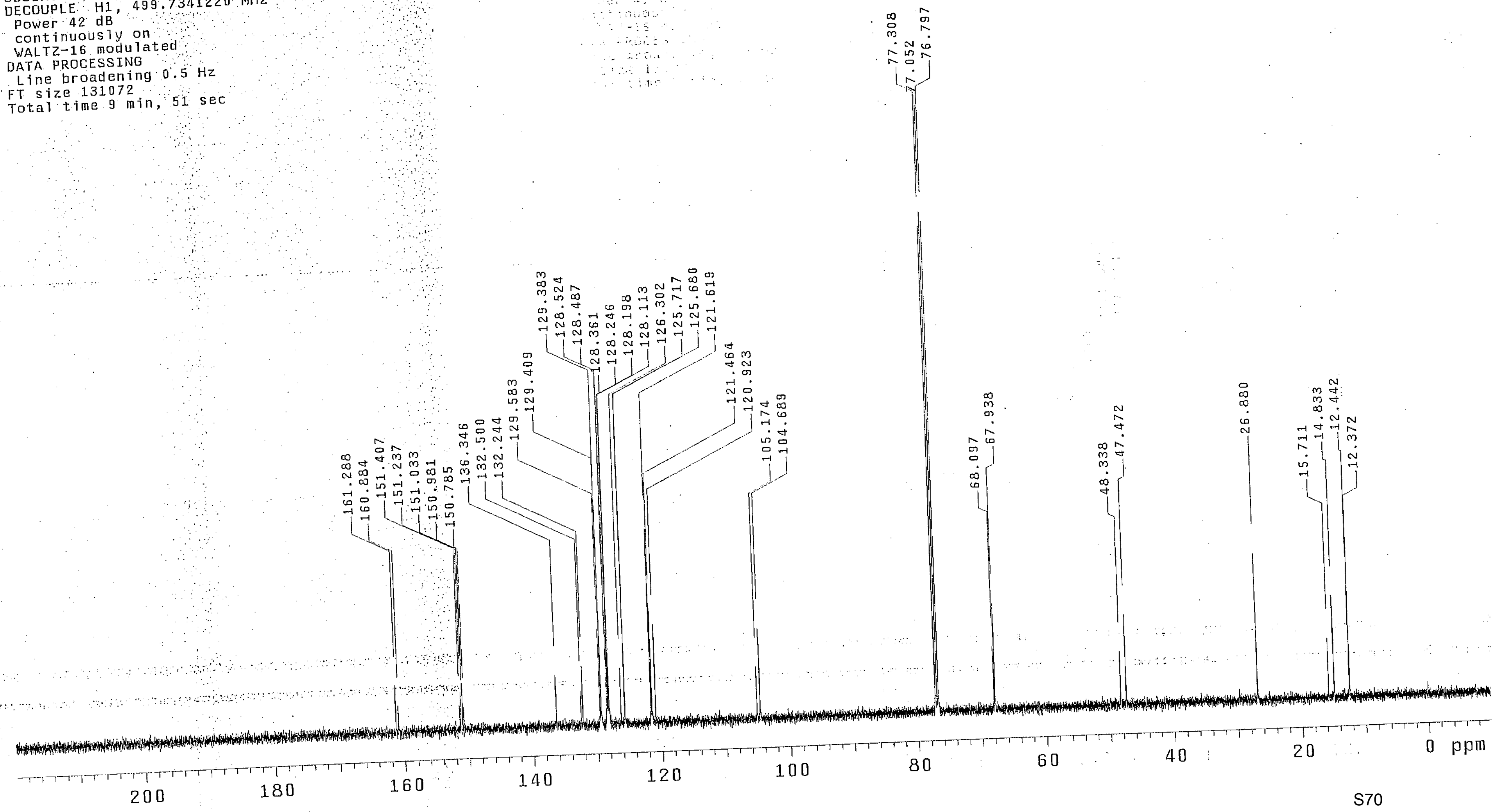

1-Phenoxycarbonyl-2-phenyl-3-benzyloxy-5-benzyl-1,2-dihydropyrazine (11)

Target (ISCO)

Sample: VRS-6-126B
Sample ID: s_20140317_06
File: s_20140317_06/data/cdcl3_01.f

Pulse Sequence: s2pul
Solvent: cdcl3
Temp. 26.0 C / 299.1 K
Operator: walkup
File: cdcl3_01
VNMRS-500 "NMR500"

Relax. delay 1.000 sec
Pulse 45.0 degrees
Acq. time 2.049 sec
Width 8012.8 Hz
8 repetitions
OBSERVE H1, 499.7316617 MHz
DATA PROCESSING
Resol. enhancement -0.0 Hz
FT size 65536
Total time 0 min, 31 sec

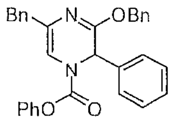

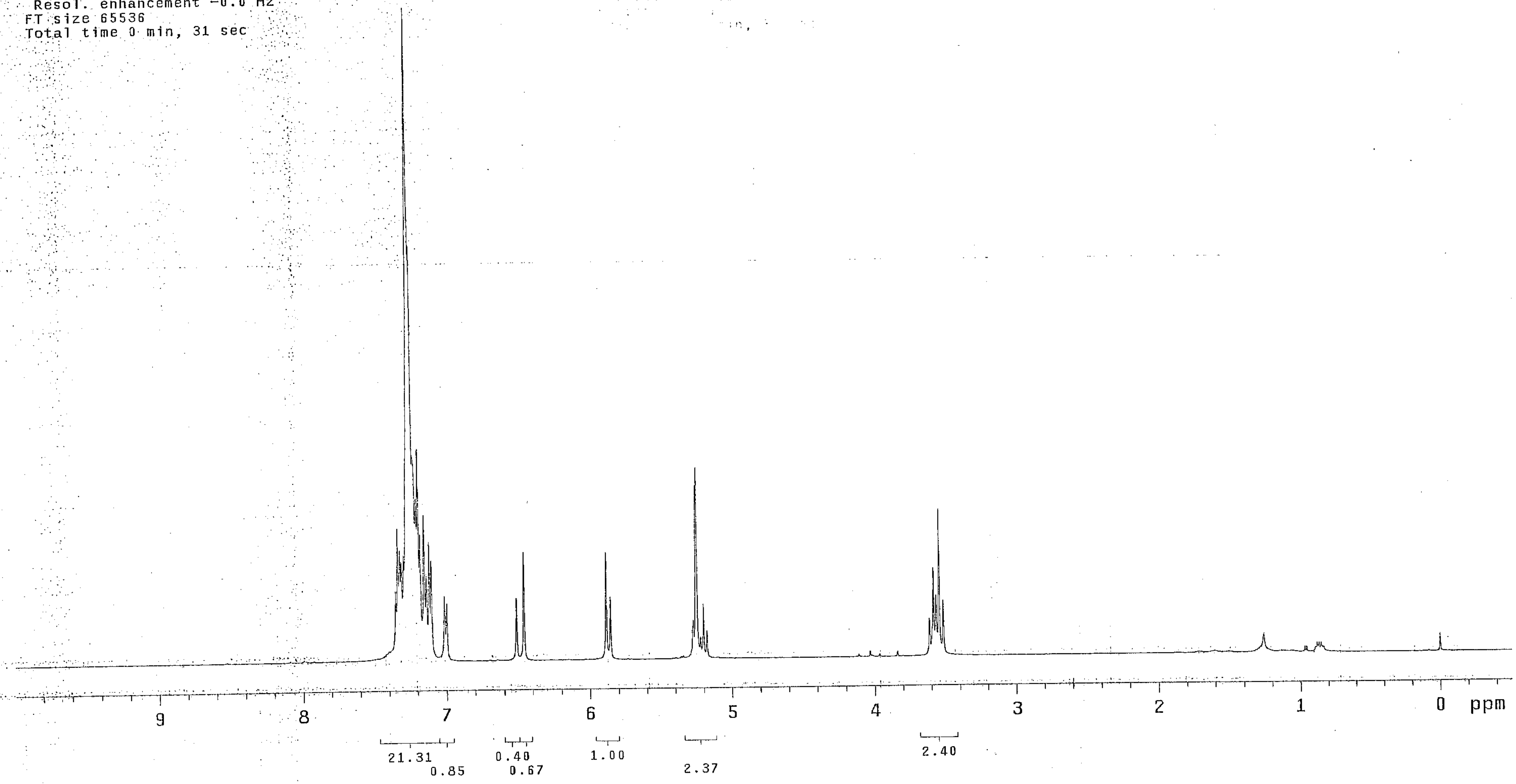

1-Phenoxycarbonyl-2-phenyl-3-benzyloxy-5-benzyl-1,2-dihydropyrazine (11)

Target (ISCO)

Sample: VRS-6-126B
Sample ID: s_20140317_07
File: s_20140317_07/data/cdcl3_01.fi

Pulse Sequence: s2pul
Solvent: cdcl3
Temp. 26.0 C / 299.1 K
Operator: walkup
File: cdcl3_01
VNMRS-500 "NMR500"

Relax. delay 1.000 sec
Pulse 45.0 degrees
Acq. time 1.300 sec
Width 30487.8 Hz
256 repetitions
OBSERVE C13, 125.6576285 MHz
DECOUPLE H1, 499.7341220 MHz
Power 42 dB
continuously on
WALTZ-16 modulated
DATA PROCESSING
Line broadening 0.5 Hz
FT size 131072
Total time 9 min, 51 sec

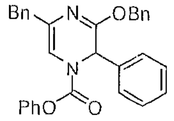

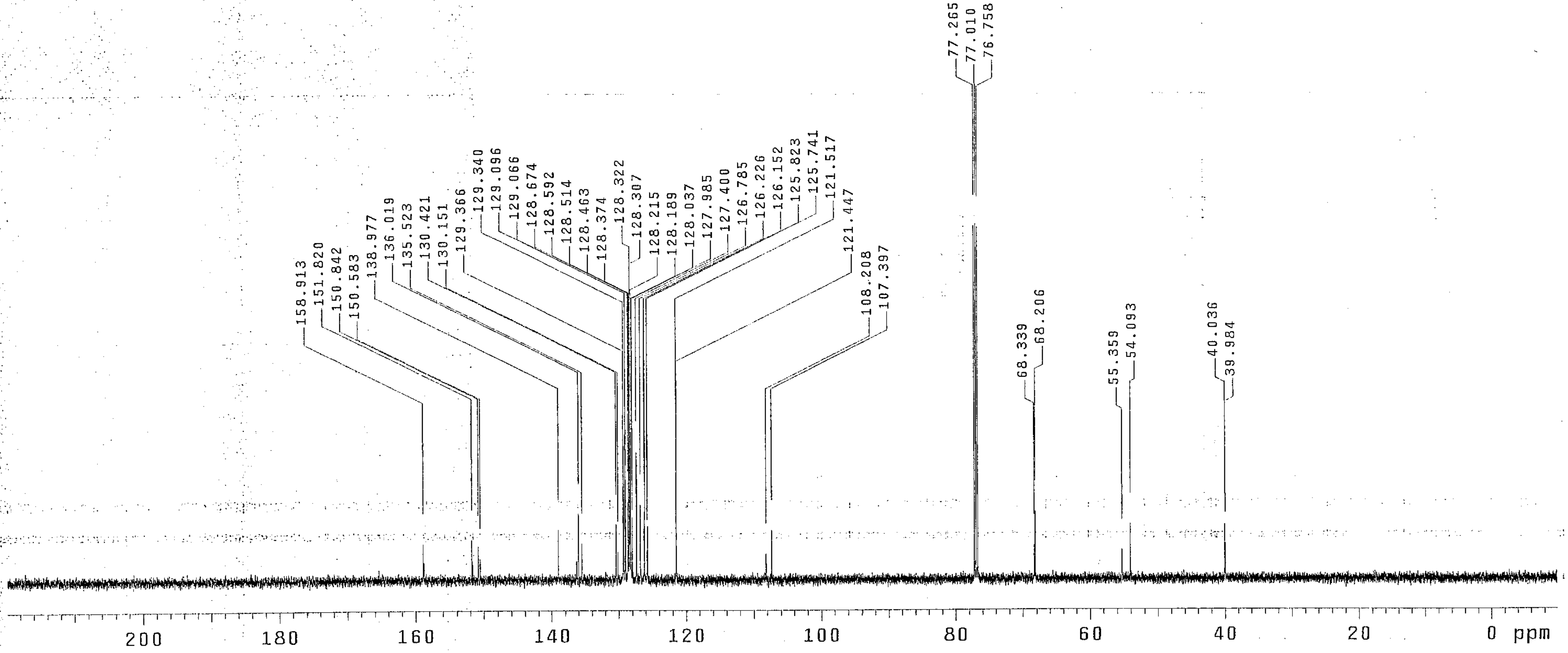

1-Phenoxycarbonyl-2,5-diphenyl-3-benzyloxy-1,2-dihydropyrazine (12)

Purify

Sample: VRS-6-123b
Sample ID: s_20121025_05
File: /home/walkup/vnmrsy

Pulse Sequence: s2pul

Solvent: cdcl3
Temp. 26.0 C / 299.1 K
Operator: walkup
File: cdcl3_01
VNMRS-500 "NMR500"

Relax. delay 1.000 sec
Pulse 45.0 degrees
Acq. time 2.049 sec
Width 8012.8 Hz
8 repetitions
OBSERVE H1, 499.7316366 MHz
DATA PROCESSING
Resol. enhancement -0.0 Hz
FT size 65536
Total time 0 min, 31 sec

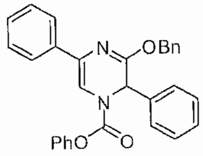

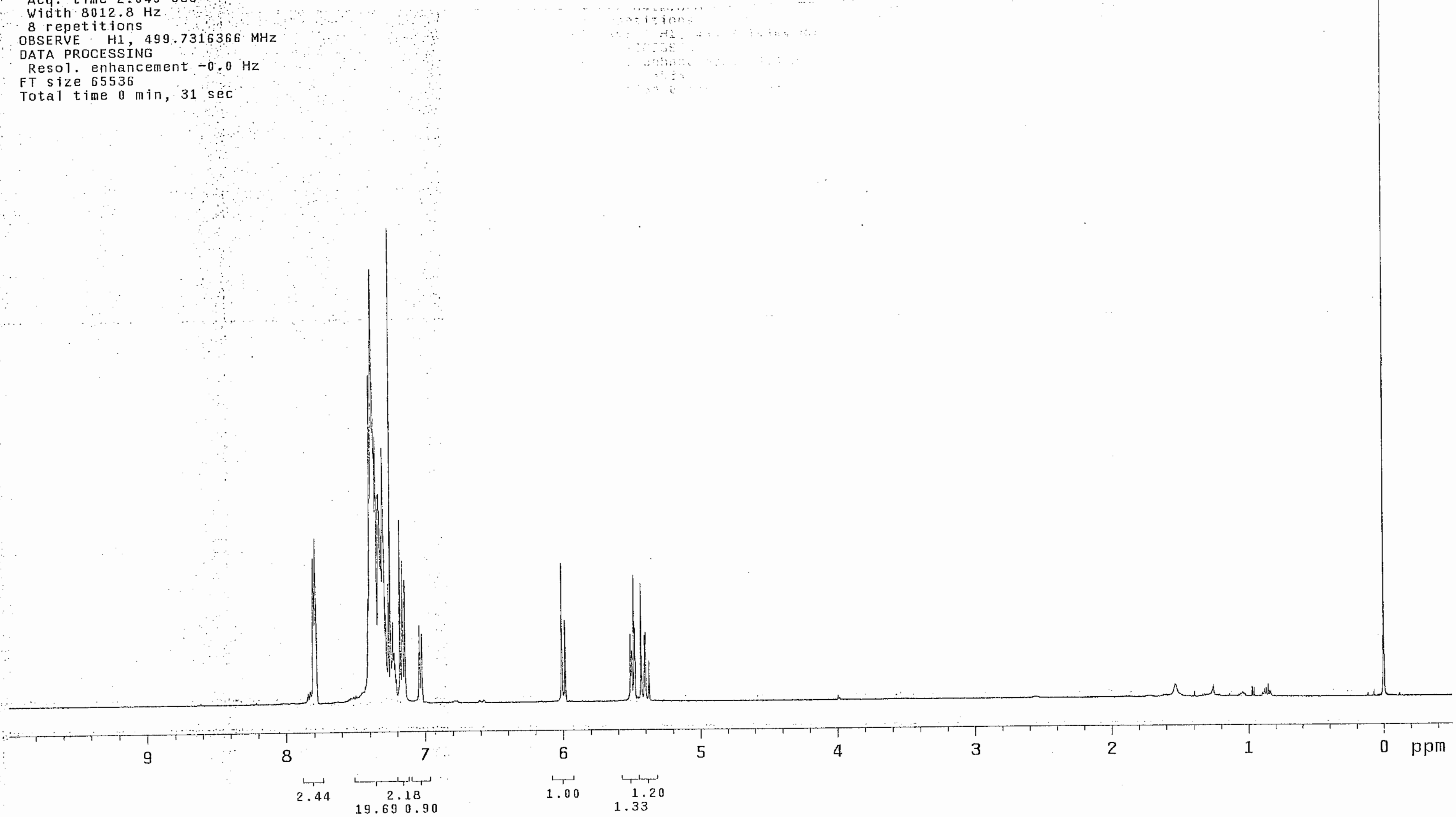

Purified (ISCO)

1-Phenoxycarbonyl-2,5-diphenyl-3-benzyloxy-1,2-dihydropyrazine (12)

Sample: VRS-6-123b
File: exp

Pulse Sequence: s2pul

Solvent: cdcl3
Temp. 26.0 C / 299.1 K
Operator: walkup
VNMRS-500 "NMR500"

Relax. delay 1.000 sec
Pulse 45.0 degrees
Acq. time 1.300 sec
Width 30487.8 Hz
10048 repetitions
OBSERVE C13, 125.6576214 MHz
DECOUPLE H1, 499.7341220 MHz
Power 42 dB
continuously on
WALTZ-16 modulated
DATA PROCESSING
Line broadening 0.5 Hz
FT size 131072
Total time 16 hr, 25 min, 21 sec

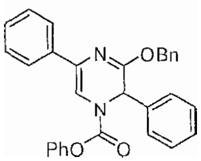

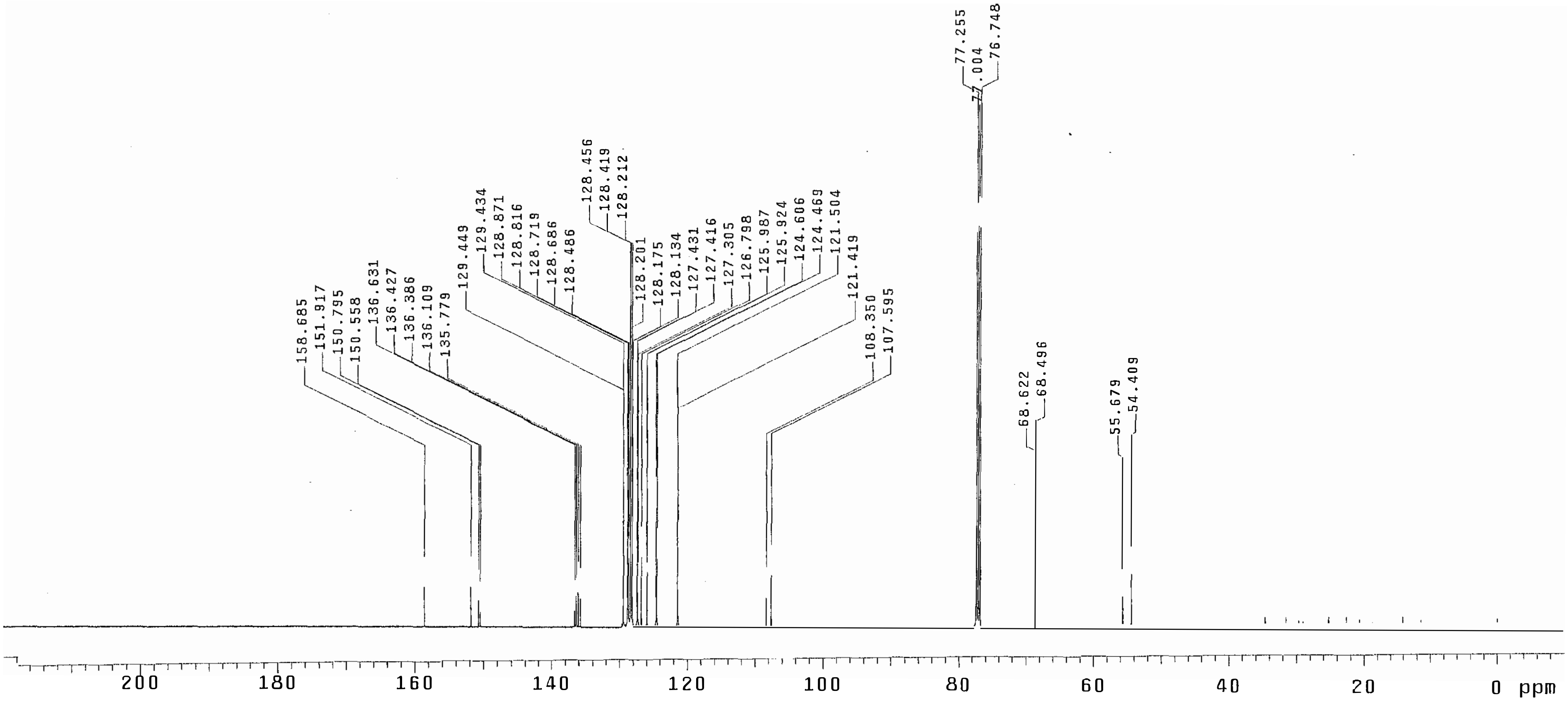

Target (ISCO)

Sample: VRS-7-60
Sample ID: s_20140313_01
File: /home/walkup/vnmrsys/stuc

Pulse Sequence: s2pul

Solvent: cdcl3
Ambient temperature
Operator: walkup
File: cdcl3_01
VNMRS-500 "NMR500"

Relax. delay 1.000 sec
Pulse 45.0 degrees
Acq. time 2.049 sec
Width 8012.8 Hz
8 repetitions
OBSERVE H1, 499.7316331 MHz
DATA PROCESSING
Resol. enhancement -0.0 Hz
FT size 65536
Total time 0 min, 30 sec

1-Phenoxycarbonyl-2-phenyl-3-benzyloxy-5-(benzyloxycarbonyl)-1,2-dihydropyrazine (13)

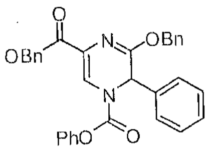

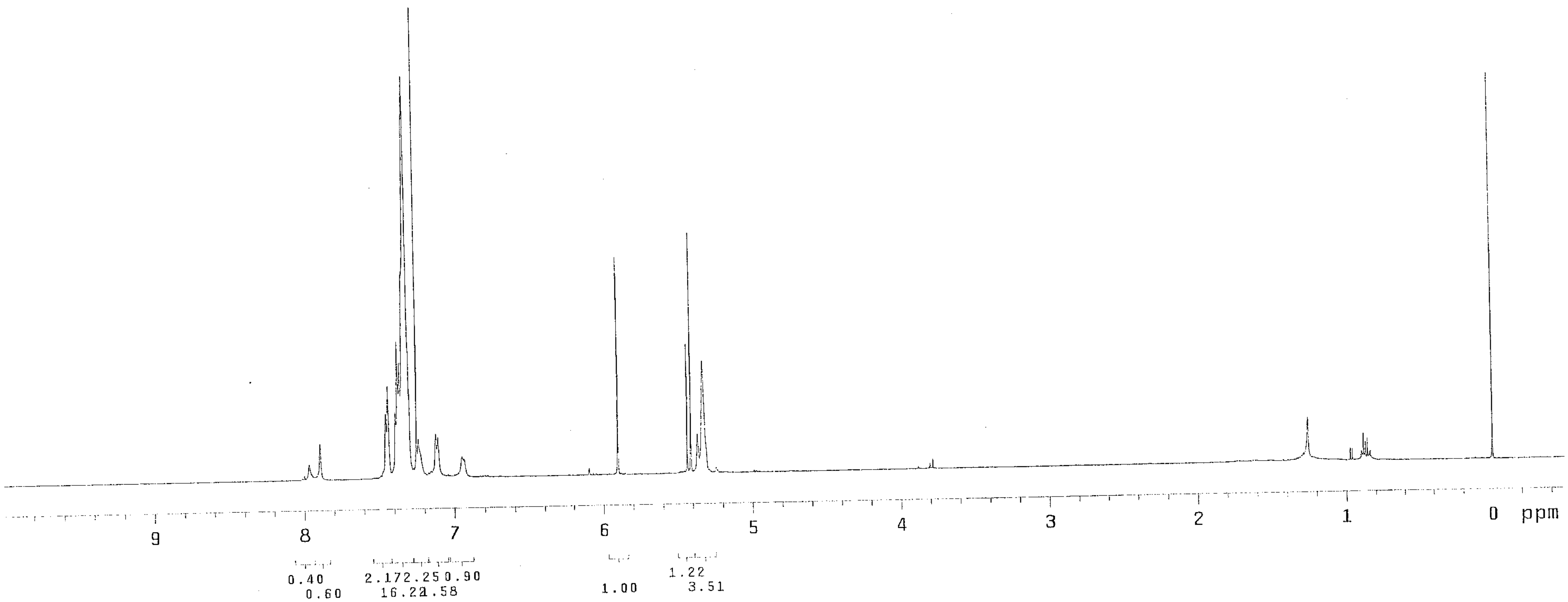

1-Phenoxycarbonyl-2-phenyl-3-benzyloxy-5-(benzyloxycarbonyl)-1,2-dihydropyrazine (13)

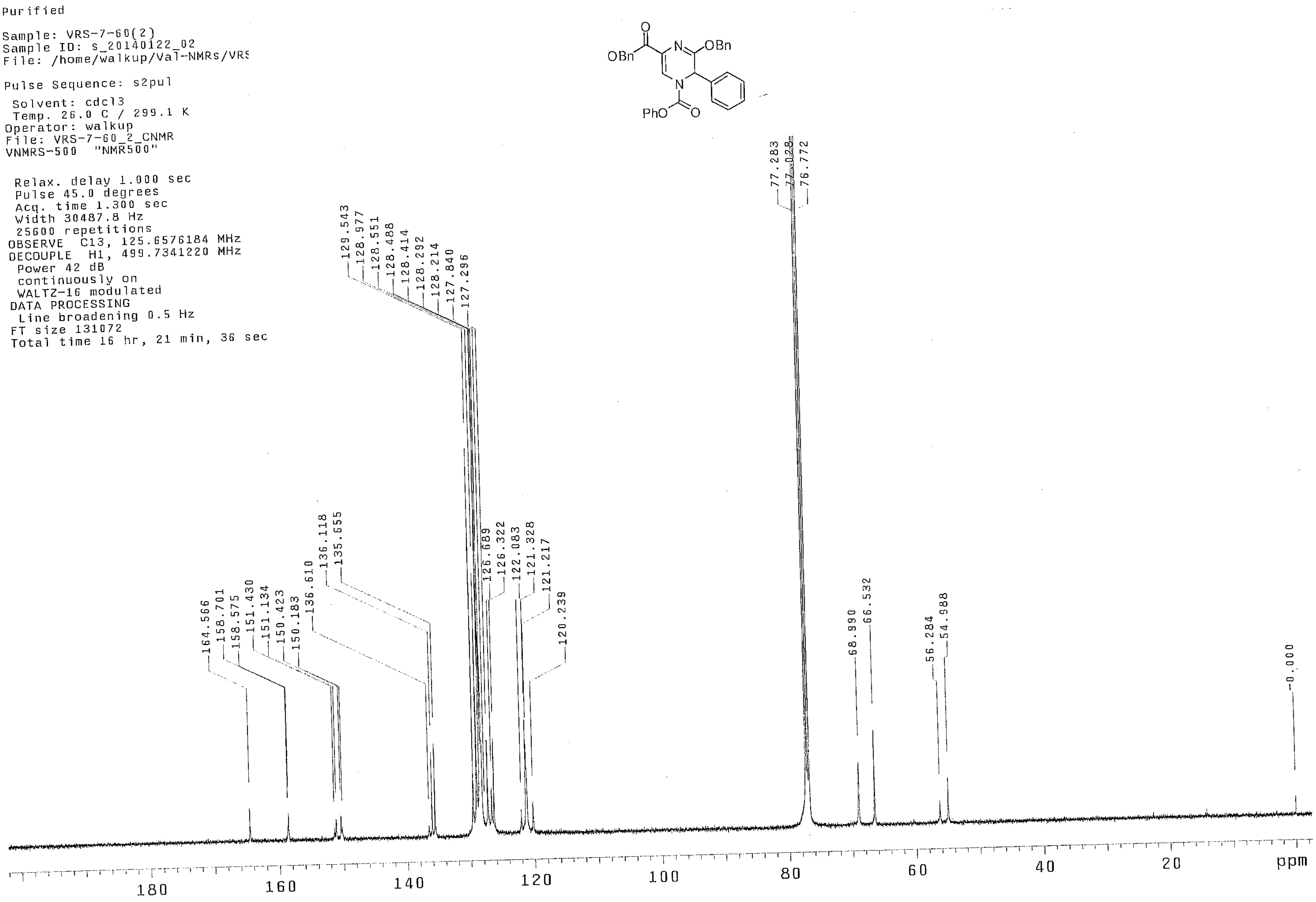

Purified VRS-6-109

Sample: VRS-6-1A
Sample ID: s_20120226_03
File: /home/walkup/vnmrsys/data/[illegible]

Pulse Sequence: s2pul
Solvent: cdcl3
Temp. 26.0 C / 299.1 K
Operator: walkup
File: cdcl3_01
VNMRS-500 "NMR500"

Relax. delay 1.000 sec
Pulse 45.0 degrees
Acq. time 2.049 sec
Width 8012.8 Hz
8 repetitions
OBSERVE H1, 499.7316326 MHz
DATA PROCESSING
Resol. enhancement -0.0 Hz
FT size 65536
Total time 0 min, 31 sec

Phenyl 3-oxo-2-phenyl-3,4-dihydropyrazine-1(2H)-carboxylate (14a)

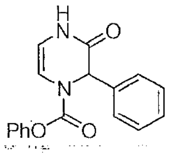

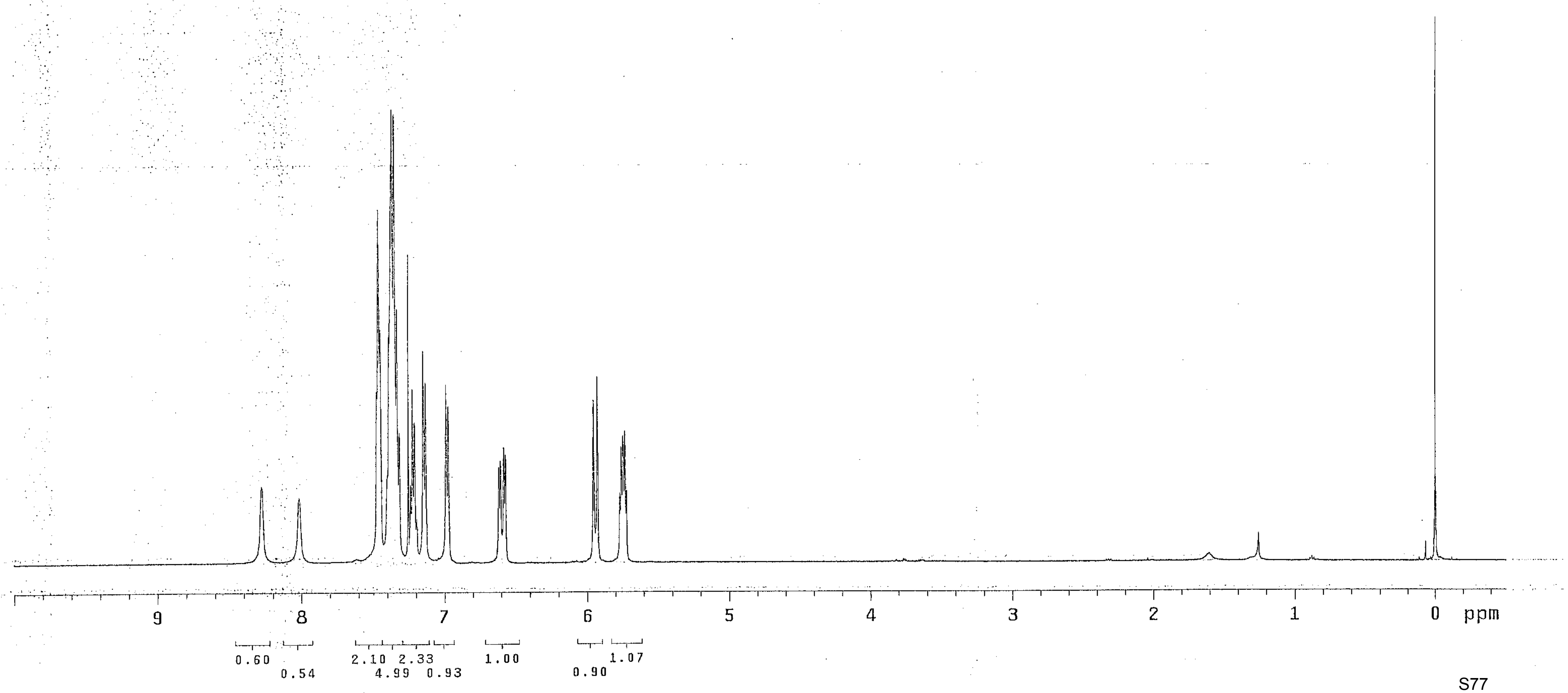

Phenyl 3-oxo-2-phenyl-3,4-dihydropyrazine-1(2H)-carboxylate (14a)

Purified

Sample: VRS-6-109
Sample ID: s_20140214_03
File: s_20140214_03/data/cdc

Pulse Sequence: s2pul
Solvent: cdcl3
Temp. 26.0 C / 299.1 K
Operator: walkup
File: cdcl3_01
VNMRS-500 "NMR500"

Relax. delay 1.000 sec
Pulse 45.0 degrees
Acq. time 1.300 sec
Width 30487.8 Hz
256 repetitions
OBSERVE C13, 125.6576310 MHz
DECOUPLE H1, 499.7341220 MHz
Power 42 dB
continuously on
WALTZ-16 modulated
DATA PROCESSING
Line broadening 0.5 Hz
FT size 131072
Total time 9 min, 51 sec

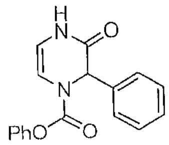

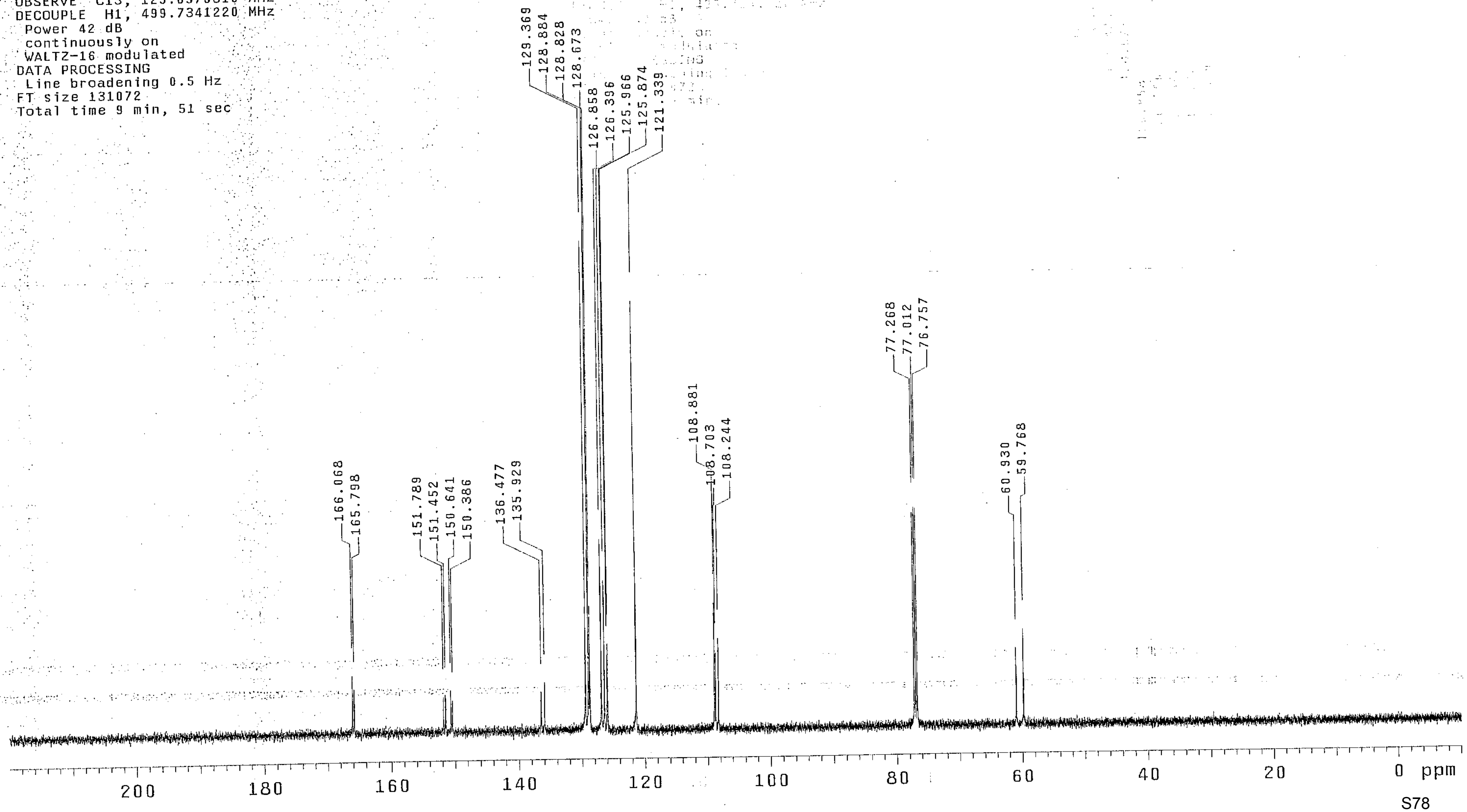

Phenyl 2-benzyl-3-oxo-3,4-dihydropyrazine-1(2H)-carboxylate (14b)

Purified (ISCO)

Sample: VRS-6-51
Sample ID: s_20140421_01
File: s_20140421_01/data/cdcl3_01.fid

Pulse Sequence: s2pul

Solvent: cdcl3
Temp. 26.0 C / 299.1 K
Operator: walkup
File: cdcl3_01
VNMRS-500 "NMR500"

Relax. delay 1.000 sec
Pulse 45.0 degrees
Acq. time 2.049 sec
Width 8012.8 Hz
8 repetitions
OBSERVE H1, 499.7316300 MHz
DATA PROCESSING
Resol. enhancement -0.0 Hz
FT size 65536
Total time 0 min, 31 sec

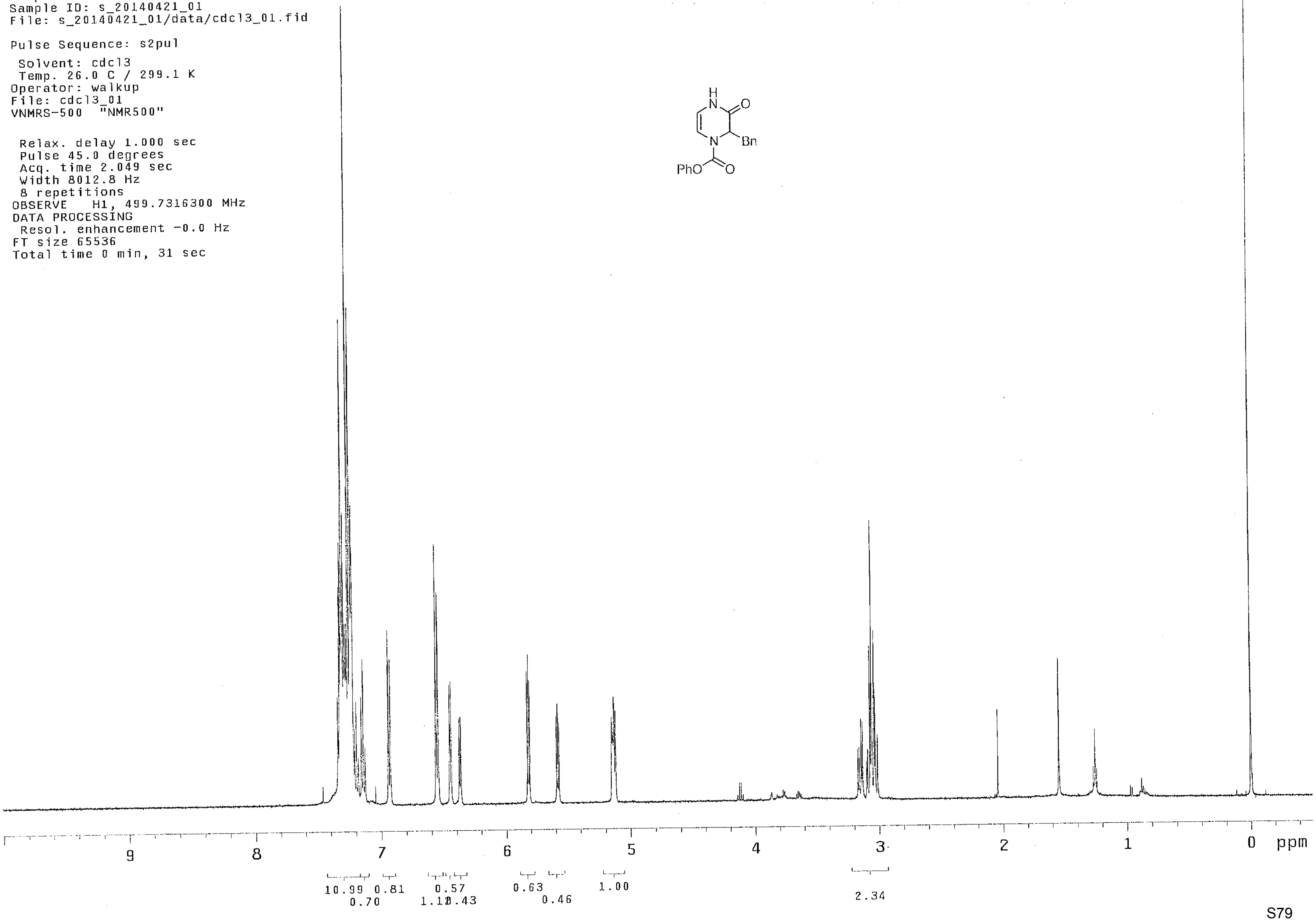

Phenyl 2-benzyl-3-oxo-3,4-dihydropyrazine-1(2H)-carboxylate (14b)

Purified (ISCO)

Sample: VRS-6-51
Sample ID: s_20140422_02
File: s_20140422_02/data/cdcl3_01.fid

Pulse Sequence: s2pul

Solvent: cdcl3
Temp. 26.0 C / 299.1 K
Operator: walkup
File: cdcl3_01
VNMRS-500 "NMR500"

Relax. delay 1.000 sec
Pulse 45.0 degrees
Acq. time 1.300 sec
Width 30487.8 Hz
256 repetitions
OBSERVE C13, 125.6576245 MHz
DECOUPLE H1, 499.7341220 MHz
Power 42 dB
continuously on
WALTZ-16 modulated
DATA PROCESSING
Line broadening 0.5 Hz
FT size 131072
Total time 9 min, 51 sec

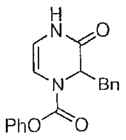

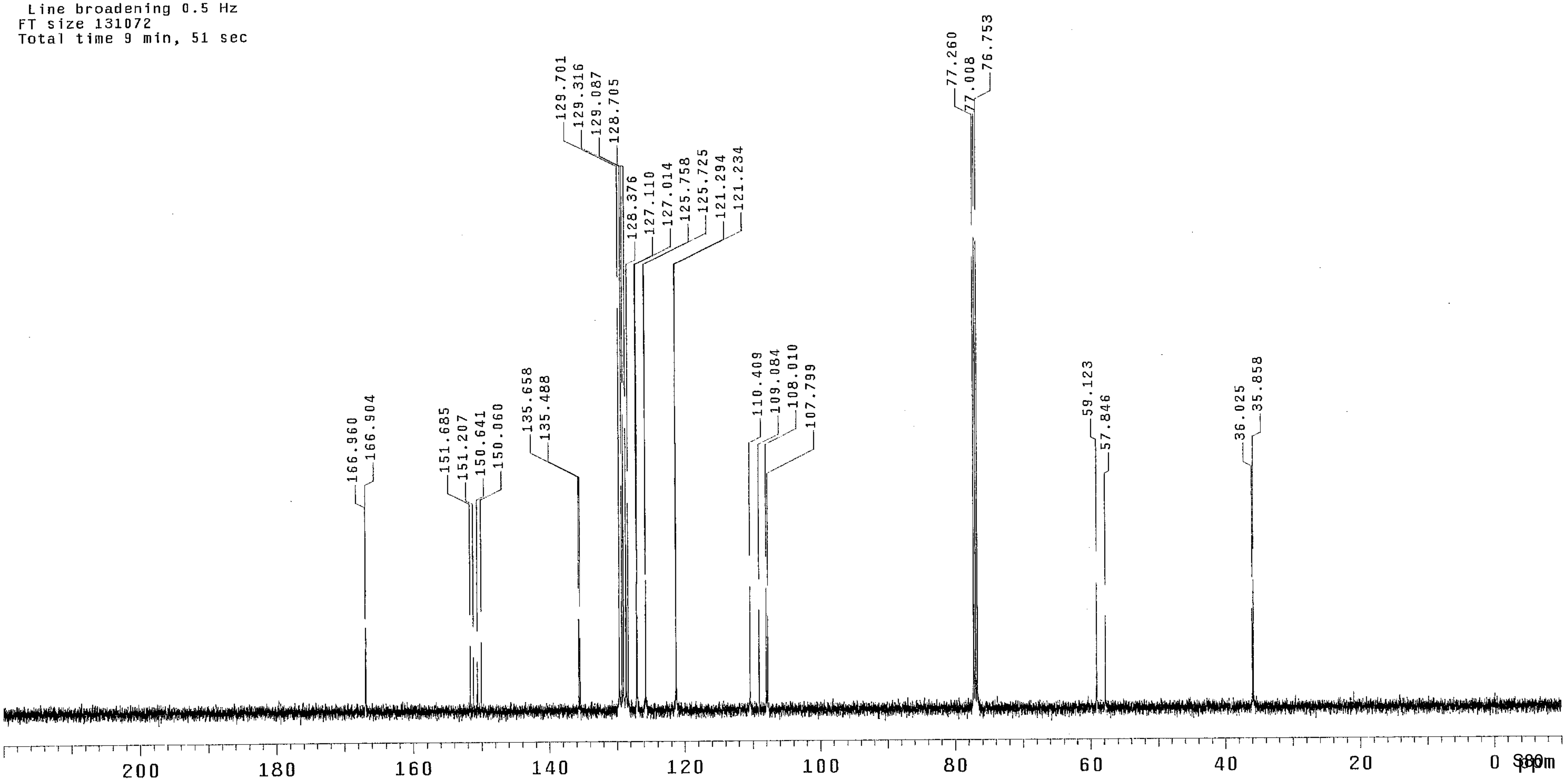

Terget (ISCO)

Sample: YM-1-22
Sample ID: s_20140312_01
File: s_20140312_01/data/cdcl3_01.fid

Pulse Sequence: s2pul
Solvent: cdcl3
Ambient temperature
Operator: walkup
File: cdcl3_01
VNMRS-500 "NMR500"

Relax. delay 1.000 sec
Pulse 45.0 degrees
Acq. time 2.049 sec
Width 8012.8 Hz
8 repetitions
OBSERVE H1, 499.7316302 MHz
DATA PROCESSING
Resol. enhancement -0.0 Hz
FT size 65536
Total time 0 min, 31 sec

Phenyl 2-(2-bromobenzyl)-3-oxo-3,4-dihydropyrazine-1(2H)-carboxylate (14c)

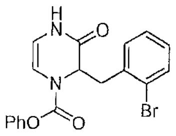

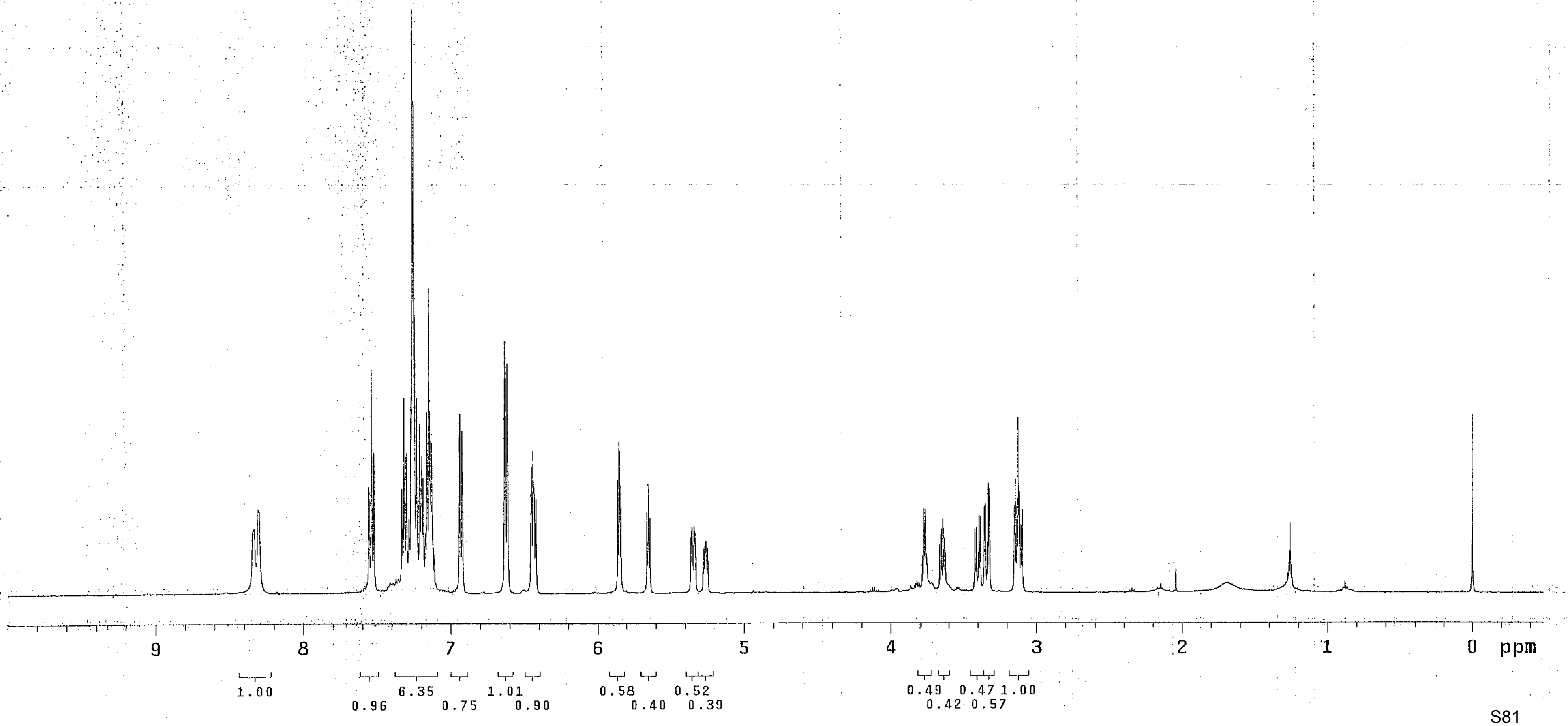

Phenyl 2-(2-bromobenzyl)-3-oxo-3,4-dihydropyrazine-1(2H)-carboxylate (14c)

Hydrolysis (band II)

Sample: YM-1-22
Sample ID: s_20121205_
File: /home/walkup/Val

Pulse Sequence: s2pul
Solvent: cdcl3
Temp. 26.0 C / 299.1
Operator: walkup
File: YM-1-22-CNMR
VNMRS-500 "NMR500"

Relax. delay 1.000 sec
Pulse 45.0 degrees
Acq. time 1.300 sec
Width 30487.8 Hz
25280 repetitions
OBSERVE C13, 125.6576264 MHz
DECOUPLE H1, 499.7341220 MHz
Power 42 dB
continuously on
WALTZ-16 modulated
DATA PROCESSING
Line broadening 0.5 Hz
FT size 131072
Total time 16 hr, 25 min, 21 sec

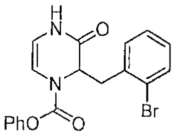

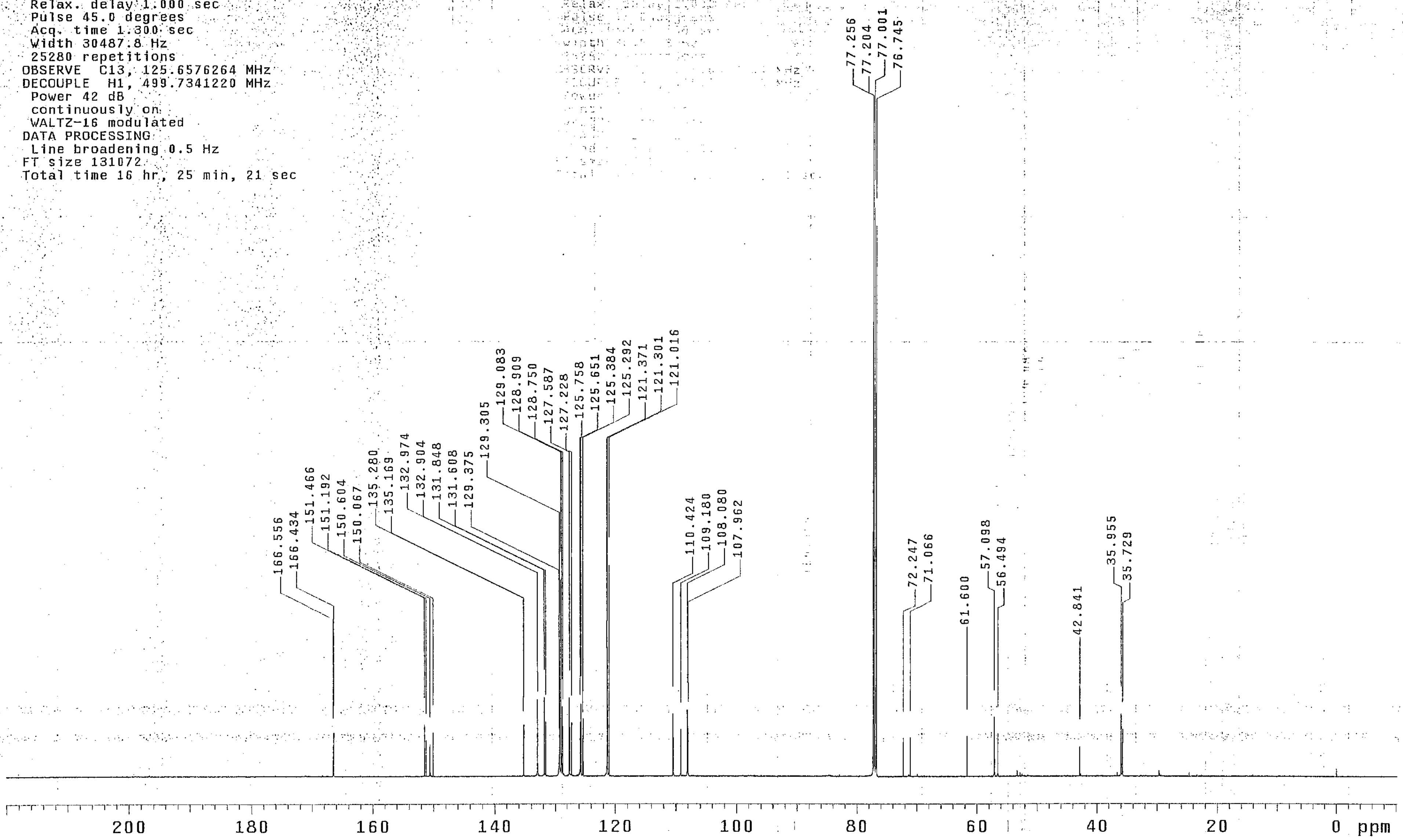

Phenyl 5-(4-methoxyphenyl)-3-oxo-2-phenyl-3,4-dihydropyrazine-1(2H)-carboxylate (15a)

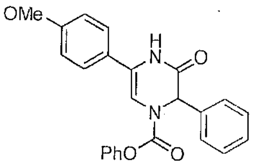

Purified

Sample: VRS-7-108
Sample ID: s_20131112_01
File: /home/walkup/vnmrsys/

Pulse Sequence: s2pul
Solvent: cdcl3
Temp. 26.0 C / 299.1 K
Operator: walkup
File: cdcl3_01
VNMRS-500 "NMR500"

Relax. delay 1.000 sec
Pulse 45.0 degrees
Acq. time 2.049 sec
Width 8012.8 Hz
8 repetitions
OBSERVE H1, 499.7316339 MHz
DATA PROCESSING
Resol. enhancement -0.0 Hz
FT size 65536
Total time 0 min, 31 sec

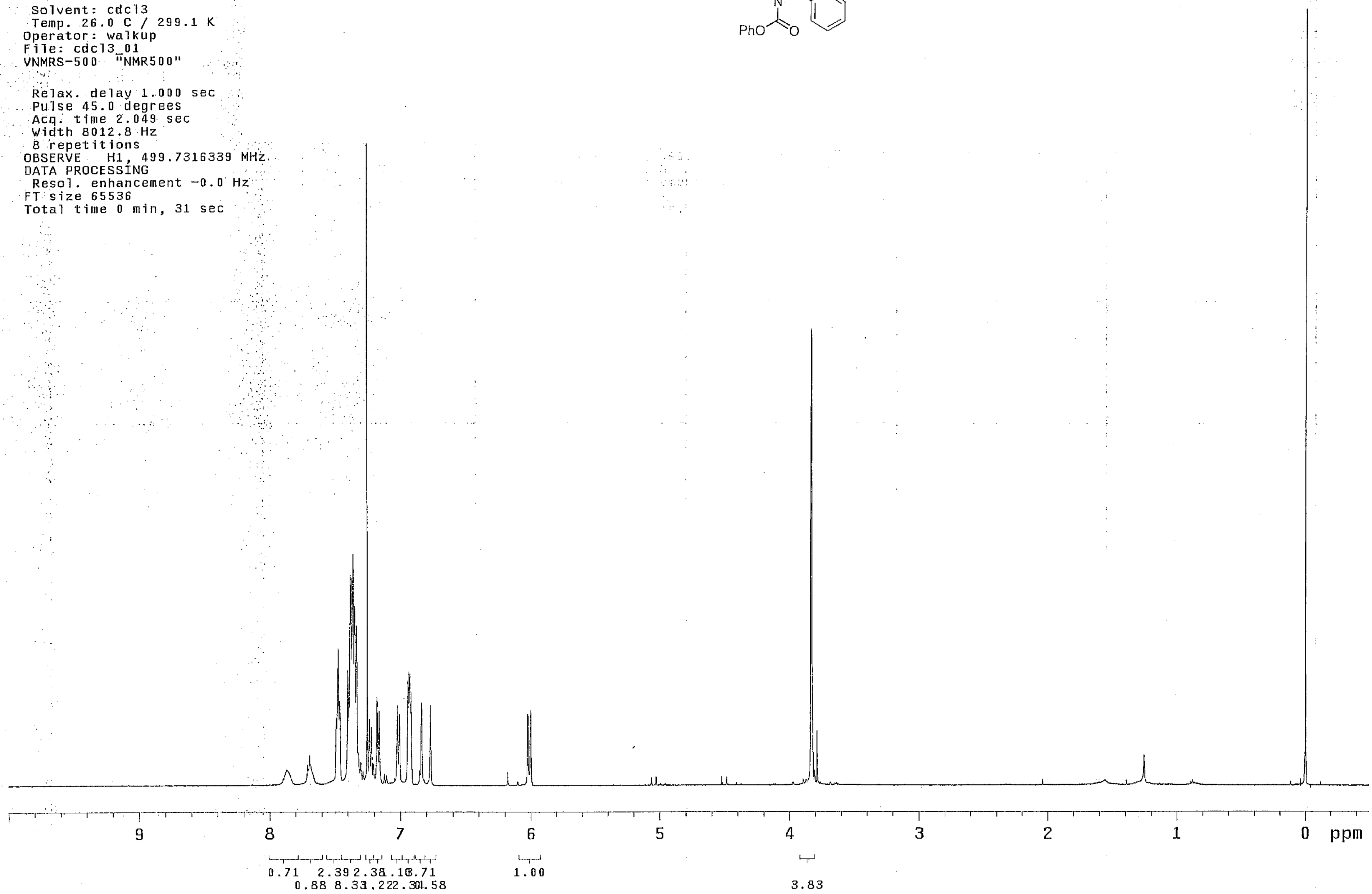

Phenyl 5-(4-methoxyphenyl)-3-oxo-2-phenyl-3,4-dihydropyrazine-1(2H)-carboxylate (15a)

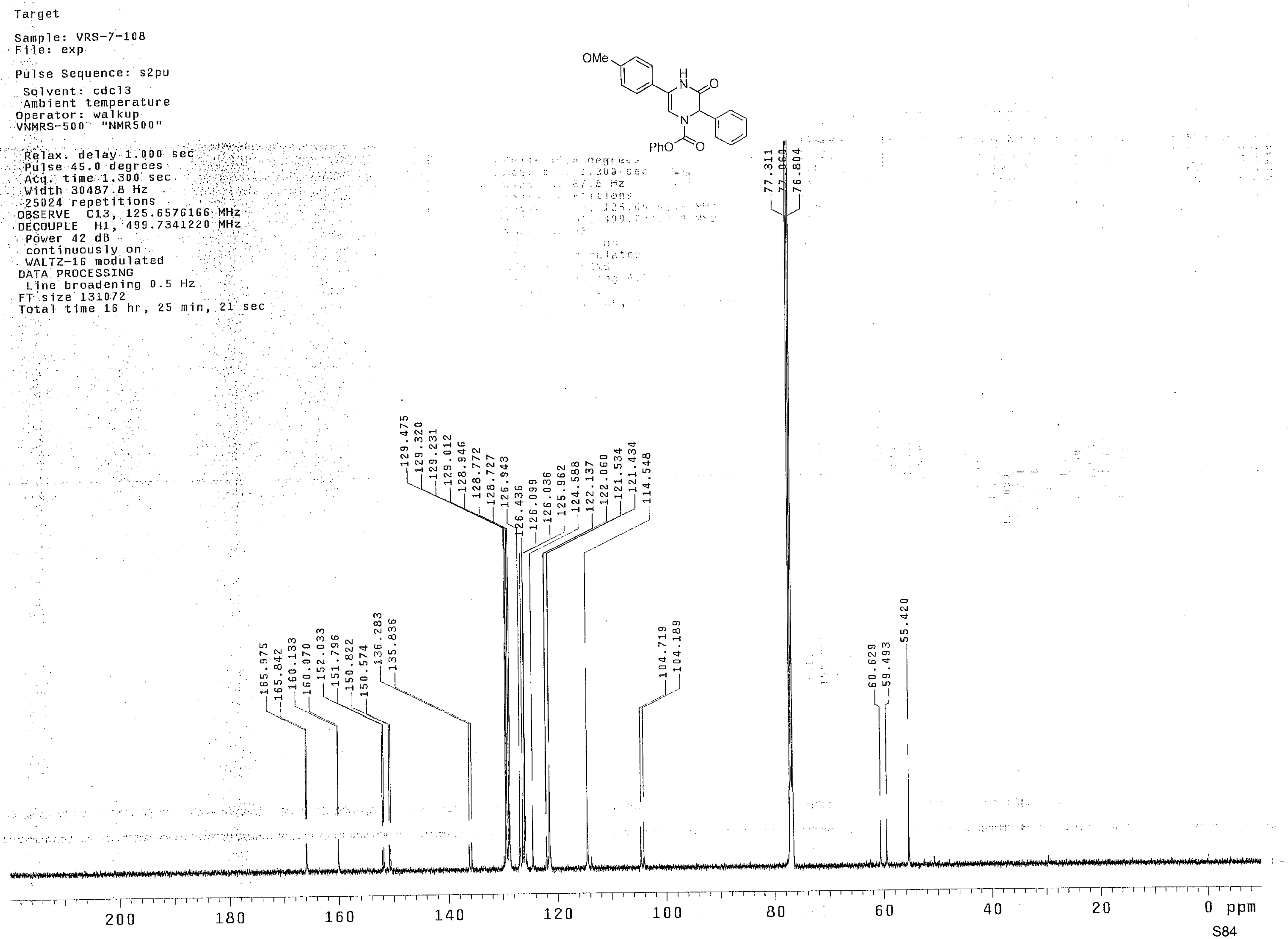

Phenyl 5-ethyl-3-oxo-2-phenyl-3,4-dihydropyrazine-1(2H)-carboxylate (15b)

Sample: DBR-4-615
Sample ID: s_20120725_02
File: s_20120725_02/data

Pulse Sequence: s2pul
Solvent: cdcl3
Temp. 26.0 C / 299.1 K
Operator: walkup
File: cdcl3_01
VNMRS-500 "NMR500"

Relax. delay 1.000 sec
Pulse 45.0 degrees
Acq. time 2.049 sec
Width 8012.8 Hz
8 repetitions
OBSERVE H1, 499.7316285 MHz
DATA PROCESSING
Resol. enhancement -0.0 Hz
FT size 65536
Total time 0 min, 31 sec

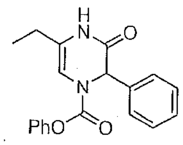

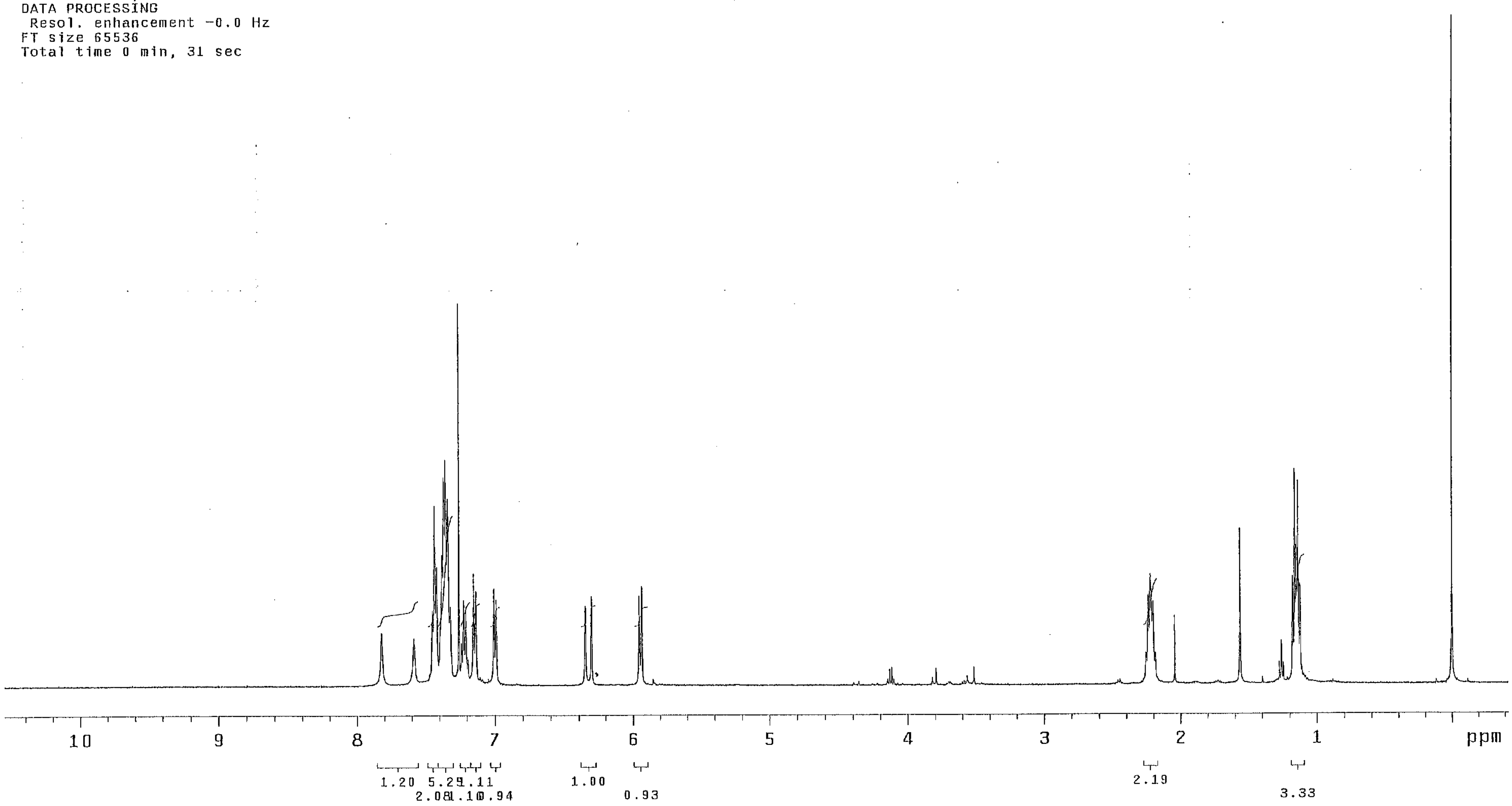

Phenyl 5-ethyl-3-oxo-2-phenyl-3,4-dihydropyrazine-1(2H)-carboxylate (15b)

```
Sample: DBR-4-615
Sample ID: s_20120724_1
File: /home/walkup/vnmr

Pulse Sequence: s2pul
 Solvent: cdcl3
 Temp. 26.0 C / 299.1 K
Operator: walkup
File: cdcl3_01
VNMRS-500  "NMR500"

 Relax. delay 1.000 sec
 Pulse 45.0 degrees
 Acq. time 1.300 sec
 Width 30487.8 Hz
 256 repetitions
OBSERVE  C13, 125.6576352 MHz
DECOUPLE  H1, 499.7341220 MHz
 Power 42 dB
 continuously on
 WALTZ-16 modulated
DATA PROCESSING
 Line broadening 0.5 Hz
FT size 131072
Total time 9 min, 51 sec
```

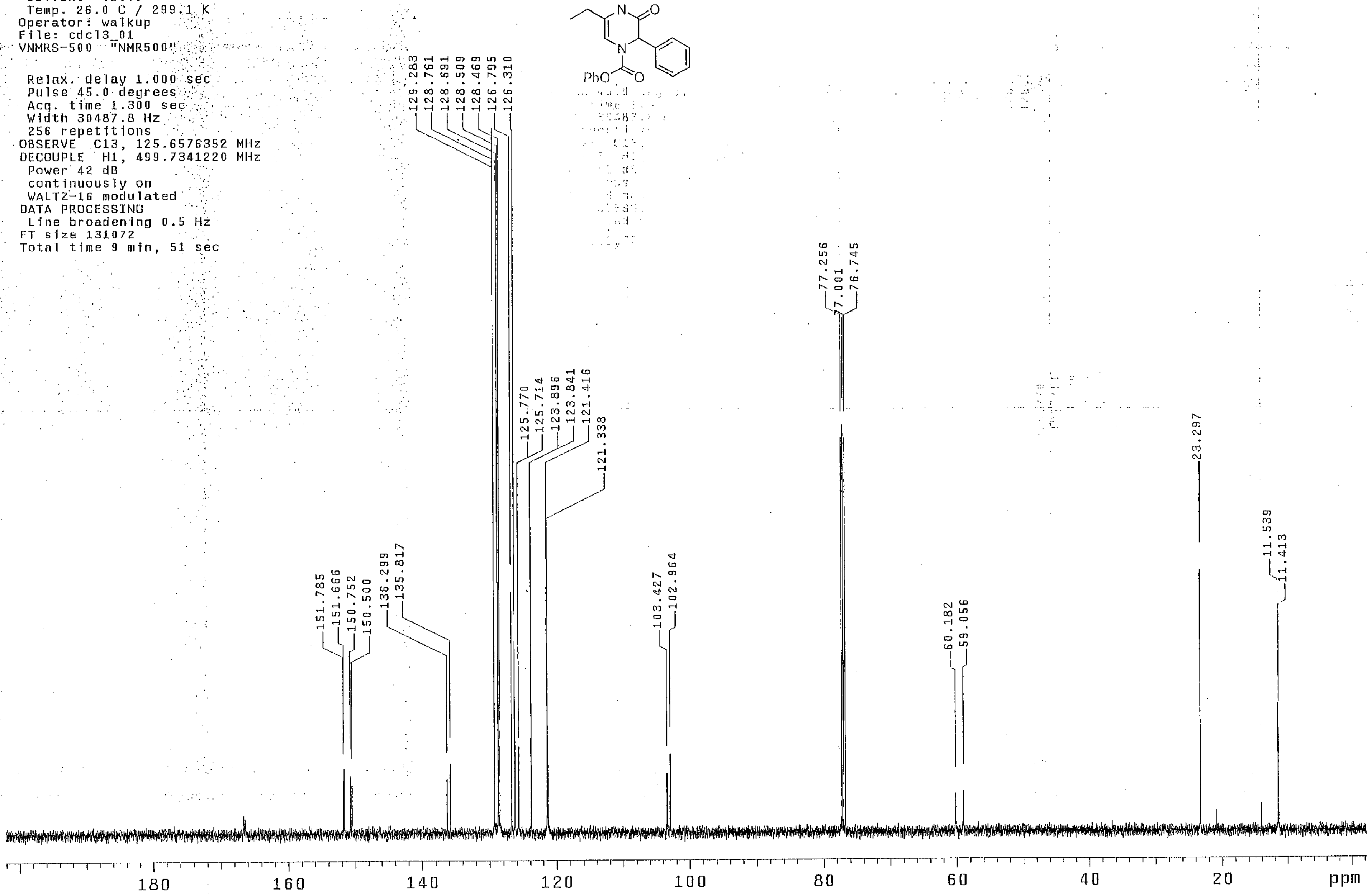

Phenyl 5-benzyl-3-oxo-2-phenyl-3,4-dihydropyrazine-1(2H)-carboxylate (15c)

Sample: DBR-4-635
Sample ID: s_20120905_02
File: s_20120905_02/data/cdcl3

Pulse Sequence: s2pul

Solvent: cdcl3
Temp. 26.0 C / 299.1 K
Operator: walkup
File: cdcl3_01
VNMRS-500 "NMR500"

Relax. delay 1.000 sec
Pulse 45.0 degrees
Acq. time 2.049 sec
Width 8012.8 Hz
8 repetitions
OBSERVE H1, 499.7316473 MHz
DATA PROCESSING
Resol. enhancement -0.0 Hz
FT size 65536
Total time 0 min, 31 sec

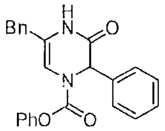

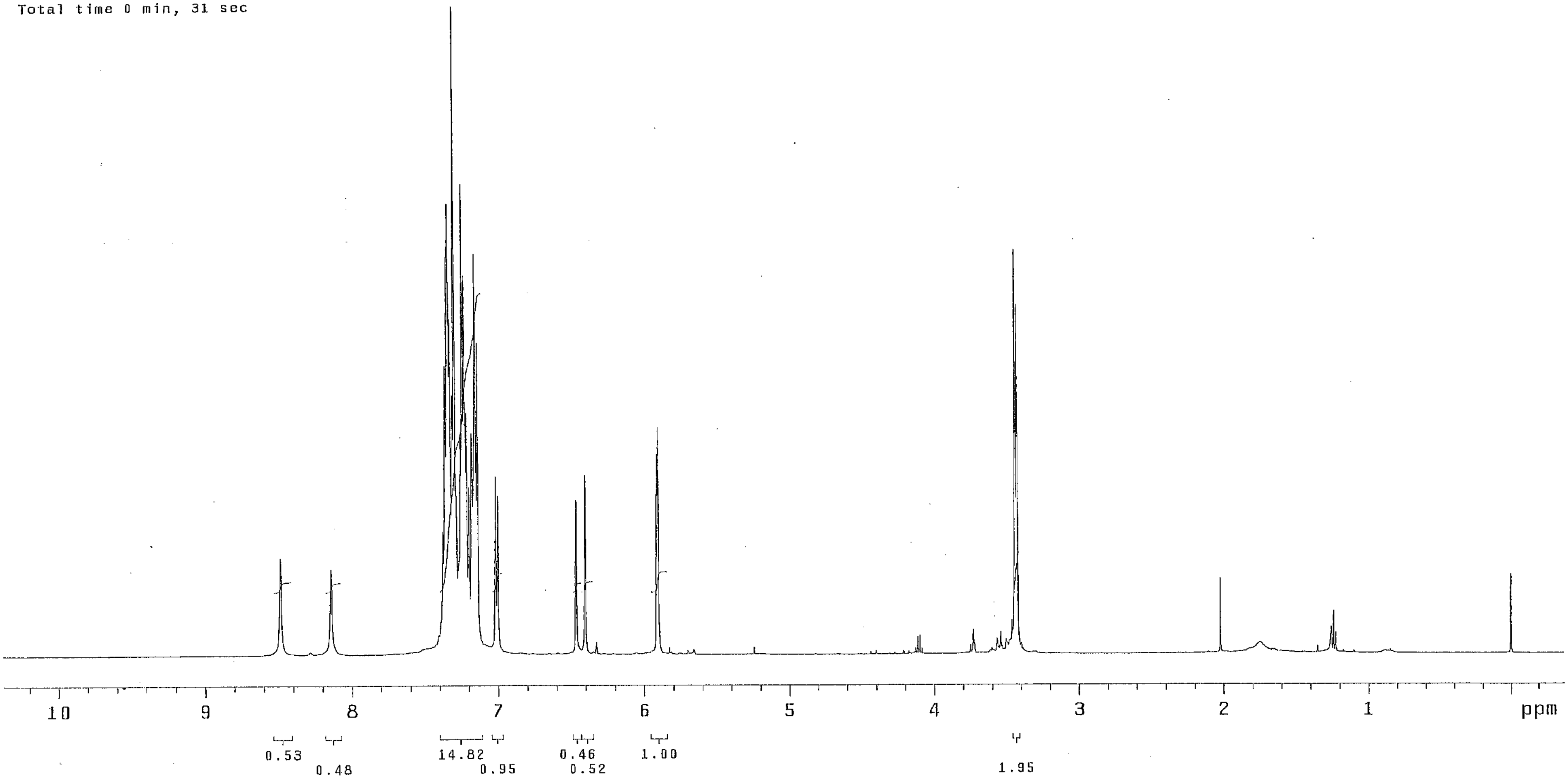

Phenyl 5-benzyl-3-oxo-2-phenyl-3,4-dihydropyrazine-1(2H)-carboxylate (15c)

Sample: DBR-4-635
Sample ID: s_20120905_03
File: s_20120905_03/data/cdcl3_01.

Pulse Sequence: s2pul

Solvent: cdcl3
Temp. 26.0 C / 299.1 K
Operator: walkup
File: cdcl3_01
VNMRS-500 "NMR500"

Relax. delay 1.000 sec
Pulse 45.0 degrees
Acq. time 1.300 sec
Width 30487.8 Hz
256 repetitions
OBSERVE C13, 125.6576314 MHz
DECOUPLE H1, 499.7341220 MHz
Power 42 dB
continuously on
WALTZ-16 modulated
DATA PROCESSING
Line broadening 0.5 Hz
FT size 131072
Total time 9 min, 51 sec

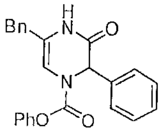

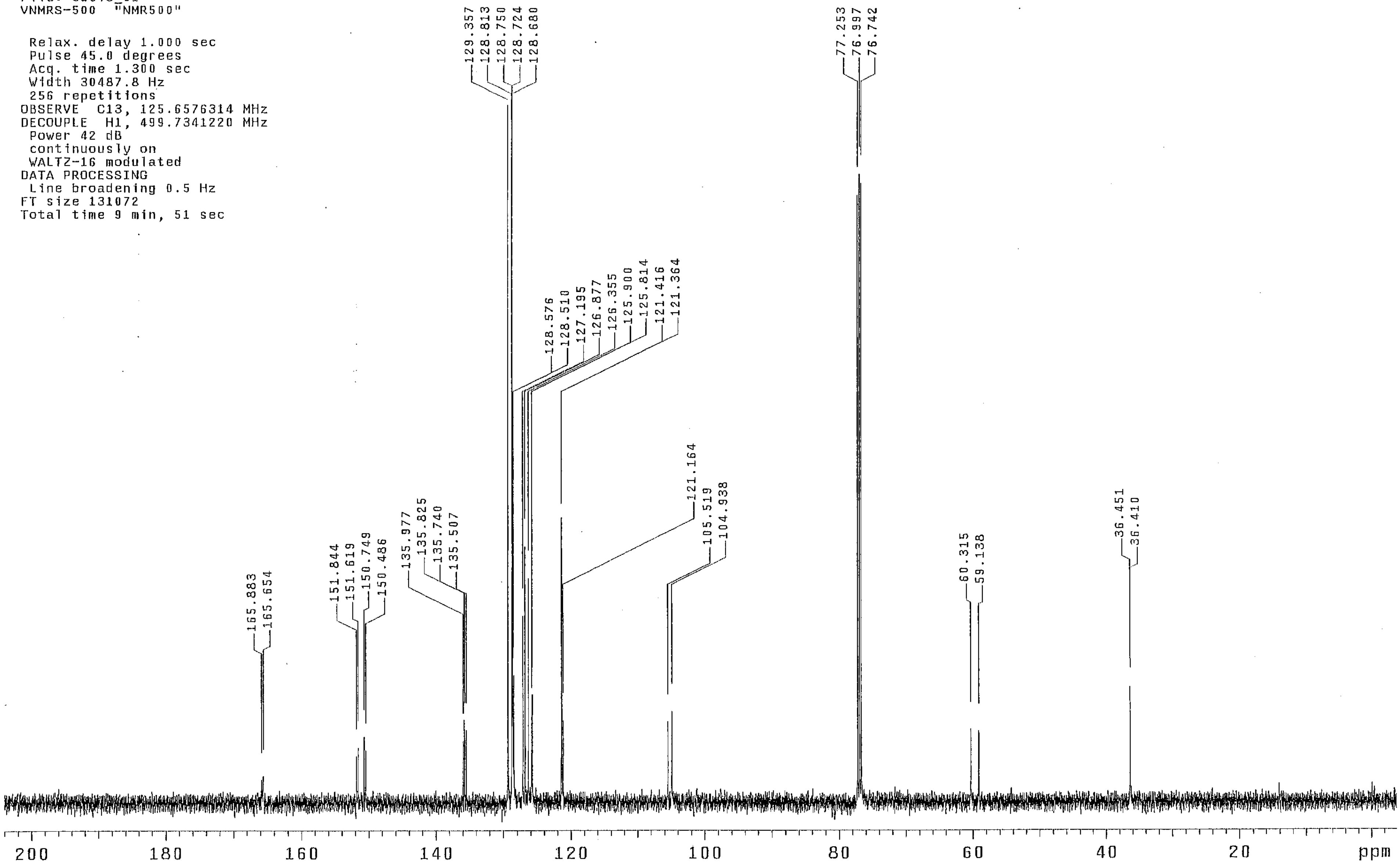

Sample: DBR-4-599R
Sample ID: s_20140703_01
File: s_20140703_01/data/cdcl3_01.fid

Pulse Sequence: s2pul

Solvent: cdcl3
Temp. 26.0 C / 299.1 K
Operator: walkup
File: cdcl3_01
VNMRS-500 "NMR500"

Relax. delay 1.000 sec
Pulse 45.0 degrees
Acq. time 2.049 sec
Width 8012.8 Hz
8 repetitions
OBSERVE H1, 499.7316290 MHz
DATA PROCESSING
Resol. enhancement -0.0 Hz
FT size 65536
Total time 0 min, 31 sec

Phenyl 3-oxo-2,5-diphenyl-3,4-dihydropyrazine-1(2H)-carboxylate (15d)

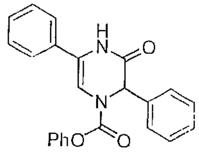

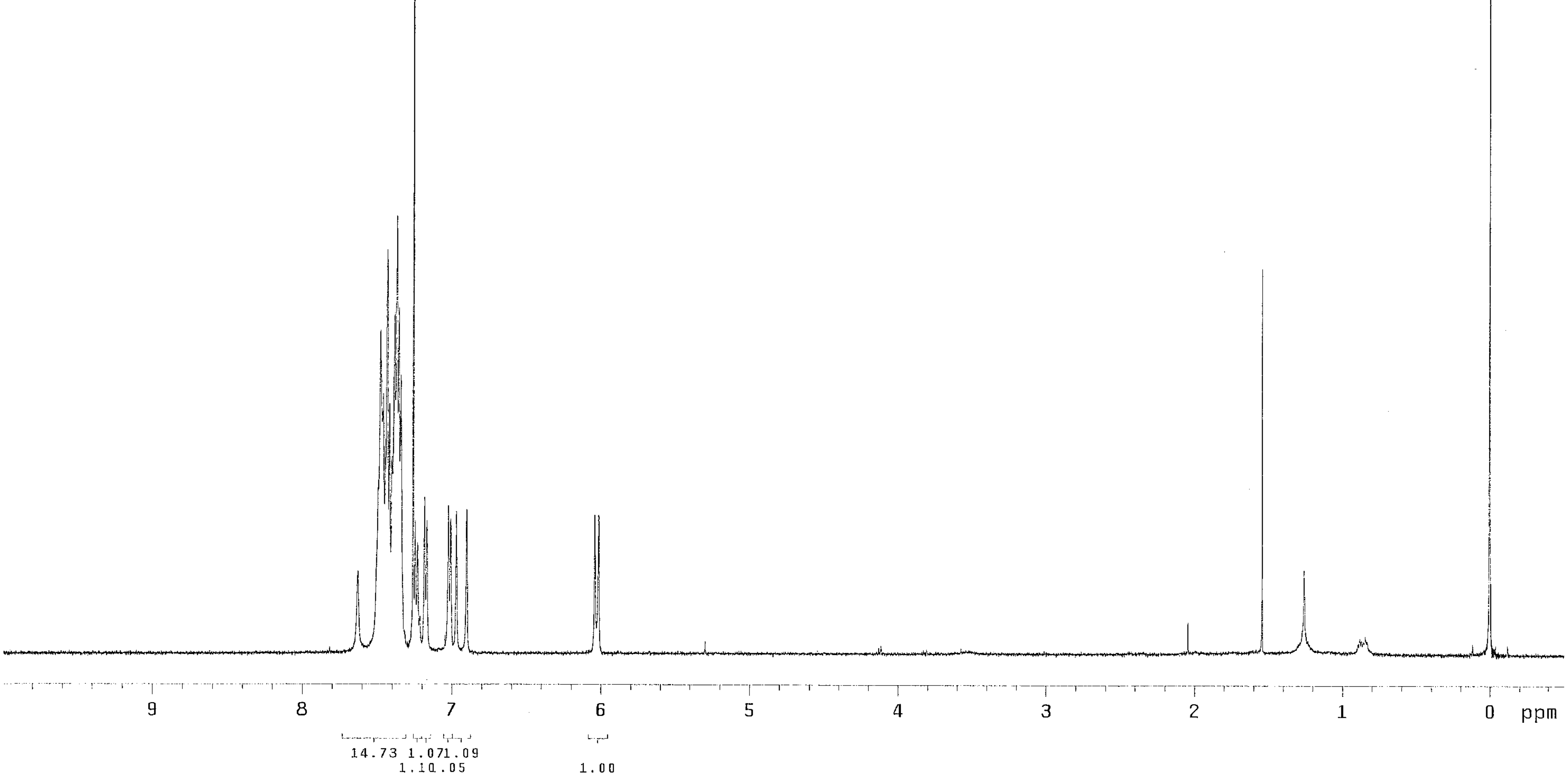

Phenyl 3-oxo-2,5-diphenyl-3,4-dihydropyrazine-1(2H)-carboxylate (15d)

Sample: DBR-4-599R
File: exp

Pulse Sequence: s2pul
Solvent: cdcl3
Temp. 26.0 C / 299.1
Operator: walkup
VNMRS-500 "NMR500"

Relax. delay 1.000 sec
Pulse 45.0 degrees
Acq. time 1.300 sec
Width 30487.8 Hz
25152 repetitions
OBSERVE C13, 125.6576166 MHz
DECOUPLE H1, 499.7341220 MHz
Power 42 dB
continuously on
WALTZ-16 modulated
DATA PROCESSING
Line broadening 0.5 Hz
FT size 131072
Total time 16 hr, 25 min, 21 sec

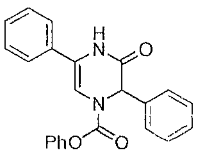

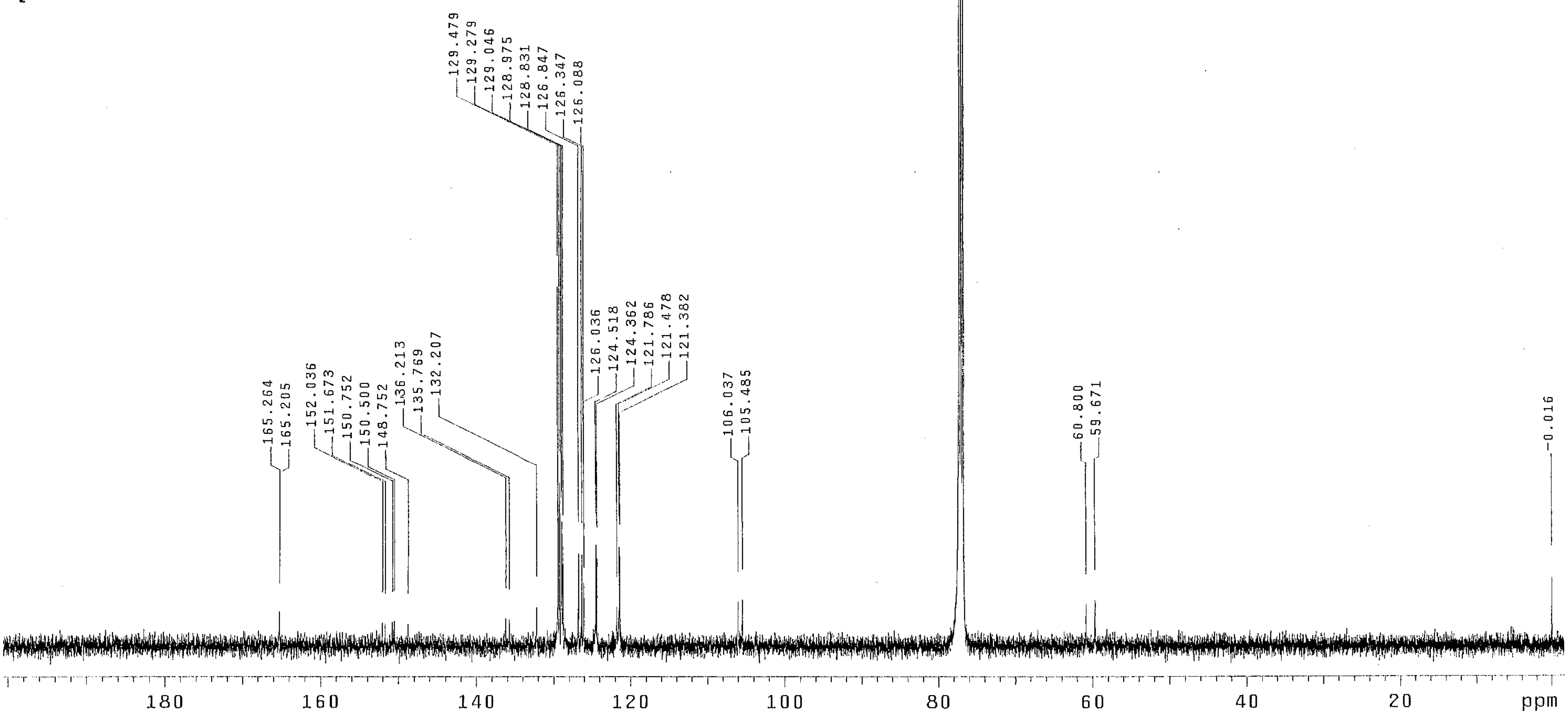

Methyl 2-((2-oxoethyl)(phenoxycarbonyl)amino)-2-phenylacetate (16)

DBR-5-856

Sample Name	**DBR-5-856**	Pulse sequence	**PROTON**	Temperature	**25**	Study owner	**walkup**
Date collected	**2017-09-01**	Solvent	**cdcl3**	Spectrometer	**NMR500-vnmrs500**	Operator	**walkup**

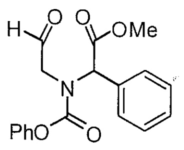

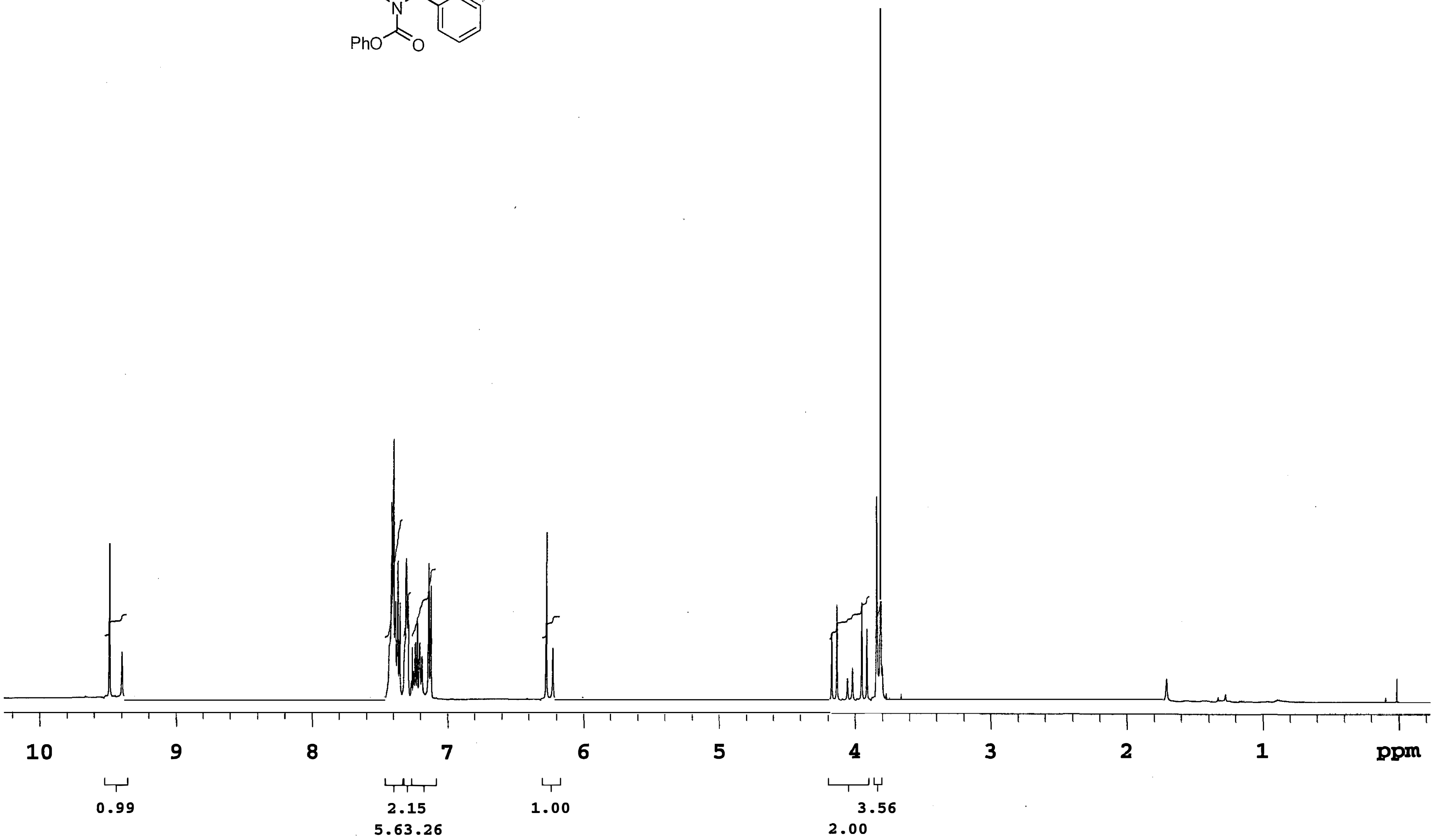

Data file /home/walkup/vnmrsys/data/DBR-5-856_20170901_07/PROTON_01

Plot date 2017-09-01

Methyl 2-((2-oxoethyl)(phenoxycarbonyl)amino)-2-phenylacetate (16)

DBR-5-856

Sample Name	**DBR-5-856**	Pulse sequence	**CARBON**	Temperature	**25**	Study owner	**walkup**
Date collected	**2017-09-01**	Solvent	**cdcl3**	Spectrometer	**NMR500-vnmrs500**	Operator	**walkup**

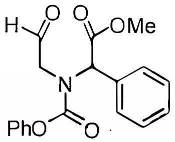

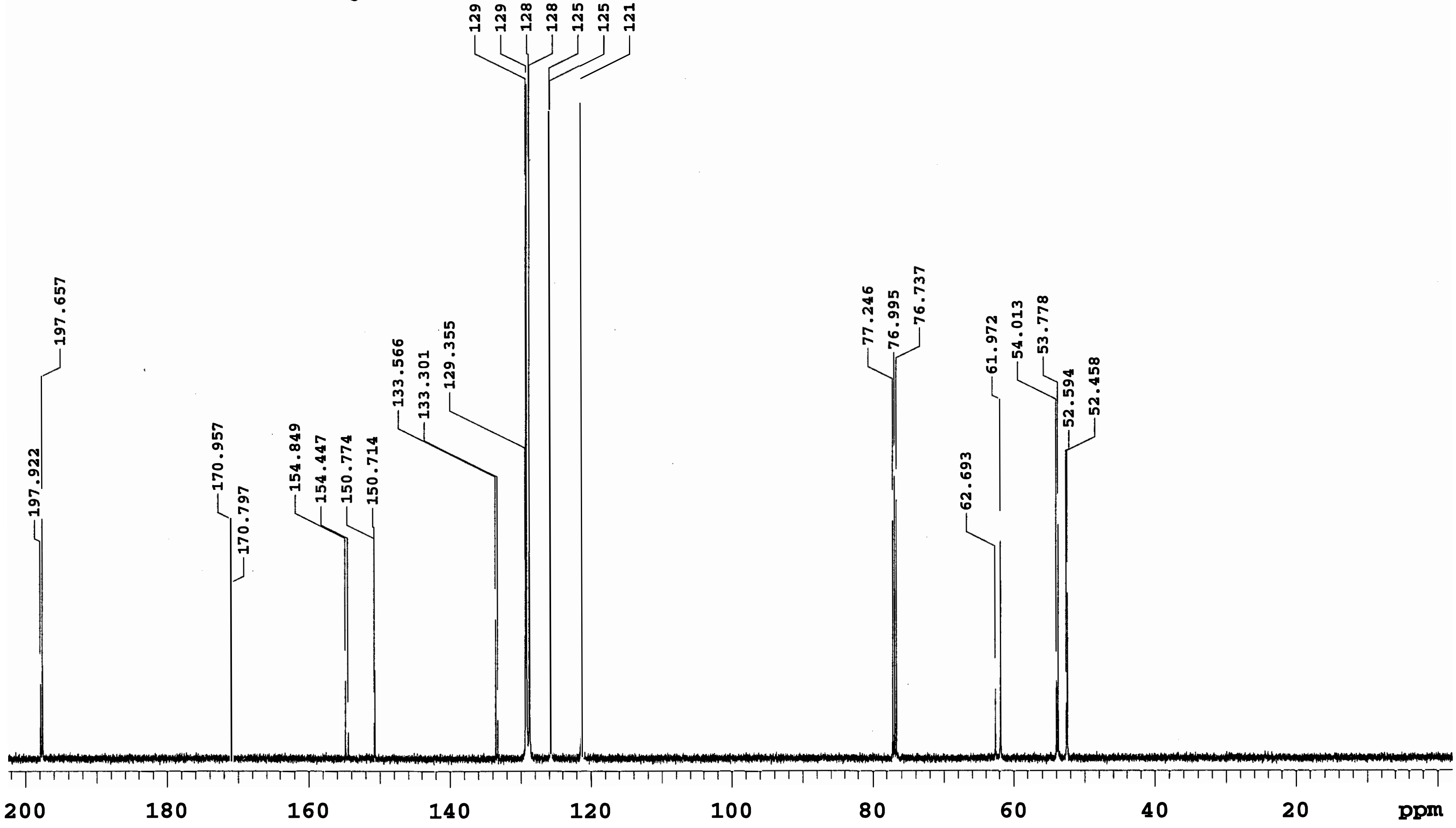

Data file /home/walkup/vnmrsys/data/DBR-5-856_20170901_08/CARBON_01

Plot date 2017-09-01
